# Cross-national research on adolescent mental health: a systematic review comparing research in low, middle and high-income countries

**DOI:** 10.1136/bmjgh-2025-019267

**Published:** 2025-07-25

**Authors:** Xiao Zhang, Yuko Mori, Anne Abio, Zahra Kafami Khorasani, Sonja Gilbert, Meytal Grimland, Nikita Bezborodovs, Wan Mohd Azam Wan Mohd Yunus, Sanju Silwal, Minja Westerlund, Samir Kumar Praharaj, David M Ndetei, Hitoshi Kaneko, Emmi Heinonen, Andre Sourander

**Affiliations:** 1 Department of Child Psychiatry, Research Centre for Child Psychiatry, University of Turku, Turku, Finland; 2 INVEST Research Flagship Center, University of Turku, Turku, Finland; 3 Shalvata Mental Health Center, Hod Hasharon, Israel; 4 Department of Psychiatry and Narcology, Riga Stradins University, Riga, Latvia; 5 Child and Youth Mental Health Centre, Children’s Clinical University Hospital, Riga, Latvia; 6 Faculty of Social Sciences and Humanities, Universiti Teknologi Malaysia, Johor, Malaysia; 7 Department of Psychiatry, Kasturba Medical College, Manipal, India; 8 Manipal Academy of Higher Education, Manipal, India; 9 Africa Institute of Mental and Brain Health, Nairobi, Kenya; 10 Department of Psychiatry, University of Nairobi, Nairobi, Kenya; 11 Psychological Support and Research Center for Human Development, Nagoya University, Nagoya, Japan; 12 Department of Child Psychiatry, University of Turku and Turku University Hospital, Turku, Finland

**Keywords:** Systematic review, Mental Health & Psychiatry, Global Health, Epidemiology, Public Health

## Abstract

**Background:**

Although 90% of the global adolescent population resides in low and middle-income countries (LMICs), cross-national mental health research has primarily focused on high-income countries. This systematic review aims to examine cross-national studies, including LMICs, focusing on adolescent mental health and psychosocial problems.

**Methods:**

Peer-reviewed articles comparing adolescent mental health or psychosocial issues across countries were included if published in English and based on observational study designs. Six databases were searched from inception to January 2024. Risk of bias was assessed using the JBI tool and additional cross-national quality criteria. A narrative synthesis was conducted following the Synthesis Without Meta-analysis guidelines.

**Results:**

The search identified 172 studies including data from over 12 million adolescents across 166 countries. The most used international survey was the Health Behaviour in School-aged Children study, and most studies employed cross-sectional designs with school-based, self-reported questionnaires. Adolescent mental health research involved 166 countries, while 52 countries were not represented in any of the studies. The inclusion of LMICs in cross-national research increased over time. Internalising problems and bullying were well-researched topics, but studies on externalising behaviours were limited. The number of publications increased over time, particularly after 2020. Variations by income level were observed, with traditional bullying more prevalent in LMICs.

**Discussion:**

This review provides important insights into global patterns and gaps in adolescent mental health research. It highlights the need to address methodological heterogeneity, including the use of validated and culturally adapted measures, comprehensive reporting of sampling procedures, sample sizes, response rates and greater attention to generalisability. In addition, the targeted inclusion of under-represented regions is essential to addressing global disparities and informing effective interventions.

**PROSPERO registration number:**

CRD42024505077.

**WHAT IS ALREADY KNOWN ON THIS TOPIC**

**WHAT THIS STUDY ADDS**

- This review provides a comprehensive overview of cross-national adolescent mental health research, with a focus on low and middle-income countries (LMICs), identifying trends in study design, country representation and topic coverage. Most studies use cross-sectional, self-reported questionnaires, but there is a lack of validated measures. While LMIC inclusion has increased over time in the last 30 years, 52 countries remain unrepresented. Internalising problems and bullying are well-researched, whereas externalising behaviours receive less attention.

**HOW THIS STUDY MIGHT AFFECT RESEARCH, PRACTICE OR POLICY.**

- The findings emphasise the need for culturally adapted and validated mental health measures. Addressing research gaps in under-represented regions and research topics can improve the global understanding of adolescent mental health.

## Introduction

 Adolescents face a range of mental health challenges that can affect their well-being, development and transition into adulthood.^1^ Approximately one-third of mental disorders begin before the age of 14, with onset peaking at 14.5 years, and nearly half appear before the age of 18.^2^ In recent decades, reviews have suggested that adolescent mental health has become a growing public health concern globally.^3 4^ Since adolescents are still of school age, they are significantly influenced by their school environments. Therefore, it is essential to account for school-related risk factors such as bullying, body image concerns, loneliness, learning disabilities, peer problems and academic stress when studying their mental health.^5 6^

Mental health issues among adolescents in low and middle-income countries (LMICs) can be affected by various factors, including socioeconomic instability, cultural stigma surrounding mental health, inadequate access to mental healthcare services and exposure to violence or traumatic experiences.^37^,^9^ Despite hosting 90% of the world’s adolescents, LMICs receive significantly less research attention and resources for adolescent mental health compared with high-income countries (HICs).^9^,^11^ This has led to several issues, such as reliance on convenience sampling, the use of questionnaires developed in HICs that are not adequately adapted for LMICs and a lack of methodological rigour necessary to ensure high-quality research. Furthermore, only 2% of global mental health data have been collected specifically on children and adolescents in LMICs.^12^ Due to this limitation, many reviews rely heavily on data from HICs.^7 9 13^ To better understand adolescent mental health in LMICs and to enable meaningful comparisons with HICs, it is important to examine the methodologies used in existing cross-national studies that include LMICs and to identify overarching patterns.

There are a few large-scale, cross-national surveys that provide valuable insights into health-related behaviours and mental health outcomes, such as the Health Behaviour in School-aged Children (HBSC) study, the Global School-based Student Health Survey (GSHS) and the Programme for International Student Assessment (PISA). The HBSC, established in 1982 and supported by WHO, collects data on health-related behaviours among young people aged 11, 13 and 15 years across 51 countries in Europe and North America.^14^ The GSHS, developed in 2003 by WHO and the US Centers for Disease Control and Prevention, is the largest adolescent mental health survey, covering over 100 countries, mainly LMICs, and it focuses on health risk and protective factors for ages 13–17.^15^ PISA has been conducted by the Organisation for Economic Co-operation and Development every 3 years since 2000 to examine the academic knowledge and skills of 15-year olds.^16^ These large international surveys serve as key resources in understanding the global patterns of adolescent mental health and in identifying gaps in research, especially in under-represented regions.

This systematic review follows a narrative synthesis approach. The aim of this systematic review is to examine the existing cross-national research on adolescent mental health and psychosocial issues, with a focus on LMICs. Our research questions are:

1. What are the key international surveys that have collected data on adolescent mental health across countries, what comparative methodologies or frameworks have they employed and how have these evolved over time in response to changing global research priorities?
2. Which countries have been included in cross-national studies on adolescent mental health, which are underrepresented, and how has the geographical coverage of these studies shifted over time?
3. What have the research priorities been in cross-national studies on adolescent mental health, how have these priorities developed over time and to what extent do the findings differ across countries by income level?

This is the first systematic review to comprehensively evaluate cross-national studies on adolescent mental health, explicitly addressing both high- and low-income settings, with a focus on evolving research methodologies and global inclusivity.

## Method

### Search strategy and selection criteria

This systematic review was conducted following the Preferred Reporting Items of Systematic Reviews and Meta-analyses.^17^ The review protocol was registered with the International Prospective Register of Systematic Reviews (PROSPERO, registration number CRD42024505077). A comprehensive search was conducted across six electronic databases (Web of Science, CINAHL, PubMed, EMBASE, PsycINFO and PsycArticles) from database inception up to the end of January 2024. For each search term, Medical Subject Headings (MeSH) terms or free-text terms were identified and then adapted for the other databases. The websites of international surveys were screened for potential papers. Reference lists of relevant articles were also searched for additional publications. The search strings consisted of a combination of 77 different terms, 49 of which referred to mental health and psychosocial issues, 20 to adolescents and 8 were cross-national terms (see online supplemental table 1 for the full-search strategy).

Eligible studies were defined as peer-reviewed empirical articles that met the following criteria: (a) included a sample of adolescents aged 10–19 years, with a flexibility of ±2 years (studies including participants outside this age range were excluded unless findings were reported separately for the target age group); (b) included a sample consisting of the general population (studies including clinical samples were excluded); (c) considered mental health and psychosocial issues, including emotional problems, emotional dysregulation, mood disorder, depression, anxiety, stress, affective disorder, behavioural problems, conduct problems, disruptive behaviour, internalising problems, externalising problems, suicide behaviour, non-suicidal self-injury, self-harm, self-injury, body image, eating problems, eating disorder, attention deficit, hyperactivity, attention deficit hyperactivity disorder, attention deficit disorder, autism, autism spectrum disorder, psychological trauma, post-traumatic stress disorder, loneliness, social isolation, peer problems, sleep problems and bullying; (d) were observational studies such as cross-sectional, longitudinal, cohort studies, or ecological studies comparing at least two or more countries (including at least one LMIC at the year of data collection based on World Bank country income level classification) and (e) were published in the English language. Studies including only HICs were excluded, as our aim was to explore cross-national comparisons that involve at least one LMIC in order to understand research participation and disparities in these settings. Single-country studies were excluded because the aim of this review was to examine country representation in cross-national mental health research using comparable designs.^18^ Additionally, in this systematic review, the term ‘country’ does not necessarily denote political independence but refers to any territory with separate results in original studies or distinct income classification by the World Bank.^18^

### Data extraction and synthesis

Articles retrieved from databases were first exported to Mendeley citation manager and subsequently imported into Covidence,^19^ a web-based software platform for systematic reviews. Title and abstract screening and full-text screening were performed by four reviewers (XZ, YM, AA and ZKK), and each article was independently screened by at least two reviewers. Any disagreements between the two reviewers were resolved by a third reviewer (SG or MG). Each paper included in the review was assigned to one of the four reviewers (XZ, YM, AA and ZKK) for data extraction. The primary reviewer extracted the data, and a second reviewer checked and verified the extracted information. Extracted information included the authors and year of publication, the number of countries, the measures used, the sample characteristics and the key findings. Given the substantial variability in focus, methodology and variables measured, we decided against a meta-analysis of rates and instead conducted a narrative synthesis according to the Synthesis Without Meta-analysis guidelines.^20^ No statistical model was applied due to the narrative nature of the synthesis and the heterogeneity of the included studies. Findings were grouped by mental health domains (eg, internalising, externalising, bullying), country income classification (HICs vs LMICs) and publication year. These classification categories were defined prior to synthesis, based on the review objectives and existing literature.

### Quality assessment

Quality ratings were conducted by the same reviewers independently using the Joanna Briggs Institute (JBI) critical appraisal tools for prevalence studies.^21^ The JBI critical appraisal tool has nine items to evaluate sampling methods, study objectives and settings, the representativeness of the sample, the reliability and validity of the measures and the data analysis method. The authors developed four additional items to assess key quality domains specific to cross-national research. This development involved consulting researchers experienced in cross-national studies and reviewing existing scientific literature on the subject. The additional items focused on research design comparability across countries, cultural appropriateness and conceptual equivalence of measures, translation and adaptation of survey instruments, and the appropriateness of analytical approaches (see online supplemental table 2 for the full details and results of the quality assessment). Each study was rated by two independent reviewers, and disagreements were resolved through discussion or by involving a third reviewer. Although no statistical correction for risk of bias was applied, potential methodological limitations such as low or unreported response rates, non-representative sampling and lack of validated measures were acknowledged and considered in the interpretation of findings.

### Patient and public involvement

This research was done without patient and public involvement.

## Result

### Description of the studies

**Figure 1:**
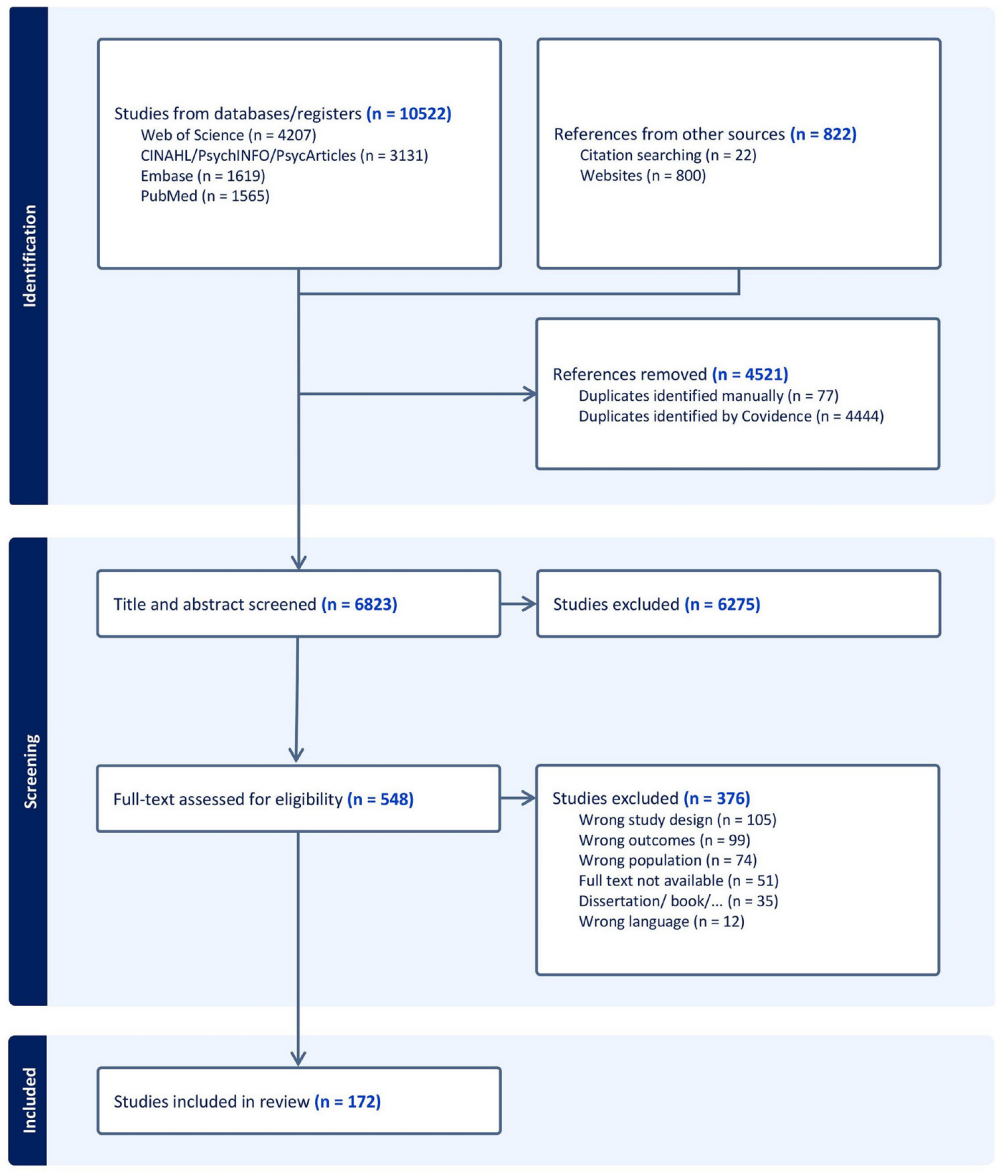
PRISMA flow diagram of database search and record screening. PRISMA, Preferred Reporting Items for Systematic Reviews and Meta-Analyses.

The electronic searches identified 10 522 articles along with 822 additional articles retrieved from other sources. After the removal of 4521 duplicates, 6823 articles remained for screening. Title and abstract screening excluded 6275 articles, leaving 548 articles for full-text evaluation. Of these, 376 were excluded as they did not meet the inclusion criteria, including some studies for which full texts could not be retrieved. Due to resource constraints, we did not contact authors for the missing full texts. Finally, 172 articles met the inclusion criteria and were included in the review (figure 1).

Out of the 172 articles included in the review, 12.2% focused exclusively on LMICs (table 1). The surveys most often used were the HBSC (19.8%), the GSHS (12.2%) and PISA (8.1%). The most studied domain was internalising problems (51.2%), followed by bullying (34.3%). Cross-sectional surveys were the most common research design (88.4%), with most studies (90.1%) based on school-based questionnaires. Self-reports were the predominant informant source (90.1%), with fewer studies using self-reports and parent/caregiver-reports (8.1%) or other informants such as teachers or peers (1.7%). Included studies covered 2–88 countries (mean=15.94, median=5.5, IQR: 2–26.5), with 62.2% involving between 2 and 9 countries. The quality assessment tool revealed that many studies had low response rates or did not report response rates (n=74, 43.0%), which they did not employ an appropriate sampling method (n=47, 27.3%), which they lacked detailed descriptions of translation and adaptation procedures for survey instruments (n=42, 24.4%), or which they failed to ensure the cultural appropriateness and conceptual equivalence of the measures used (n=28, 16.3%) (see online supplemental table 2). There were only four papers that conducted measurement invariance testing.

**Table 1:** Characteristics of studies included in the review

	Number of participants	Number of studies	% of included studies
Comparisons			
Both HIC(s) and LMIC(s)	12 340 775	151	87.8
Only LMICs	367 420	21	12.2
Survey name			
Health Behaviour in School-Aged Children	4 965 538	34	19.8
Global School-based Student Health Survey	2 748 932	21	12.2
Programme for International Student Assessment	4 040 099	14	8.1
Global Early Adolescent Study	32 385	4	2.3
The International Survey of Children’s Well-Being	135 233	3	1.7
European School Survey Project on Alcohol and Other Drugs	45 086	2	1.2
Others	5 288 775	94	45.3
Studied domain*			
Internalising problems	4 819 687	88	51.2
Externalising problems	91 372	24	14.0
General mental health and well-being	905 852	29	16.9
Bullying	5 739 831	59	34.3
Research design			
Cross-sectional	11 916 508	156	88.4
Longitudinal	582 249	11	8.1
Repeated cross-sectional	209 438	5	3.5
Methodology			
School-based questionnaire	12 622 155	158	90.1
Community-based questionnaire	34 437	5	3.5
Multiple methodology†	44 041	7	4.1
Unknown	7562	2	2.3
Informants			
Self-report	12 642 396	155	90.1
Self-report and parent/caregiver-report	64 162	14	8.1
Others (teachers, peer)	1637	3	1.7
Number of countries included			
2–9	720 274	107	62.2
10–49	7 867 051	52	30.2
≥50	4 120 870	13	7.6

* Internalising problems: anxiety, depression, suicidal behaviour, self-harm, emotional and internalising problems, body image, loneliness, sleep difficulties, post-traumatic stress and eating problems. Externalising problems: externalising problems and conduct problems. General mental health and well-being: general mental health, subjective well-being and stress. Bullying: traditional, cyber and sexual bullying

† Multiple methodology refers to the use of more than one methodology within a single study (eg, collecting data from children, using a school-based questionnaire and from their parents, via a household survey).

HIC, high-income country; LMIC, low and/or middle-income country.

### Geographical representation in cross-national adolescent mental health research

**Figure 2:**
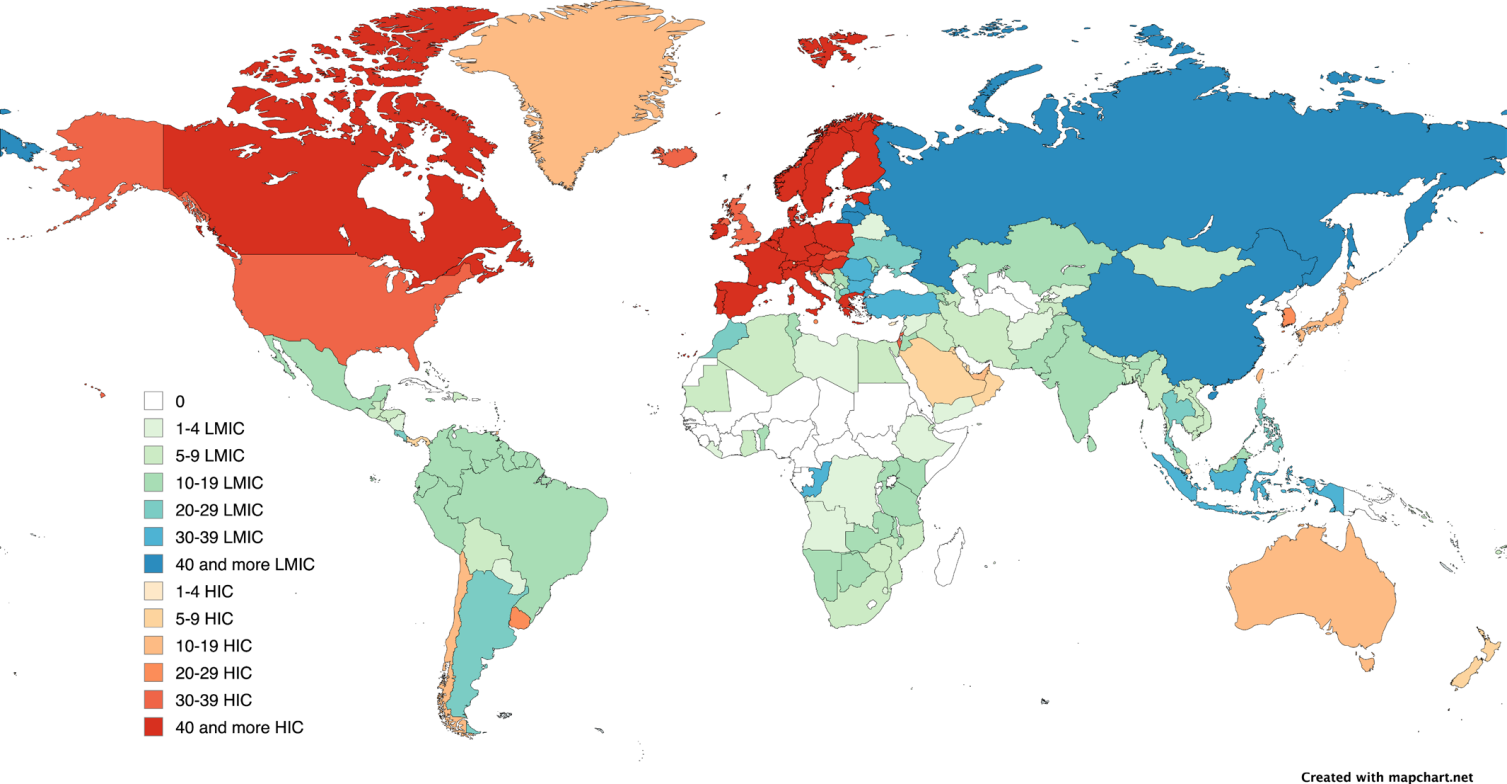
Global distribution of the included studies by country. The term 'country' does not necessarily denote political independence but refers to any territory with separate results in original studies or distinct income classification by the World Bank.^18^ HIC, high-income country; LMIC, low and/or middle-income country.

Out of the 218 countries recognised by the World Bank,^18^ 52 countries were not represented in any of the studies included in this review (figure 2) (see online supplemental table 3 for the full list of countries). The excluded countries were primarily from Sub-Saharan Africa (n=27) followed by the Pacific region (n=8). Thirty-six countries, mostly from Eastern Europe, were included in the review under both LMIC and HIC classifications due to shifts in economic categorisation by the World Bank across data collection periods. Among the HICs, Belgium appeared in the highest number of studies (n=70), followed by Germany and Italy, each included in 56 studies. The LMIC with the highest number of studies (n=61) was China, followed by Russia (n=47) and Indonesia (n=34).

**Figure 3:**
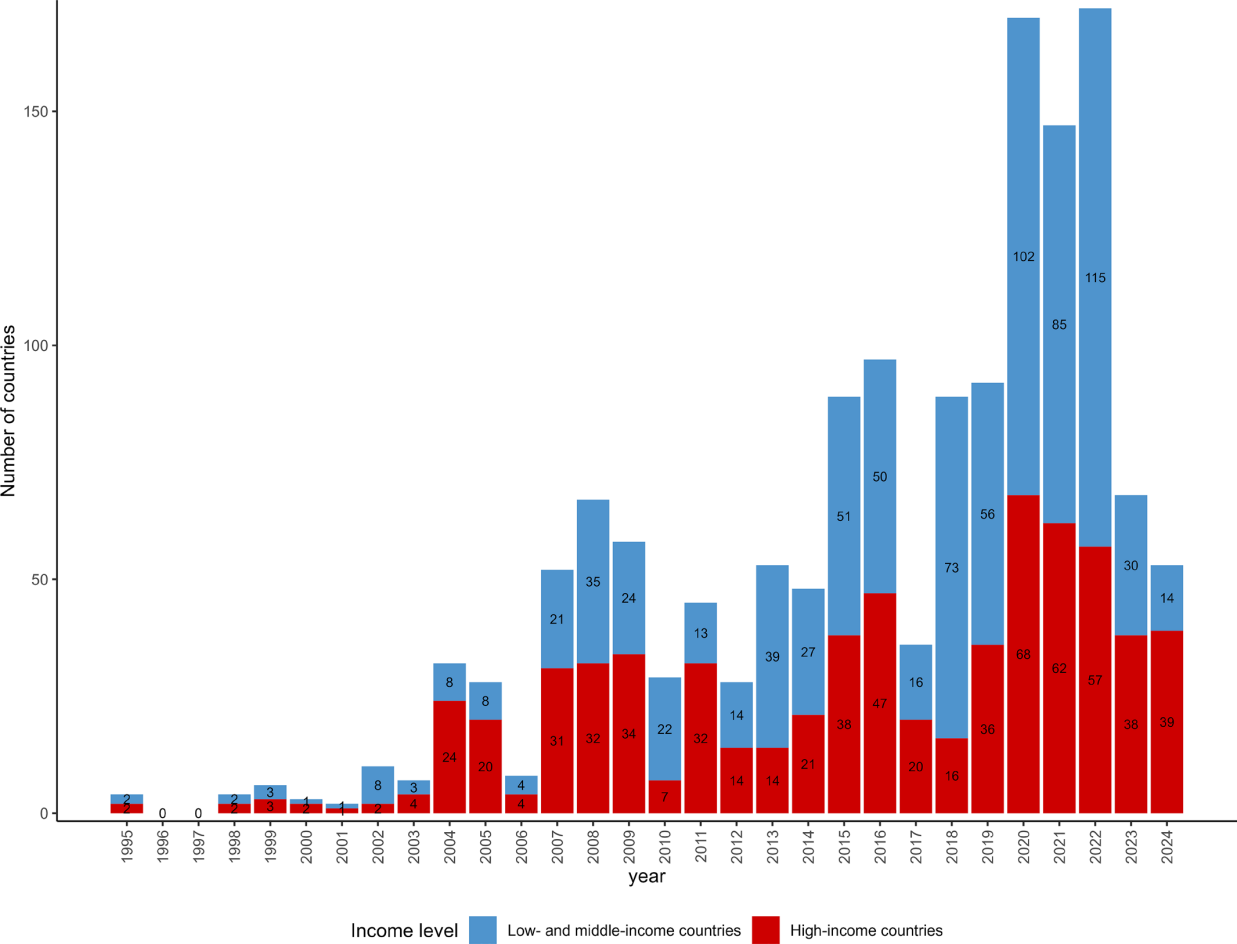
Number of countries included in cross-national studies including at least one low or middle-income country.

Figure 3 shows the number of countries included in previous cross-national research that involved at least one LMIC. The figure highlights the growing inclusion of LMICs in adolescent mental health research, especially within the last decade. From 1995 to the early 2000s, the number of cross-national studies including LMICs was low, with fewer than 10 LMICs involved per year. A significant increase began in the mid-2000s, with notable peaks in 2007–2009. A second increase occurred after 2015, with the years 2020–2022 having the highest number of LMICs included in studies on adolescent mental health research.

### Distribution of research topics in cross-national adolescent mental health studies over the past 30 years and key findings

**Figure 4:**
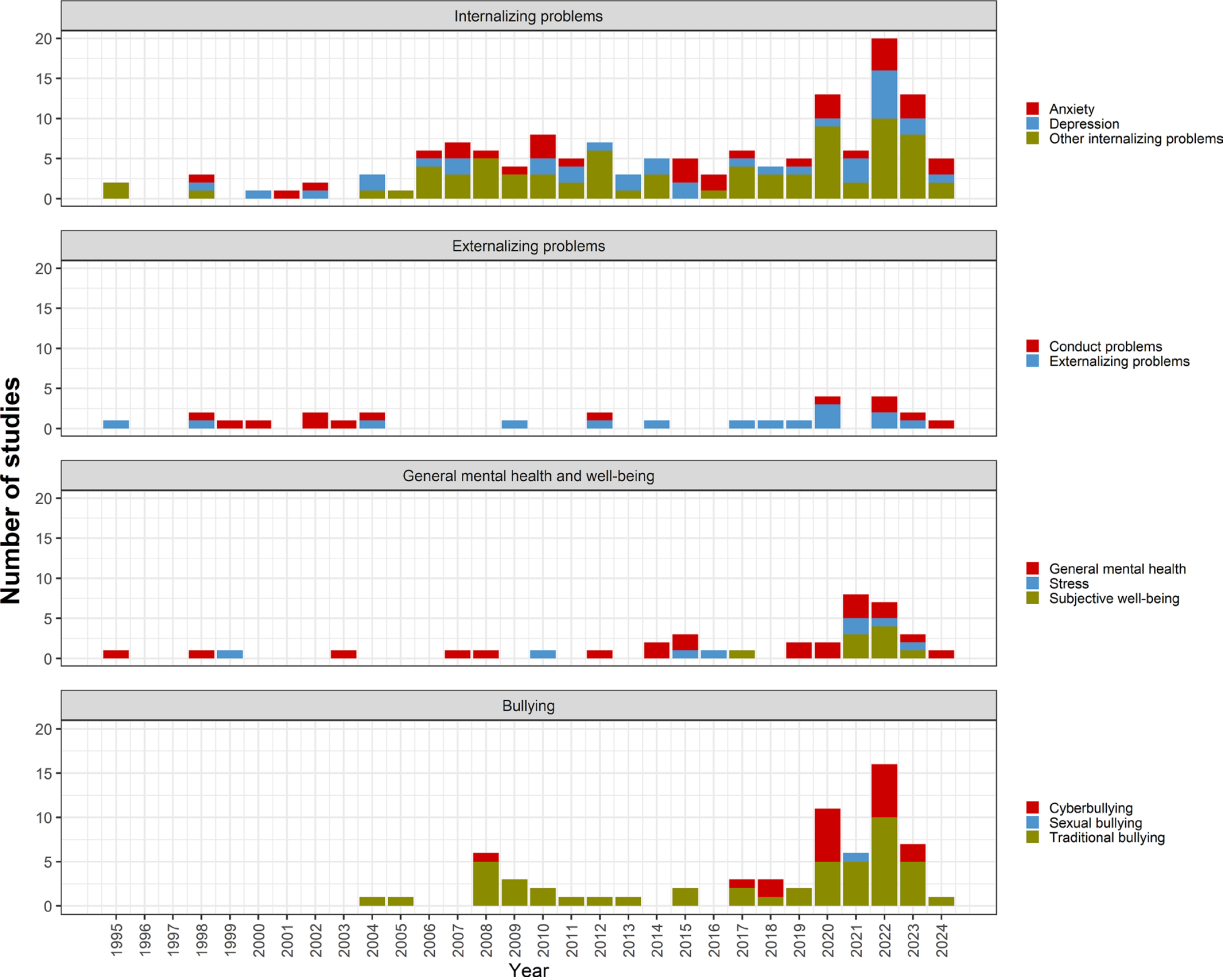
The number of cross-national studies in the past 30 years by mental health problems.

Figure 4 shows the number of cross-national studies on adolescent mental health that include LMICs. Mental health and psychosocial problems are categorised into four mental health problem domains: internalising problems (anxiety, depression, suicidal behaviour, self-harm, emotional and internalising problems, body image, loneliness, sleep difficulties, post-traumatic stress and eating problems), externalising problems (externalising problems and conduct problems), general mental health and well-being (general mental health, subjective well-being and stress) and bullying (traditional, cyber and sexual bullying). If a study addressed multiple issues within these domains, we assigned a count to each specific issue it covered. For instance, if a study examined both anxiety and bullying, we recorded one count for anxiety and one for bullying.

From 1995 to 2024 covered in our review, internalising problems and bullying received the most attention in cross-national research. The number of studies focused on internalising problems increased over the years, with a peak around 2022 (n=20). Research on externalising problems remained limited over the years. There was a moderate and steady increase in studies on general mental health and well-being, especially after 2021. The number of cross-national studies on traditional bullying gradually increased after 2004, while cyberbullying emerged as a major research area around 2017.

The prevalence of most mental health and psychosocial issues was similar between LMICs and HICs, with no clear trend favouring either group. In some cases, findings were mixed; some studies reported a higher prevalence in HICs, while others found the opposite. One exception to this is traditional bullying; a majority of the studies reported a higher prevalence in LMICs (n=26), while two studies found the opposite. The most commonly used measures included mathematics anxiety questions used in the PISA survey,^22^ the Youth Self-Report,^23^ the Strengths and Difficulties Questionnaire^24^ and bullying questions used in the HBSC survey^25^ (see online supplemental table 4 for the summary of included studies and online supplemental tale 5 for the characteristics of all included studies).

## Discussion

This is the first systematic review that provides a comprehensive summary of cross-national studies on adolescent mental health problems. A key strength of this review is its wide geographical and population coverage, including more than 12 million adolescents in 166 countries around the world. The review examined a range of key mental health and psychosocial problems among adolescents, with a specific focus on research from LMICs, addressing a critical gap in global literature. Based on the structured narrative synthesis, this review identified three major patterns across the included studies. First, the most commonly used international surveys collecting data on adolescent mental health during the review period were the HBSC (19.8%), the GSHS (12.2%) and PISA (8.1%). Most studies were based on cross-sectional designs, using school-based questionnaires with self-reported measures. Second, 166 countries were included in cross-national research on adolescent mental health, while 52 countries were not included in any of the studies selected for this review. There has been an increasing number of LMICs included in cross-national research over the years, especially after 2020. Third, during the last 30 years, cross-national studies on adolescent mental health in LMICs have focused primarily on internalising problems and bullying, while research on externalising problems have been limited. While the prevalences of most mental health and psychosocial issues were comparative or mixed between LMICs and HICs, traditional bullying was higher in LMICs in the majority of studies. These synthesised findings highlight substantial disparities in geographic coverage, research focus and outcome trends, which are likely shaped by cultural, socioeconomic and structural contexts. They also point to persistent gaps in global adolescent mental health research, particularly regarding under-represented regions, externalising behaviours and measurement standardisation.

The most common international survey used to collect data on adolescent mental health was the HBSC (n=34), followed by the GSHS (n=21) and PISA (n=14). The HBSC study examines limited mental health-related variables: mental well-being measured using the WHO-5 Well-being Index, life satisfaction, loneliness, self-efficacy and health complaints across 51 countries in Europe and North America.^14^ The GSHS is the largest-scale survey available for adolescent mental health, and it involves over 100 countries, mainly LMICs.^15^ The study includes mental health-related measures such as suicide behaviour, mental health knowledge and bullying. PISA mainly focuses on academic achievement but contains a limited number of mental health-related measures: life satisfaction, sense of belonging at school and bullying.^16^ All of these surveys aim to collect data from a nationally representative sample of adolescents using a cluster sampling design. However, concerns have been raised about the representativeness of these surveys, noting that in some countries, particularly those with large populations, the samples only include students from specific regions or school types, rather than from the entire national student population.^26^,^30^

Most of the included studies were based on a cross-sectional design collecting data from adolescents at a single point in time.^31^ Cross-sectional studies are generally less time-consuming and relatively inexpensive to conduct.^32^ About 8% of the included studies had a longitudinal design, allowing researchers to provide insight into cause-and-effect relationships and to identify changes over time.^33^ School-based surveys were the most common type of survey. School-based surveys can reach a broad and diverse group of adolescents from different socioeconomic backgrounds in a controlled environment, saving time and resources. However, they may miss adolescents who are not enrolled in or not regularly attending school, such as dropouts or those with chronic absenteeism. This can lead to the under-representation of vulnerable groups, particularly in countries with low school attendance rates. Although our review did not directly compare school attendance across countries, it is important to note that attendance rates often vary by income level, with LMICs generally facing greater challenges in ensuring universal school access.^34^ As a result, the role of important socioeconomic factors such as marginalisation and poverty on adolescent mental health may remain underplayed. Most of the included studies relied solely on self-reported measures, which are standard practice in child and adolescent psychiatry research.^35^ However, it is important to note that the use of self-reported measures may introduce biases and measurement errors, impacting the reliability of results.^36^

Our review highlighted a significant lack of validated measures for assessing adolescent mental health in cross-national studies. Moreover, many of the commonly used measures were originally developed according to Western mental health constructs. The validity, reliability and universal applicability of measures are essential for comparing culturally diverse populations in different countries.^37 38^ More than 16% of the studies did not meet the criteria for cultural appropriateness and conceptual equivalence across countries, highlighting a significant challenge in cross-national research. The use of inadequately adapted instruments may misrepresent mental health symptoms or overlook culturally specific expressions of distress. Renwick and colleagues^9^ note that many studies conducted in LMICs use tools developed in HICs without proper equivalence assessment, which may not accurately capture the nuances of mental health conditions in diverse cultural contexts. This suggests that, rather than relying solely on tools developed in HICs, there is a critical need to support LMICs in creating and validating their own culturally relevant instruments, which can also serve as valuable resources for HICs seeking more inclusive and globally applicable assessments. Measurement validation processes typically involve determining the relevance of a concept in new contexts, addressing interpretation issues, assessing psychometric properties such as reliability and validity, and evaluating the scale’s overall performance.^38^ Our finding that only four papers conducted measurement invariance testing echoes a previous review on the evidence of measurement invariance testing for child and adolescent psychopathology measures, which showed that this critical assessment was rarely conducted.^39^ The review further highlights that only a minority of the reviewed scales showed evidence of suitability for cross-cultural comparative studies. Indeed, future efforts should focus on creating culturally adapted and well-validated mental health measures with flexible administration modes and open-access availability to promote cross-national research. Such development should also acknowledge different cultural conceptualisations of mental health, since Western perceptions and evaluations of psychopathology may not be applicable, as such, across cultures.^40^

A previous report showed that, on a global scale, during 2015 to 2019, research on child and adolescent mental health received disproportionately less grant funding than research focusing on the adult population.^41^ Our review, however, shows that cross-national research on adolescent mental health had remarkable global coverage, with studies included in this review representing 166 countries, including many LMICs. This highlights significant progress in understanding adolescent mental health across diverse settings. The number of publications increased, especially after 2020, and this could be attributed to the increased attention to mental health during and after the COVID-19 pandemic.^42^

However, gaps remain as 52 countries are not included in any of the studies. Most of these countries are LMICs (n=30), reflecting the financial and logistical challenges researchers may face in both conducting and publishing such studies in resource-limited settings.^43^ Global funding for mental health research is highly unequally distributed across geographic regions, with most funding awarded to researchers in HICs.^41^ In addition, the excluded countries were primarily from Sub-Saharan Africa (n=27). The region faces a combination of challenges, including socioeconomic instability, displacement due to conflict, limited mental health infrastructure and deep-seated stigma surrounding mental illness. These issues significantly shape the mental health experiences of adolescents, yet they remain understudied due to the lack of systematic data collection. This is concerning, since limiting research to, from a global perspective, mainly relatively homogeneous populations and contexts prevents a comprehensive understanding of global adolescent mental health trends and complexities.^44^ To address this, targeted efforts are needed to promote the inclusion of under-represented countries through means such as equitable funding mechanisms, capacity building and international collaborations focused on under-represented regions.^45^ Addressing these gaps will not only enhance the global understanding of adolescent mental health but also ensure equitable attention to the needs of under-represented regions like Sub-Saharan Africa.

In the past 30 years, various research topics have been examined in cross-national adolescent mental health research. Anxiety and depression have been extensively examined domains throughout the examined timeframe, reflecting their high prevalence in adolescents.^46^ Sleep problems, often comorbid with both anxiety and depression,^47^ received increased focus in 2022. This may be related to concerns of adolescent’s changing sleep routines due to the COVID-19 pandemic and excessive use of smartphones.^48 49^ Suicidal behaviour, including attempts, plans and ideation, was consistently assessed in the studies published in the last 20 years, likely due to its severe consequences, as suicide ranks globally as the third leading cause of death among adolescents.^50^ Traditional bullying was the most extensively examined subdomain, while studies on cyberbullying were relatively limited in comparison. Cyberbullying became a major research topic in cross-national research around the late 2010s along with technological advancements and availability. This aligns with findings from the bibliometric analysis, which highlighted a significant increase in cyberbullying research over the last two decades.^51^

In contrast, some domains remain under-researched, leaving gaps in our understanding of adolescent mental health. Externalising problems, such as conduct disorders and hyperactivity, lacked consistent cross-national research, particularly in LMICs. This may be due to several interplaying factors. Externalising problems overlap with other disciplines such as education, social sciences and criminology. These phenomena may therefore have been traditionally studied outside of the mental health field to a greater degree than internalising symptoms. Internalising symptoms may be more strongly perceived as associated with mental health, shifting focus away from externalising problems. Furthermore, while internalising symptoms are typically assessed with self-reports, externalising problems often require more laborious measures and, hence, more resources. Cross-national research on externalising problems would also warrant qualitative considerations as there is likely significant cultural variation in the norms, explanations, and perceptions of what constitutes disruptive behaviour in individual settings. These gaps highlight the need for greater attention directed to externalising problems, especially given their potential long-term impact on adolescent development.^52^

Some mental health themes showed inconsistent patterns of inclusion in cross-national adolescent research. Loneliness was frequently studied in the 2000s and again in 2017. However, there seemed to be lack of more recent cross-national studies on the theme, which is intriguing given the widespread concern about decreasing social interactions among adolescents and the negative association of loneliness with adolescent well-being.^53^ A similar pattern could be observed regarding the topic of body image, which was addressed in the 2000s and in recent years, but not 2012–2019. A contributing factor may be the limited tradition of body image research in LMIC. According to a bibliometric review,^54^ in latest decades, body image research has been predominantly conducted in certain Anglo-Saxon (HIC) countries. The review also showed that scholars from some LMICs (Eastern Europe and South America) were poorly connected to those established scholarly networks and that researchers from Africa did not appear at all. Another less consistently included theme was post-traumatic stress. It was only occasionally studied in the 2000s but has emerged as a more frequent theme in recent years. This may reflect the high number of children currently being exposed to armed conflicts globally^55^ as well as an increased awareness of the impact of adverse childhood experiences.^56^ Finally, it could be noted that, despite the increasing number of reports on young people’s psychological responses to global threats such as climate change, war and pandemic,^57^,^59^ subcategories for the mental health impact of these global threats (eg, climate anxiety) did not appear among the reviewed studies. Although it was outside of our age inclusion criteria, a study by Hickman *et al* investigated climate anxiety in young people aged 16–25 years across 10 countries, revealing that 59% were very or extremely worried about climate change.^60^ This underscores the importance of further research into the mental health impacts of global threats on young populations.

When studies compared the level of mental health and psychosocial problems between LMICs and HICs, most of them reported mixed or comparable findings. These findings indicate that mental health problems are a global concern, affecting adolescents across all income levels. Moreover, this result suggests that there are more complex interplays between individuals, their community and their environment on mental health issues in different countries, beyond a country’s income level. However, our review found variation by income level for some mental health problems. The prevalence of research on traditional bullying was consistently higher in LMICs than HICs. These patterns are difficult to interpret given that several factors contribute to this variation, including socioeconomic inequalities, educational challenges and differing cultural perceptions of aggressive behaviours.^61 62^ However, these observations are based on narrative synthesis rather than formal statistical comparison, and no subgroup analyses were conducted. Therefore, the findings should be interpreted with caution and should not be considered as definitive prevalence differences.

### Limitations

Our systematic review has several limitations. First, we restricted our review to published peer-reviewed articles in English, and six electronic databases were used for the search. This may have led to the systematic exclusion of findings published in non-English journals, which is a significant limitation for cross-national research. Second, substantial heterogeneity in the methodology and inconsistencies in the definition and measurement of key constructs made direct comparisons across studies challenging, which affected our ability to conduct meta-analyses. Narrative syntheses of studies are often criticised for their lack of transparency and potential for bias, in comparison with meta-analyses, which provide more statistically robust findings. Nonetheless, the use of a narrative synthesis was considered appropriate to address our research questions, which aimed to identify the existing international surveys, countries yet to be explored and key trends across countries in cross-national research on adolescent mental health and psychosocial problems. Third, there was a clear lack of validated measures in various languages to assess adolescent mental health, and the inclusion of studies using unvalidated measures could make cross-national comparisons misleading. In addition, several other methodological issues in the included studies may affect the robustness of the findings, such as low or unreported response rates, the use of convenience or non-representative samples and limited reporting on the reliability and validity of assessment tools. These factors may reduce comparability across countries and were taken into account during the interpretation of findings. Fourth, our review focused on studies comparing countries. This limited our ability to examine specific populations within individual countries, such as immigrant, refugee and indigenous adolescents. In addition, by excluding single-country studies, we may have overlooked high-quality national data, especially from countries not included in international surveys. However, our focus on cross-national studies allowed us to identify countries that are under-represented in comparative research across borders, which may reflect broader structural challenges such as limited research funding, inadequate infrastructure and imbalanced resource distribution. Fifth, our review excluded studies that focused solely on comparisons between HICs, which may have underestimated the overall research activity in HICs and potentially skewed the picture of global research disparities. However, we still found that many of the included cross-national studies were predominantly based in HICs. This suggests that the disparity in research on adolescent mental health may be even greater than reported and should be interpreted with this limitation in mind. In addition, 51 potentially suitable studies were omitted due to difficulties in accessing their full texts. This may have influenced the findings by limiting the representativeness of the evidence. Finally, the countries were categorised into HICs and LMICs using World Bank income classification categories in our review. While the World Bank’s classification offers a useful starting point for global comparisons, it might have led to oversights in recognising the diversity within categories. Mental health problems are influenced by social, cultural and political factors, such as health infrastructure, stigma and societal expectations, which may not correlate directly with income levels.

### Implications

Future research should prioritise comprehensive, methodologically sound cross-national studies that include LMICs. Addressing gaps in under-researched domains, such as externalising problems, while focusing on the inclusion of under-represented regions is essential for addressing global disparities and inform effective interventions. Future cross-national research should use validated instruments and multiple informant sources to measure the mental health of adolescents and include representative samples that reflect the diversity of the adolescent population. Particular attention should be given to including minorities, such as adolescents from immigrant backgrounds, those with disabilities and indigenous groups. Integrating research findings with existing knowledge on social inequalities and policy frameworks is crucial for understanding how these factors relate to adolescent well-being in different contexts.

## Supplementary material

10.1136/bmjgh-2025-019267 online supplemental file 1

## Data Availability

All data relevant to the study are included in the article or uploaded as supplementary information.
