# Supplementary material for: Cross-national research on adolescent mental health: a systematic review comparing research in low, middle and high-income countries
Source: BMJ Glob Health. 2025 Jul 25;10(7):e019267. doi: 10.1136/bmjgh-2025-019267 (PMC12306219; doi:10.1136/bmjgh-2025-019267)
Supplement: online supplemental file 1 [file bmjgh-10-7-s001.docx]

**Supplementary Appendix**

**Table S1**. Syntax used for the six database searches

**Web of Science**

| **Themes** | **Search string** |
| --- | --- |
| **Mental health** | ((TS=("Mental Health” OR  “Psychopathology” OR   "Emotional problem*" OR  “Emotional dysregulation” OR “Mood disorder*” OR  Depress* OR  Anxi* OR  stress* OR  “affective disorder*” OR  “Behaviour problem*” OR “Behavior problem*” OR “Behavioral problem*” OR “Behavioural problem*” OR   "Conduct problem*" OR  “disruptive behavio*” OR  Internali* OR Externali* OR   Suicid* OR non-suicid* OR selfharm OR self-harm* OR selfinjur* OR self-injur* OR   “Body image” OR   “Eating problem*” OR “Eating distress” OR “Eating behavior” OR “Eating behaviour” OR  “Eating disorder*” OR  "Attention Deficit” OR Hyperactiv* OR “ADHD” OR “Autism” OR “ASD” OR  "Psychological Trauma" OR “PTSD” OR lonel* OR “Social Isolation” OR “peer problem*” OR   bully* OR bullied OR cyberbullying OR    “sleep problem*” OR “sleeping problem*”)) AND |
| **Adolescent** | TS=(“School age*” OR   Adoles* OR   Teen* OR   Boy OR Boys OR Girl* OR   Minors* OR   Pubert* OR   Pubescen* OR   Prepubescen*  OR   “young people” OR “young person” OR   preteen OR   youth* OR   student* OR   “Secondary school” OR   “High school*” OR   Highschool* OR   “Junior high school” OR   “Middle school”)) AND |
| **Cross-cultural/national** | TS=("cross cultur*” OR cross-cultur* OR   “cross-national” OR “cross national” OR   multinational* OR multi-cultur* OR  transcultura* OR intercultura*) |
| **Total results** | **4207** |

**CINAHL/PsycInfo/PsycArticles**

| **Themes** | **Search string** |
| --- | --- |
| **Mental health** | (TI "Mental Health" OR TI "Psychopathology" OR TI "Emotional problem*" OR TI "Emotional dysregulation" OR TI "Mood disorder*" OR TI Depress* OR TI Anxi* OR TI stress* OR TI "affective disorder*" OR TI "Behaviour problem*" OR TI "Behavior problem*" OR TI "Behavioral problem*" OR TI "Behavioural problem*" OR TI "Conduct problem*" OR TI "disruptive behavio*" OR TI Internali* OR TI Externali* OR TI Suicid* OR TI non-suicid* OR TI selfharm OR TI self-harm* OR TI selfinjur* OR TI self-injur* OR TI "Body image" OR TI "Eating problem*" OR TI "Eating distress" OR TI "Eating behavior" OR TI "Eating behaviour" OR TI "Eating disorder*" OR TI "Attention Deficit" OR TI Hyperactiv* OR TI "ADHD" OR TI "Autism" OR TI "ASD" OR TI "Psychological Trauma" OR TI "PTSD" OR TI lonel* OR TI "Social Isolation" OR TI "peer problem*" OR TI bully* OR TI bullied OR TI cyberbullying OR TI "sleep problem*" OR TI "sleeping problem*" OR AB "Mental Health" OR AB "Psychopathology" OR AB "Emotional problem*" OR AB "Emotional dysregulation" OR AB "Mood disorder*" OR AB Depress* OR AB Anxi* OR AB stress* OR AB "affective disorder*" OR AB "Behaviour problem*" OR AB "Behavior problem*" OR AB "Behavioral problem*" OR AB "Behavioural problem*" OR AB "Conduct problem*" OR AB "disruptive behavio*" OR AB Internali* OR AB Externali* OR AB Suicid* OR AB non-suicid* OR AB selfharm OR AB self-harm* OR AB selfinjur* OR AB self-injur* OR AB "Body image" OR AB "Eating problem*" OR AB "Eating distress" OR AB "Eating behavior" OR AB "Eating behaviour" OR AB "Eating disorder*" OR AB "Attention Deficit" OR AB Hyperactiv* OR AB "ADHD" OR AB "Autism" OR AB "ASD" OR AB "Psychological Trauma" OR AB "PTSD" OR AB lonel* OR AB "Social Isolation" OR AB "peer problem*" OR AB bully* OR AB bullied OR AB cyberbullying OR AB "sleep problem*" OR AB "sleeping problem*") AND |
| **Adolescent** | (TI "School age*" OR TI Adoles* OR TI Teen* OR TI Boy OR TI Boys OR TI Girl* OR TI Minors* OR TI Pubert* OR TI Pubescen* OR TI Prepubescen* OR TI "young people" OR TI "young person" OR TI preteen OR TI youth* OR TI student* OR TI "Secondary school" OR TI "High school*" OR TI Highschool* OR TI "Junior high school" OR TI "Middle school" OR AB "School age*" OR AB Adoles* OR AB Teen* OR AB Boy OR AB Boys OR AB Girl* OR AB Minors* OR AB Pubert* OR AB Pubescen* OR AB Prepubescen* OR AB "young people" OR AB "young person" OR AB preteen OR AB youth* OR AB student* OR AB "Secondary school" OR AB "High school*" OR AB Highschool* OR AB "Junior high school" OR AB "Middle school") AND |
| **Cross-cultural/national** | (TI "cross cultur*" OR TI cross-cultur* OR TI "cross-national" OR TI "cross national" OR TI multinational* OR TI multi-cultur* OR TI transcultura* OR TI intercultura* OR AB "cross cultur*" OR AB cross-cultur* OR AB "cross-national" OR AB "cross national" OR AB multinational* OR AB multi-cultur* OR AB transcultura* OR AB intercultura*) |
| **Total results** | **3131** |

**Embase**

| **Themes** | **Search string** |
| --- | --- |
| **Mental health** | ('mental health':ab OR 'psychopathology':ab OR 'emotional problem*':ab OR 'emotional dysregulation':ab OR 'mood disorder*':ab OR depress*:ab OR anxi*:ab OR stress*:ab OR 'affective disorder*':ab OR 'behaviour problem*':ab OR 'behavior problem*':ab OR 'behavioral problem*':ab OR 'behavioural problem*':ab OR 'conduct problem*':ab OR 'disruptive behavio*':ab OR internali*:ab OR externali*:ab OR suicid*:ab OR 'non suicid*':ab OR selfharm:ab OR 'self harm*':ab OR selfinjur*:ab OR 'self injur*':ab OR 'body image':ab OR 'eating problem*':ab OR 'eating distress':ab OR 'eating behavior':ab OR 'eating behaviour':ab OR 'eating disorder*':ab OR 'attention deficit':ab OR hyperactiv*:ab OR 'adhd':ab OR 'autism':ab OR 'asd':ab OR 'psychological trauma':ab OR 'ptsd':ab OR lonel*:ab OR 'social isolation':ab OR 'peer problem*':ab OR bully*:ab OR bullied:ab OR cyberbullying:ab OR 'sleep problem*':ab OR 'sleeping problem*':ab OR 'mental health':ti OR 'psychopathology':ti OR 'emotional problem*':ti OR 'emotional dysregulation':ti OR 'mood disorder*':ti OR depress*:ti OR anxi*:ti OR stress*:ti OR 'affective disorder*':ti OR 'behaviour problem*':ti OR 'behavior problem*':ti OR 'behavioral problem*':ti OR 'behavioural problem*':ti OR 'conduct problem*':ti OR 'disruptive behavio*':ti OR internali*:ti OR externali*:ti OR suicid*:ti OR 'non suicid*':ti OR selfharm:ti OR 'self harm*':ti OR selfinjur*:ti OR 'self injur*':ti OR 'body image':ti OR 'eating problem*':ti OR 'eating distress':ti OR 'eating behavior':ti OR 'eating behaviour':ti OR 'eating disorder*':ti OR 'attention deficit':ti OR hyperactiv*:ti OR 'adhd':ti OR 'autism':ti OR 'asd':ti OR 'psychological trauma':ti OR 'ptsd':ti OR lonel*:ti OR 'social isolation':ti OR 'peer problem*':ti OR bully*:ti OR bullied:ti OR cyberbullying:ti OR 'sleep problem*':ti OR 'sleeping problem*':ti) AND |
| **Adolescent** | ('school age*':ab OR adoles*:ab OR teen*:ab OR boy:ab OR boys:ab OR girl*:ab OR minors*:ab OR pubert*:ab OR pubescen*:ab OR prepubescen*:ab OR 'young people':ab OR 'young person':ab OR preteen:ab OR youth*:ab OR student*:ab OR 'secondary school':ab OR 'high school*':ab OR highschool*:ab OR 'junior high school':ab OR 'middle school':ab OR 'school age*':ti OR adoles*:ti OR teen*:ti OR boy:ti OR boys:ti OR girl*:ti OR minors*:ti OR pubert*:ti OR pubescen*:ti OR prepubescen*:ti OR 'young people':ti OR 'young person':ti OR preteen:ti OR youth*:ti OR student*:ti OR 'secondary school':ti OR 'high school*':ti OR highschool*:ti OR 'junior high school':ti OR 'middle school':ti) AND |
| **Cross-cultural/national** | ('cross cultur*':ab OR 'cross-national':ab OR 'cross national':ab OR multinational*:ab OR 'multi cultur*':ab OR transcultura*:ab OR intercultura*:ab OR 'cross cultur*':ti OR 'cross-national':ti OR 'cross national':ti OR multinational*:ti OR 'multi cultur*':ti OR transcultura*:ti OR intercultura*:ti) |
| **Total results** | **1619** |

**PubMed**

| **Themes** | **Search string** |
| --- | --- |
| **Mental health** | (("Mental Health"[Title/Abstract] OR "Psychopathology"[Title/Abstract] OR "Emotional problem*"[Title/Abstract] OR "Emotional dysregulation"[Title/Abstract] O  R "Mood disorder*"[Title/Abstract] OR Depress*[Title/Abstract] OR Anxi*[Title/Abstract] OR stress*[Title/Abstract] OR "affective disorder*"[Title/Abstract] OR "Behaviour problem*"[Title/Abstract] OR "Behavior problem*"[Title/Abstract] OR "Behavioral problem*"[Title/Abstract] OR "Behavioural problem*"[Title/Abstract] OR "Conduct problem*"[Title/Abstract] OR "disruptive behavio*"[Title/Abstract] OR Internali*[Title/Abstract] OR Externali*[Title/Abstract] OR Suicid*[Title/Abstract] OR non-suicid*[Title/Abstract] OR selfharm[Title/Abstract] OR self-harm*[Title/Abstract] OR selfinjur*[Title/Abstract] OR self-injur*[Title/Abstract] OR "Body image"[Title/Abstract] OR "Eating problem*"[Title/Abstract] OR "Eating distress"[Title/Abstract] OR "Eating behavior"[Title/Abstract] OR "Eating behaviour"[Title/Abstract] OR "Eating disorder*"[Title/Abstract] OR "Attention Deficit"[Title/Abstract] OR Hyperactiv*[Title/Abstract] OR ADHD[Title/Abstract] OR ADD[Title/Abstract] OR Autis*[Title/Abstract] OR ASD[Title/Abstract] OR "Psychological Trauma"[Title/Abstract] OR PTSD[Title/Abstract] OR lonel*[Title/Abstract] OR "Social Isolation"[Title/Abstract] OR "peer problem*"[Title/Abstract] OR "sleep problem*"[Title/Abstract] OR "sleeping problem*"[Title/Abstract] OR bully*[Title/Abstract] OR bullied[Title/Abstract] OR cyberbullying[Title/Abstract]) AND |
| **Adolescent** | ("School age*"[Title/Abstract] OR Adoles*[Title/Abstract] OR Teen*[Title/Abstract] OR Boy[Title/Abstract] OR Boys[Title/Abstract] OR Girl*[Title/Abstract] OR Minors*[Title/Abstract] OR Pubert*[Title/Abstract] OR Pubescen*[Title/Abstract] OR Prepubescen*[Title/Abstract] OR "young people"[Title/Abstract] OR "young person"[Title/Abstract] OR preteen[Title/Abstract] OR youth*[Title/Abstract] OR student*[Title/Abstract] OR "Secondary school"[Title/Abstract] OR "High school*"[Title/Abstract] OR Highschool*[Title/Abstract] OR "Junior high school"[Title/Abstract] OR "Middle school"[Title/Abstract])) AND |
| **Cross-cultural/national** | ("cross cultur*"[Title/Abstract] OR cross-cultur*[Title/Abstract] OR "cross-national"[Title/Abstract] OR "cross national"[Title/Abstract] OR multinational*[Title/Abstract] OR multi-cultur*[Title/Abstract] OR transcultura*[Title/Abstract] OR intercultura*[Title/Abstract]) |
| **Total results** | **1565** |

**Table S2**. JBI critical appraisal

| Author, year | | 1 | 2 | 3 | 4 | 5 | 6 | 7 | 8 | 9 | a | b | c | d |
| --- | --- | --- | --- | --- | --- | --- | --- | --- | --- | --- | --- | --- | --- | --- |
| Percentage of “Unclear” & “No” | | 2,3 % | 27,3 % | 9,3 % | 15,1 % | 15,7 % | 17,4 % | 16,3 % | 1,7 % | 43,0 % | 18,0 % | 16,3 % | 24,4 % | 1,7 % |
| [1] | Abio 2022 | Yes | Yes | Yes | Yes | Yes | Yes | Yes | Yes | Yes | Yes | Yes | NA | Yes |
| [2] | AlSabbah 2009 | Yes | Yes | Yes | Yes | Yes | Yes | Yes | Yes | Unclear | Yes | Yes | Yes | Yes |
| [3] | Arat 2017 | Yes | Yes | Yes | Yes | Yes | Yes | Yes | Yes | Yes | Yes | Yes | Yes | Yes |
| [4] | Assarsson 2018 | Yes | Yes | Yes | Yes | Yes | Yes | Yes | Yes | Unclear | Yes | Yes | Yes | Yes |
| [5] | Athanasiou 2018 | Yes | Yes | Yes | Yes | Yes | Yes | Yes | Yes | Yes | Yes | Yes | Unclear | Yes |
| [6] | Auerbach 2010 | Yes | Unclear | Unclear | Yes | Unclear | Yes | Yes | Yes | Unclear | No | Unclear | Yes | Yes |
| [7] | Auerbach 2011 | Yes | Unclear | Unclear | Yes | Yes | Yes | Yes | Yes | Yes | Yes | Yes | Yes | Yes |
| [8] | Badura 2021 | Yes | Yes | Yes | Yes | Yes | Yes | Yes | Yes | Yes | Yes | Yes | Yes | Yes |
| [9] | Bagley 1999 | Yes | Yes | Unclear | Yes | Yes | Yes | Yes | Yes | Unclear | Yes | Unclear | Yes | Yes |
| [10] | Baird 2019 | Yes | Yes | Yes | Yes | Yes | Yes | Yes | Yes | Yes | Yes | Yes | Yes | Yes |
| [11] | Balogun 2014 | Yes | Yes | Yes | No | Yes | Yes | Yes | Yes | Yes | Yes | Yes | Yes | Yes |
| [12] | Beckwith 2022 | Yes | Yes | Yes | Yes | Yes | Yes | Yes | Yes | Yes | Yes | Yes | Yes | Yes |
| [13] | Biswas 2022 | Yes | Yes | Yes | Yes | Yes | Yes | Yes | Yes | NA | NA | NA | NA | Yes |
| [14] | Bochaver 2022 | Yes | Yes | Yes | Yes | Yes | Yes | Yes | Yes | Unclear | Yes | Yes | Yes | Yes |
| [15] | Bravo-Sanzana 2022 | Yes | Yes | Yes | Yes | Yes | Yes | Yes | Yes | Yes | Yes | Yes | Yes | Yes |
| [16] | Bravo-Sanzana 2023 | Yes | Yes | Yes | Yes | Yes | Yes | Yes | Yes | Unclear | Yes | Yes | Unclear | Yes |
| [17] | Brown 2008 | Yes | Yes | Yes | Yes | Yes | Yes | Yes | Yes | Yes | No | Yes | Yes | Yes |
| [18] | Buist 2017 | Yes | No | Yes | Yes | Unclear | Yes | Yes | Yes | Unclear | Yes | Yes | Yes | Yes |
| [19] | Calmaestra 2020 | Yes | Yes | Yes | Yes | Yes | Yes | Yes | Yes | Unclear | Yes | Yes | Unclear | Yes |
| [20] | Campbell 2021 | Yes | Yes | Yes | No | Yes | Yes | Yes | Yes | Yes | Yes | Yes | Yes | Yes |
| [21] | Chen 1998 | Yes | No | No | Yes | Unclear | No | Yes | Yes | Unclear | No | Yes | No | Yes |
| [22] | Chen 2004 | Yes | Yes | Yes | Yes | Unclear | Yes | Yes | No | Unclear | Yes | Yes | Yes | Yes |
| [23] | Chen 2020 | Yes | Yes | Yes | Yes | Yes | No | Yes | Yes | Yes | Yes | No | Yes | Yes |
| [24] | Chen 2020 | Yes | Yes | Yes | Yes | Unclear | Yes | Yes | Yes | Unclear | Yes | Yes | NA | Yes |
| [25] | Chen 2020 | Yes | Yes | No | Yes | Unclear | Yes | Unclear | Yes | Unclear | Yes | Yes | NA | Yes |
| [26] | Chen 2023 | Yes | No | Yes | Yes | Yes | Unclear | Yes | Yes | Unclear | Yes | Yes | Yes | Yes |
| [27] | Chester 2015 | Yes | Unclear | Unclear | No | Unclear | No | Yes | Yes | Unclear | Unclear | Unclear | Unclear | Yes |
| [28] | Chudal 2022 | Yes | Yes | Yes | Yes | Yes | Yes | Yes | Yes | Yes | Yes | Yes | Yes | Yes |
| [29] | Cosma 2020 | Yes | Yes | Unclear | Yes | Yes | No | No | Yes | Unclear | Unclear | Yes | Yes | Yes |
| [30] | Cosma 2022 | Yes | Yes | Yes | No | Yes | Yes | Yes | Yes | Yes | Yes | Yes | Yes | Yes |
| [31] | Cosma 2022 | Yes | Yes | Unclear | Yes | Yes | Yes | Yes | Yes | Yes | Yes | Unclear | Unclear | Yes |
| [32] | Craig 2009 | Yes | Yes | Yes | No | Yes | Yes | Unclear | Yes | Yes | Yes | Unclear | Unclear | Yes |
| [33] | Craig 2020 | Yes | Yes | Yes | Unclear | Yes | Yes | Unclear | Yes | Yes | Yes | Yes | Unclear | Yes |
| [34] | Crocetti 2015 | Yes | Yes | Yes | Yes | Yes | Yes | Unclear | Yes | Unclear | Yes | Yes | Unclear | Yes |
| [35] | Crous 2017 | Yes | Yes | Yes | Yes | Yes | Yes | Yes | Yes | Yes | Yes | Yes | Yes | Yes |
| [36] | Delvecchio 2015 | Yes | No | Yes | Yes | Yes | Yes | Yes | Yes | Unclear | Yes | Yes | Unclear | Yes |
| [37] | Deryol 2022 | Yes | Yes | Yes | No | Yes | Unclear | Unclear | Yes | Unclear | Yes | Unclear | Unclear | Yes |
| [38] | DiGiunta 2018 | Yes | Yes | Yes | Yes | Yes | Yes | Yes | Yes | Yes | Yes | Yes | Yes | Yes |
| [39] | DiGiunta 2020 | Yes | Yes | Yes | Yes | Yes | Yes | Unclear | Yes | Yes | Yes | Yes | Yes | Yes |
| [40] | DiGiunta 2023 | Yes | Yes | Yes | No | Yes | Yes | Yes | Yes | Unclear | Yes | Yes | Yes | Yes |
| [41] | Dmitrieva 2004 | Yes | Yes | Yes | No | Yes | Yes | Unclear | Yes | Yes | No | Yes | Yes | Yes |
| [42] | Doty 2023 | Yes | No | Yes | Yes | No | Yes | No | Yes | Unclear | No | No | No | Yes |
| [43] | Due 2005 | Yes | Yes | Yes | Yes | Yes | Yes | Unclear | Yes | Yes | Yes | Yes | Unclear | Yes |
| [44] | Due 2008 | Yes | Yes | Yes | Yes | Yes | Yes | Yes | Yes | Yes | Yes | Yes | Yes | Yes |
| [45] | Due 2009 | Yes | Yes | Yes | Yes | Yes | Yes | Unclear | Yes | Yes | Yes | No | Yes | Yes |
| [46] | Duinhof 2020 | Yes | Yes | Yes | Yes | Yes | Yes | Yes | Yes | Unclear | Yes | Yes | Yes | Yes |
| [47] | Dzielska 2020 | Yes | Yes | Yes | Yes | Yes | Unclear | Yes | Yes | Yes | Yes | Unclear | Unclear | Yes |
| [48] | Elgar 2009 | Yes | Yes | Yes | Yes | Yes | Yes | Yes | Yes | Yes | Yes | Yes | Yes | Yes |
| [49] | Elgar 2015 | Yes | Yes | Yes | Yes | Yes | Yes | Yes | Yes | Yes | Yes | Yes | Yes | Yes |
| [50] | Erskine 2024 | Yes | No | Yes | No | Yes | Yes | Yes | Yes | Yes | No | Yes | Yes | Yes |
| [51] | Eskin 1995 | Yes | No | Yes | Yes | Unclear | No | Yes | Yes | Unclear | No | No | No | Yes |
| [52] | Eskin 2014 | Yes | No | Yes | Yes | Unclear | No | No | Yes | Unclear | Yes | Yes | Yes | Yes |
| [53] | Eslea 2004 | Yes | Yes | Yes | Yes | Yes | Yes | Yes | Yes | Yes | Yes | Yes | Yes | Yes |
| [54] | Farruggia 2004 | Yes | No | Yes | No | Yes | No | No | Yes | No | No | No | No | Yes |
| [55] | Fine 2022 | Yes | Unclear | Yes | Yes | Yes | Yes | Yes | Yes | Unclear | Yes | Unclear | Unclear | Yes |
| [56] | Fine 2023 | Yes | Yes | Yes | No | Yes | Yes | Yes | Yes | Unclear | No | Yes | Yes | Yes |
| [57] | Fismen 2022 | Yes | Yes | Yes | Yes | Yes | Yes | Yes | Yes | Yes | Yes | Yes | Yes | Yes |
| [58] | Fleming 2010 | Yes | Yes | Yes | Yes | Yes | Yes | Yes | Yes | Yes | Yes | Yes | Yes | Yes |
| [59] | Frenzel 2007 | Yes | No | Yes | Yes | No | No | No | Yes | Unclear | No | Yes | Yes | Yes |
| [60] | Germani 2021 | Yes | No | Yes | Yes | Yes | Unclear | Yes | Yes | Unclear | Yes | Unclear | No | Yes |
| [61] | Ghekiere 2019 | Yes | Yes | Yes | Yes | Yes | Yes | Yes | Yes | Unclear | Yes | Yes | Yes | Yes |
| [62] | Gillé 2021 | Yes | Yes | Yes | Yes | Yes | Yes | Yes | Yes | Yes | Yes | Yes | Yes | Yes |
| [63] | Gobina 2008 | Yes | Yes | Yes | Yes | Yes | No | No | Yes | Yes | Yes | Unclear | Unclear | Yes |
| [64] | Gomez-Baya 2022 | Yes | Yes | Yes | Yes | Yes | Yes | Yes | Yes | Yes | Yes | Yes | Yes | Yes |
| [65] | Görzig 2017 | Yes | Yes | Yes | Yes | Yes | Yes | Yes | Yes | Yes | Yes | Yes | Yes | Yes |
| [66] | Govorova 2020 | Yes | Yes | Yes | Yes | Yes | Yes | Yes | Yes | Yes | Yes | Yes | Yes | Yes |
| [67] | Greenberger 2000 | Yes | No | Yes | Yes | Yes | Yes | Yes | Yes | Unclear | Yes | Yes | Yes | Unclear |
| [68] | Greenberger 2000 | Yes | Yes | No | Yes | Yes | Yes | Yes | Yes | Yes | Yes | Yes | Yes | Yes |
| [69] | Gross-Manos 2022 | Yes | Yes | Yes | Yes | Yes | Yes | Yes | Yes | No | Yes | Yes | Unclear | Yes |
| [70] | Güngör 2010 | Yes | No | Yes | Yes | Yes | No | No | Yes | Yes | Yes | Yes | Yes | Yes |
| [71] | Gupta 2013 | Yes | No | Yes | Yes | Yes | No | No | Yes | Yes | Yes | No | Yes | Yes |
| [72] | Haid 2010 | Yes | No | Yes | Yes | Yes | Yes | Yes | Yes | Unclear | No | Yes | Yes | Yes |
| [73] | Han 2021 | Yes | Yes | Yes | Yes | Yes | Yes | Yes | Yes | Yes | Yes | Yes | Yes | Yes |
| [74] | Harel-Fisch 2010 | Yes | Yes | Yes | Yes | Yes | Yes | Yes | Yes | Yes | Yes | Yes | Yes | Yes |
| [75] | Harel-Fisch 2011 | Yes | Yes | Yes | Yes | Yes | Yes | Yes | Yes | Yes | Yes | Yes | Yes | Yes |
| [76] | Harel-Fisch 2012 | Yes | Yes | Yes | Yes | Yes | No | Unclear | Yes | Unclear | Yes | Yes | Yes | Yes |
| [77] | Heinz 2020 | Yes | Yes | Yes | Yes | Yes | Yes | Yes | Yes | Unclear | Yes | Yes | Yes | Yes |
| [78] | Hillekens 2020 | Yes | Yes | Yes | Unclear | Yes | Yes | Yes | Yes | Yes | Yes | Yes | Unclear | Yes |
| [79] | Högberg 2021 | Yes | Yes | Yes | Yes | Yes | Yes | Yes | Yes | Unclear | Yes | Yes | Yes | Yes |
| [80] | Hosozawa 2021 | Yes | Yes | Yes | Yes | Yes | Unclear | Yes | Yes | Yes | Yes | Yes | Yes | Yes |
| [81] | Hussein 2010 | Yes | Yes | Yes | Unclear | Yes | Yes | Yes | Yes | Unclear | Yes | Yes | Yes | Yes |
| [82] | Imran 2021 | Yes | Yes | Yes | Yes | Yes | Yes | Yes | Yes | Yes | Yes | Yes | Yes | Yes |
| [83] | Isaksson 2023 | Yes | Yes | Yes | Yes | Yes | Yes | Yes | Yes | Yes | Yes | Yes | Yes | Yes |
| [84] | Iwawaki 2001 | Yes | No | Yes | No | Yes | Unclear | No | Yes | Unclear | No | No | No | Yes |
| [85] | Jacob 2020 | Yes | Yes | Yes | Yes | Yes | Yes | Yes | Yes | Yes | Yes | Yes | Yes | Yes |
| [86] | Jacob 2020 | Yes | Yes | Yes | Yes | Yes | Yes | Yes | Yes | Yes | Yes | Yes | Yes | Yes |
| [87] | Jessor 2003 | Yes | No | Yes | Yes | Unclear | Unclear | Unclear | Yes | Unclear | Yes | Yes | Yes | Yes |
| [88] | Johnson 2024 | Yes | Yes | Yes | Yes | Yes | Yes | Yes | Yes | Yes | Yes | Yes | Yes | Yes |
| [89] | Kahumoku 2011 | Yes | Yes | Yes | Yes | Yes | No | No | Yes | Unclear | Yes | No | Unclear | Yes |
| [90] | Kakar 2023 | Yes | Yes | Yes | Yes | Yes | Yes | Yes | Yes | Unclear | Yes | Yes | Yes | Yes |
| [91] | Kakar 2023 | Yes | Unclear | Unclear | Yes | Unclear | Yes | Yes | Yes | Unclear | Yes | Yes | Yes | Yes |
| [92] | Kalaycıoğlu 2015 | Yes | Yes | Yes | Yes | Yes | Yes | Yes | Yes | Yes | Yes | Yes | Yes | Yes |
| [93] | Kapetanovic 2020 | Yes | Yes | Yes | Yes | Yes | Yes | Yes | Yes | Yes | Yes | Yes | Yes | Yes |
| [94] | Katsantonis 2021 | Yes | Yes | Yes | Yes | Yes | Yes | Yes | Yes | Yes | Yes | Yes | Unclear | Yes |
| [95] | Kayano 2008 | Yes | Unclear | Yes | No | Unclear | Yes | Yes | Yes | Unclear | No | Unclear | Unclear | Yes |
| [96] | Kearns 2022 | Yes | Yes | Yes | Yes | Yes | Yes | Yes | Yes | Unclear | Yes | Yes | Yes | Yes |
| [97] | Khan 2021 | Yes | Yes | Yes | Yes | Yes | Yes | Yes | Yes | Yes | Yes | Yes | Yes | Yes |
| [98] | Khan 2022 | Yes | Yes | Yes | Yes | Yes | Yes | Yes | Yes | Yes | Yes | Yes | Yes | Yes |
| [99] | Kim 2021 | Yes | No | Yes | Yes | Yes | No | No | Yes | Unclear | No | No | Yes | Yes |
| [100] | Kim 2022 | Yes | Yes | Yes | Yes | Yes | Yes | Yes | Yes | Unclear | Yes | Yes | Yes | Yes |
| [101] | King 2024 | Yes | Yes | Yes | No | Yes | Yes | Unclear | Yes | Unclear | Yes | Yes | Yes | Yes |
| [102] | Klinger 2015 | Yes | Yes | Yes | Yes | Yes | Yes | Yes | Yes | Yes | Yes | Yes | Yes | Yes |
| [103] | Koenig 2021 | Yes | Yes | Yes | Yes | Yes | Yes | Yes | Yes | Unclear | Yes | Yes | Yes | Yes |
| [104] | Kokkevi 2012 | Yes | Yes | Yes | Yes | Yes | Yes | Yes | Yes | Yes | Yes | Yes | Yes | Yes |
| [105] | Kokkevi 2012 | Yes | Yes | Yes | Yes | Yes | Yes | Yes | Yes | Yes | Yes | Yes | Yes | Yes |
| [106] | Koyanagi 2019 | Yes | Yes | Yes | Yes | Yes | Yes | Yes | Yes | Yes | Yes | Yes | Yes | Yes |
| [107] | Lai 2008 | Yes | Unclear | Yes | Unclear | Unclear | Yes | Yes | Yes | Unclear | Yes | Yes | Unclear | Yes |
| [108] | Lambert 1998 | Yes | Unclear | Yes | Yes | No | Yes | Yes | Yes | No | No | Yes | Yes | No |
| [109] | Law 2022 | Yes | No | Yes | Yes | Unclear | No | No | Yes | Unclear | No | No | Yes | Yes |
| [110] | Lee 2009 | Yes | Yes | Yes | Yes | Yes | Yes | Yes | Yes | Unclear | Yes | Yes | Unclear | Yes |
| [111] | Lewis 2017 | Yes | Yes | Yes | Yes | Yes | Yes | Yes | Yes | Yes | Yes | Yes | Yes | Yes |
| [112] | Li 2008 | Yes | Yes | Unclear | Yes | Yes | Yes | Yes | Yes | Yes | Yes | Yes | Yes | Yes |
| [113] | Li 2008 | No | No | Yes | Yes | No | No | Yes | Yes | Unclear | No | No | No | Yes |
| [114] | Li 2015 | Yes | Yes | Yes | No | Yes | Yes | Yes | Yes | Unclear | Yes | Yes | Yes | Yes |
| [115] | Li 2016 | Yes | No | Yes | Yes | Unclear | Yes | Yes | Yes | Unclear | Yes | Yes | Unclear | Yes |
| [116] | Li 2019 | Yes | Yes | Yes | Yes | Unclear | Yes | Yes | Yes | Unclear | Yes | Unclear | Unclear | Yes |
| [117] | Liu 2018 | Yes | Yes | Yes | Yes | Yes | Yes | Yes | Yes | Yes | Yes | Yes | Yes | Yes |
| [118] | Liu 2020 | Yes | Yes | Yes | Yes | Yes | Yes | Yes | Yes | Yes | Yes | Yes | Yes | Yes |
| [119] | Lukoševičiūtė 2022 | Yes | Yes | Yes | Yes | Yes | Yes | Yes | No | Unclear | Yes | Yes | Unclear | No |
| [120] | Malykh 2013 | Unclear | Unclear | Yes | No | Unclear | Yes | Yes | Yes | Unclear | Unclear | Yes | Yes | Yes |
| [121] | Mancinelli 2021 | Yes | Unclear | Unclear | Yes | Yes | Yes | Yes | Yes | Yes | Yes | Yes | Yes | Yes |
| [122] | Marksteiner 2020 | Yes | Yes | Yes | Yes | Yes | Yes | Yes | Yes | Yes | Yes | Yes | Yes | Yes |
| [123] | McCabe 2012 | Yes | Unclear | Yes | Yes | Unclear | Yes | Yes | Yes | Yes | Yes | Yes | Unclear | Yes |
| [124] | McKinnon 2016 | Yes | Yes | Yes | Yes | Yes | Yes | Yes | Yes | Yes | Yes | Yes | Yes | Yes |
| [125] | Medina 2012 | Yes | Yes | Yes | Yes | Yes | Yes | Yes | Yes | Yes | Yes | Yes | No | Yes |
| [126] | Nansel 2004 | Yes | Yes | Yes | Yes | Yes | Yes | Yes | Yes | Yes | Yes | Yes | Yes | Yes |
| [127] | Nguyen 2022 | Yes | Yes | Yes | Yes | Yes | Yes | Yes | Yes | Yes | Yes | Yes | Yes | Yes |
| [128] | Ojala 2007 | Yes | Yes | Yes | Yes | Yes | Yes | Yes | Yes | Yes | Yes | Yes | Yes | Yes |
| [129] | Page 2006 | Yes | No | Yes | No | Yes | Yes | Yes | Yes | Yes | No | Yes | Yes | Yes |
| [130] | Page 2010 | Yes | Yes | Yes | Yes | Yes | Yes | Yes | Yes | Yes | Yes | Yes | Yes | Yes |
| [131] | Page 2013 | Yes | Yes | Yes | Yes | Yes | Yes | Yes | Yes | Yes | Yes | Yes | Yes | Yes |
| [132] | Pat-Horenczyk 2009 | Yes | No | Yes | Yes | Yes | Yes | Yes | Yes | Unclear | No | Yes | Yes | Yes |
| [133] | Pfoertner 2014 | Yes | Yes | Yes | Yes | Yes | Yes | Yes | Yes | Yes | Yes | Yes | Yes | Yes |
| [134] | Pronk 2017 | Yes | No | Yes | Yes | Yes | Unclear | Yes | Yes | Unclear | No | Unclear | No | Yes |
| [135] | Rajmil 2014 | Yes | No | Yes | Yes | Yes | Yes | Yes | Yes | No | Yes | Yes | Yes | Yes |
| [136] | Ravens-Sieberer 2008 | Yes | Yes | Yes | Yes | Unclear | Yes | Yes | Yes | Yes | Yes | Yes | Yes | Yes |
| [137] | Rescorla 2007 | Yes | Unclear | Yes | No | Yes | Yes | No | Yes | Unclear | Yes | Yes | Yes | Yes |
| [138] | Rodríguez-Hidalgo 2020 | Yes | Yes | Yes | Yes | Yes | Yes | Yes | Yes | Unclear | Yes | Yes | Yes | Yes |
| [139] | Ruchkin 2005 | Yes | Yes | Yes | Yes | No | Yes | Yes | Yes | No | No | Yes | Yes | Yes |
| [140] | Ruchkin 2006 | Yes | Yes | Yes | Yes | Yes | Yes | Yes | Yes | Yes | Yes | Yes | Yes | Yes |
| [141] | Samara 2019 | Yes | Yes | Yes | Yes | Yes | NA | Yes | Yes | Unclear | Yes | Yes | Unclear | Yes |
| [142] | Sentenac 2013 | Yes | Yes | Yes | Yes | Yes | Yes | Yes | Yes | Yes | Yes | Yes | Yes | Yes |
| [143] | Shapka 2018 | Yes | No | Yes | Yes | Unclear | No | Yes | Yes | Unclear | Yes | No | No | Yes |
| [144] | Shukla 2022 | No | No | Yes | Yes | Yes | Yes | Yes | Yes | Yes | No | Yes | Yes | Yes |
| [145] | Sitnikova 2022 | Yes | Yes | Yes | Yes | Yes | Yes | Yes | Yes | Unclear | Yes | Yes | Unclear | Yes |
| [146] | Skinner 2022 | Yes | Yes | No | No | No | Yes | Yes | Yes | Unclear | Yes | Yes | Yes | Yes |
| [147] | Šmigelskas 2018 | Yes | Yes | Yes | Yes | Yes | Yes | Yes | Yes | Yes | Yes | Yes | Yes | Yes |
| [148] | Springer 2007 | Yes | Unclear | Yes | Yes | Yes | Unclear | Yes | Yes | Unclear | Yes | Yes | Yes | Yes |
| [149] | Stoet 2016 | Yes | Yes | Yes | Yes | Yes | Yes | Yes | Yes | Yes | Yes | Yes | Yes | Yes |
| [150] | Sujoldzić 2007 | Yes | Yes | Yes | Unclear | Yes | Yes | Yes | Yes | Unclear | Yes | Unclear | Unclear | Yes |
| [151] | Tahmouresi 2014 | Yes | Yes | No | Yes | Yes | Yes | Yes | Yes | Unclear | Yes | Yes | Unclear | Yes |
| [152] | Tang 2020 | Yes | Yes | Yes | Yes | Yes | Yes | Yes | Yes | Yes | Yes | Yes | Yes | Yes |
| [153] | Thorsén 2022 | Yes | Yes | Yes | Yes | Yes | No | Yes | Yes | Yes | No | Unclear | Unclear | Yes |
| [154] | Toro 2006 | Yes | No | Yes | Yes | Unclear | Yes | Yes | Yes | Unclear | Yes | Yes | NA | Yes |
| [155] | Turner-Moore 2021 | No | No | No | Yes | Yes | Yes | Yes | Yes | Unclear | Yes | Yes | Yes | Yes |
| [156] | Tuttle 2023 | Yes | Yes | Yes | Yes | Yes | Yes | Yes | Yes | Yes | Yes | Yes | Yes | Yes |
| [157] | Vancampfort 2018 | Yes | No | Yes | Yes | Yes | Yes | Yes | Yes | Unclear | No | Yes | Yes | Yes |
| [158] | Vazsonyi 2015 | Yes | Yes | Yes | Yes | Yes | Yes | Yes | Yes | Yes | Yes | Yes | Yes | Yes |
| [159] | Verhulst 2003 | Yes | Yes | Yes | No | Yes | Yes | Yes | Yes | Yes | Yes | Yes | Yes | Yes |
| [160] | Vermeiren 2002 | Yes | Yes | Yes | Yes | Yes | No | No | Yes | Yes | No | Yes | Yes | Yes |
| [161] | Vittetoe 2002 | Yes | Yes | Yes | Yes | Yes | Yes | No | No | Unclear | Yes | Yes | Yes | Yes |
| [162] | Volk 2021 | Yes | No | Yes | Yes | Yes | Yes | Yes | Yes | Yes | No | Yes | Yes | Yes |
| [163] | Vore 2016 | Yes | No | No | Yes | Yes | Unclear | Unclear | Yes | Yes | Yes | Yes | Yes | Yes |
| [164] | Wang 2020 | Yes | Yes | Yes | Yes | Yes | Yes | Yes | Yes | Yes | Yes | Yes | Yes | Yes |
| [165] | Weine 1995 | Yes | No | Yes | Yes | Yes | Yes | Yes | Yes | Yes | No | Yes | Yes | Yes |
| [166] | Weitkamp 2019 | Yes | No | Yes | Yes | Yes | Yes | Yes | Yes | Yes | Yes | Yes | Yes | Yes |
| [167] | Yuan 2023 | Yes | Yes | Yes | Yes | Yes | Yes | Yes | Yes | Yes | Yes | Yes | Yes | Yes |
| [168] | Zgambo 2014 | Yes | Yes | Yes | Yes | Yes | Yes | Yes | Yes | Yes | Yes | Yes | No | Yes |
| [169] | Zhang 2023 | Yes | Yes | Yes | Yes | Yes | Yes | Yes | Yes | Yes | Yes | Yes | Yes | Yes |
| [170] | Zhao 2019 | Yes | Yes | Yes | No | Yes | Unclear | Yes | Yes | Yes | Yes | Unclear | Yes | Yes |
| [171] | Zhou 2009 | Yes | Yes | Yes | Yes | Yes | Yes | Yes | Yes | Yes | No | Yes | Unclear | Yes |
| [172] | Zietz 2022 | Yes | Yes | Yes | Yes | Yes | Yes | Yes | Yes | Yes | Yes | Yes | Yes | Yes |

NA Not applicable

**JBI Critical Appraisal Checklist for Studies Reporting Prevalence Data**

1. Was the sample frame appropriate to address the target population?

This question relies upon knowledge of the broader characteristics of the population of interest and the geographical area. If the study is of women with breast cancer, knowledge of at least the characteristics, demographics and medical history is needed. The term “target population” should not be taken to infer every individual from everywhere or with similar disease or exposure characteristics. Instead, give consideration to specific population characteristics in the study, including age range, gender, morbidities, medications, and other potentially influential factors. For example, a sample frame may not be appropriate to address the target population if a certain group has been used (such as those working for one organisation, or one profession) and the results then inferred to the target population (i.e. working adults).  A sample frame may be appropriate when it includes almost all the members of the target population (i.e. a census, or a complete list of participants or complete registry data).

1. Were study participants recruited in an appropriate way?

Studies may report random sampling from a population, and the methods section should report how sampling was performed. Random probabilistic sampling from a defined subset of the population (sample frame) should be employed in most cases, however, random probabilistic sampling is not needed when everyone in the sampling frame will be included/ analysed.  For example, reporting on all the data from a good census is appropriate as a good census will identify everybody.  When using cluster sampling, such as a random sample of villages within a region, the methods need to be clearly stated as the precision of the final prevalence estimate incorporates the clustering effect. Convenience samples, such as a street survey or interviewing lots of people at a public gatherings are not considered to provide a representative sample of the base population.

1. Was the sample size adequate?

The larger the sample, the narrower will be the confidence interval around the prevalence estimate, making the results more precise. An adequate sample size is important to ensure good precision of the final estimate. Ideally we are looking for evidence that the authors conducted a sample size calculation to determine an adequate sample size.  This will estimate how many subjects are needed to produce a reliable estimate of the measure(s) of interest. For conditions with a low prevalence, a larger sample size is needed. Also consider sample sizes for subgroup (or characteristics) analyses, and whether these are appropriate. Sometimes, the study will be large enough (as in large national surveys) whereby a sample size calculation is not required. In these cases, sample size can be considered adequate.  When there is no sample size calculation and it is not a large national survey, the reviewers may consider conducting their own sample size analysis using the following formula: (Naing et al. 2006, Daniel 1999) n= Z2P(1-P) d2 Where: n= sample size Z = Z statistic for a level of confidence P = Expected prevalence or proportion (in proportion of one; if 20%, P = 0.2) d = precision (in proportion of one; if 5%, d=0.05)

1. Were the study subjects and setting described in detail?

Certain diseases or conditions vary in prevalence across different geographic regions and populations (e.g.  Women vs. Men, sociodemographic variables between countries).  The study sample should be described in sufficient detail so that other researchers can determine if it is comparable to the population of interest to them.

1. Was data analysis conducted with sufficient coverage of the identified sample?

Coverage bias can occur when not all subgroups of the identified sample respond at the same rate. For instance, you may have a very high response rate overall for your study, but the response rate for a certain subgroup (i.e. older adults) may be quite low.

1. Were valid methods used for the identification of the condition?

Here we are looking for measurement or classification bias.  Many health problems are not easily diagnosed or defined and some measures may not be capable of including or excluding appropriate levels or stages of the health problem. If the outcomes were assessed based on existing definitions or diagnostic criteria, then the answer to this question is likely to be yes. If the outcomes were assessed using observer reported, or self-reported scales, the risk of over- or under-reporting is increased, and objectivity is compromised. Importantly, determine if the measurement tools used were validated instruments as this has a significant impact on outcome assessment validity.

1. Was the condition measured in a standard, reliable way for all participants?

Considerable judgment is required to determine the presence of some health outcomes. Having established the validity of the outcome measurement instrument (see item 6 of this scale), it is important to establish how the measurement was conducted.  Were those involved in collecting data trained or educated in the use of the instrument/s? If there was more than one data collector, were they similar in terms of level of education, clinical or research experience, or level of responsibility in the piece of research being appraised? When there was more than one observer or collector, was there comparison of results from across the observers? Was the condition measured in the same way for all participants?

1. Was there appropriate statistical analysis?

Importantly, the numerator and denominator should be clearly reported, and percentages should be given with confidence intervals.  The methods section should be detailed enough for reviewers to identify the analytical technique used and how specific variables were measured. Additionally, it is also important to assess the appropriateness of the analytical strategy in terms of the assumptions associated with the approach as differing methods of analysis are based on differing assumptions about the data and how it will respond.

1. Was the response rate adequate, and if not, was the low response rate managed appropriately?

A large number of dropouts, refusals or “not founds” amongst selected subjects may diminish a study’s validity, as can a low response rates for survey studies. The authors should clearly discuss the response rate and any reasons for non-response and compare persons in the study to those not in the study, particularly with regards to their socio-demographic characteristics. If reasons for non-response appear to be unrelated to the outcome measured and the characteristics of non-responders are comparable to those who do respond in the study (addressed in question 5, coverage bias), the researchers may be able to justify a more modest response rate.

**Additional items for CROSS NATIONAL studies**

1. Was the research design comparable in terms of data collection, sample size, and participants in each country or region?

To make a valid cross-national comparison of survey results, sample characteristics and methods must be comparable across countries. Differences in study design, and data collection methods used in the individual countries can affect the comparability of results. If an incomparable research design, such as comparing samples with significantly different age groups, or employing different sampling methods (household or school-based surveys) or data collection methods were employed in the included countries, the study will be rated as low quality. However, studies do not necessarily have the exact same age group across countries if they are comparable age ranges.

1. Are the variables and measurements culturally appropriate and conceptually equivalent across countries?

The variables and measurement tools used in a study should be suitable and relevant within the context of a specific culture or country taking into account the cultural norms, values, beliefs, and practices of the population being studied. Researchers should respect the cultural context and the potential impact of their work on the populations they study especially when we examine sensitive topics such as gender norms or suicide behavior. At the same time, the constructs, ideas, and concepts being studied should have the same meaning and significance in diverse cultural settings. It is preferred to use the same validated and culturally adapted instrument across countries. If not, it is important to use conceptually equivalent instruments across countries.

1. Have translation and adaptation procedures been rigorously followed for survey instruments and questionnaires?

Ensuring that translation and adaptation procedures are rigorously followed for survey instruments and questionnaires is crucial for conducting high-quality, cross-cultural research. These procedures help maintain the integrity and validity of data, facilitating meaningful comparisons and insights across diverse populations and settings. Systematic steps such as back translation procedures should be conducted to ensure the linguistic and cultural validity of survey instruments and questionnaires.

1. Is the statistical or analytical approach appropriate for comparing data from different countries or regions?

When conducting cross-national research, it is crucial to employ an appropriate statistical or analytical approach to compare data from different countries or regions. If various units of measurement and reporting standards are used in different countries, were they standardized or was an appropriate approach employed to ensure comparability? For example, the cut-off points for the Strengths and Difficulties Questionnaire can vary between different countries and cultures. It is important to establish how to make the data comparable in terms of identifying, for example, a high-risk population. In a case of having different sample sizes or gender distribution across countries, were appropriate weighting approaches employed? When a difference in outcome variables was observed across countries, were any cultural, sociodemographic or any other appropriate factors explored to identify the possible explanations for such discrepancies?

**Table S3**. The number of studies by country/territory and income level

|  | LMIC | HIC | Total |
| --- | --- | --- | --- |
| Belgium | 0 | 70 | 70 |
| China | 61 | 0 | 61 |
| Poland | 22 | 39 | 61 |
| Germany | 0 | 56 | 56 |
| Italy | 0 | 56 | 56 |
| Greece | 0 | 55 | 55 |
| Netherlands | 0 | 52 | 52 |
| Spain | 0 | 52 | 52 |
| Russian Federation | 47 | 2 | 49 |
| Austria | 0 | 48 | 48 |
| France | 0 | 48 | 48 |
| Hungary | 20 | 28 | 48 |
| Sweden | 0 | 48 | 48 |
| Czech Republic | 19 | 27 | 46 |
| Finland | 0 | 46 | 46 |
| Latvia | 27 | 19 | 46 |
| Ireland | 0 | 45 | 45 |
| Portugal | 0 | 45 | 45 |
| Norway | 0 | 43 | 43 |
| Switzerland | 0 | 43 | 43 |
| Canada | 0 | 42 | 42 |
| Slovenia | 0 | 42 | 42 |
| Denmark | 0 | 41 | 41 |
| Estonia | 13 | 28 | 41 |
| Lithuania | 21 | 19 | 40 |
| Croatia | 19 | 19 | 38 |
| United Kingdom | 0 | 38 | 38 |
| Israel | 2 | 35 | 37 |
| United States | 0 | 35 | 35 |
| Indonesia | 34 | 0 | 34 |
| Congo, Rep. | 33 | 0 | 33 |
| Romania | 33 | 0 | 33 |
| Bulgaria | 30 | 1 | 31 |
| Slovak Republic | 10 | 21 | 31 |
| Türkiye | 31 | 0 | 31 |
| Iceland | 0 | 30 | 30 |
| Philippines | 28 | 0 | 28 |
| Thailand | 28 | 0 | 28 |
| Ukraine | 28 | 0 | 28 |
| Luxembourg | 0 | 27 | 27 |
| Malta | 2 | 24 | 26 |
| North Macedonia | 25 | 1 | 26 |
| Korea, Rep. | 0 | 21 | 21 |
| Argentina | 19 | 1 | 20 |
| Costa Rica | 19 | 1 | 20 |
| Morocco | 20 | 0 | 20 |
| Uruguay | 9 | 11 | 20 |
| Chile | 8 | 11 | 19 |
| Kenya | 19 | 0 | 19 |
| Peru | 19 | 0 | 19 |
| Japan | 0 | 18 | 18 |
| Jordan | 17 | 0 | 17 |
| Malaysia | 17 | 0 | 17 |
| Australia | 0 | 16 | 16 |
| Colombia | 16 | 0 | 16 |
| Greenland | 0 | 16 | 16 |
| Hong Kong SAR, China | 0 | 16 | 16 |
| Tanzania | 16 | 0 | 16 |
| United Arab Emirates | 1 | 15 | 16 |
| India | 15 | 0 | 15 |
| Mexico | 15 | 0 | 15 |
| Moldova | 12 | 3 | 15 |
| Namibia | 15 | 0 | 15 |
| Taiwan, China | 1 | 14 | 15 |
| Albania | 14 | 0 | 14 |
| Armenia | 14 | 0 | 14 |
| Brazil | 13 | 0 | 13 |
| Guyana | 13 | 0 | 13 |
| Tunisia | 13 | 0 | 13 |
| Uganda | 13 | 0 | 13 |
| Botswana | 12 | 0 | 12 |
| Pakistan | 12 | 0 | 12 |
| Serbia | 12 | 0 | 12 |
| Benin | 11 | 0 | 11 |
| Jamaica | 11 | 0 | 11 |
| Kazakhstan | 10 | 1 | 11 |
| Malawi | 11 | 0 | 11 |
| Seychelles | 7 | 4 | 11 |
| Trinidad and Tobago | 3 | 8 | 11 |
| Zambia | 11 | 0 | 11 |
| Ecuador | 10 | 0 | 10 |
| Georgia | 9 | 1 | 10 |
| Sri Lanka | 10 | 0 | 10 |
| Suriname | 10 | 0 | 10 |
| Venezuela, RB | 10 | 0 | 10 |
| El Salvador | 9 | 0 | 9 |
| Ghana | 9 | 0 | 9 |
| Guatemala | 9 | 0 | 9 |
| Honduras | 9 | 0 | 9 |
| Mauritania | 9 | 0 | 9 |
| New Zealand | 0 | 9 | 9 |
| Singapore | 0 | 9 | 9 |
| Zimbabwe | 9 | 0 | 9 |
| American Samoa | 8 | 0 | 8 |
| Antigua and Barbuda | 5 | 3 | 8 |
| Bolivia | 8 | 0 | 8 |
| Brunei Darussalam | 1 | 7 | 8 |
| Grenada | 8 | 0 | 8 |
| Kuwait | 2 | 6 | 8 |
| Mongolia | 8 | 0 | 8 |
| Myanmar | 8 | 0 | 8 |
| Samoa | 8 | 0 | 8 |
| St. Vincent and the Grenadines | 7 | 1 | 8 |
| Vietnam | 8 | 0 | 8 |
| Bahamas, The | 2 | 5 | 7 |
| Bangladesh | 7 | 0 | 7 |
| Belize | 7 | 0 | 7 |
| Dominica | 7 | 0 | 7 |
| Maldives | 7 | 0 | 7 |
| Nepal | 7 | 0 | 7 |
| Qatar | 0 | 7 | 7 |
| Solomon Islands | 7 | 0 | 7 |
| South Africa | 2 | 5 | 7 |
| St. Lucia | 6 | 1 | 7 |
| Vanuatu | 7 | 0 | 7 |
| Algeria | 6 | 0 | 6 |
| Azerbaijan | 6 | 0 | 6 |
| Cambodia | 6 | 0 | 6 |
| Dominican Republic | 6 | 0 | 6 |
| Egypt, Arab Rep. | 6 | 0 | 6 |
| Fiji | 6 | 0 | 6 |
| Iran, Islamic Rep. | 6 | 0 | 6 |
| Iraq | 6 | 0 | 6 |
| Montenegro | 6 | 0 | 6 |
| Oman | 4 | 2 | 6 |
| Tajikistan | 6 | 0 | 6 |
| Djibouti | 5 | 0 | 5 |
| Lao PDR | 5 | 0 | 5 |
| Macao SAR, China | 0 | 5 | 5 |
| Mozambique | 5 | 0 | 5 |
| Panama | 2 | 3 | 5 |
| Saudi Arabia | 0 | 5 | 5 |
| Afghanistan | 4 | 0 | 4 |
| Bosnia and Herzegovina | 4 | 0 | 4 |
| Ethiopia | 4 | 0 | 4 |
| Kosovo | 4 | 0 | 4 |
| St. Kitts and Nevis | 1 | 3 | 4 |
| Tonga | 4 | 0 | 4 |
| Yemen, Rep. | 4 | 0 | 4 |
| Bahrain | 1 | 2 | 3 |
| Barbados | 0 | 3 | 3 |
| Belarus | 3 | 0 | 3 |
| British Virgin Islands | 2 | 1 | 3 |
| Cayman Islands | 0 | 3 | 3 |
| Congo, Dem. Rep. | 3 | 0 | 3 |
| Kyrgyz Republic | 3 | 0 | 3 |
| Liechtenstein | 0 | 3 | 3 |
| Mauritius | 3 | 0 | 3 |
| Cyprus | 0 | 2 | 2 |
| French Polynesia | 0 | 2 | 2 |
| Isle of Man | 0 | 2 | 2 |
| Libya | 2 | 0 | 2 |
| Nauru | 2 | 0 | 2 |
| Nicaragua | 2 | 0 | 2 |
| Timor-Leste | 2 | 0 | 2 |
| West Bank and Gaza | 2 | 0 | 2 |
| Angola | 1 | 0 | 1 |
| Bhutan | 1 | 0 | 1 |
| Curaçao | 0 | 1 | 1 |
| Eswatini | 1 | 0 | 1 |
| Kiribati | 1 | 0 | 1 |
| Lebanon | 1 | 0 | 1 |
| Liberia | 1 | 0 | 1 |
| Paraguay | 1 | 0 | 1 |
| Puerto Rico | 0 | 1 | 1 |
| Syrian Arab Republic | 1 | 0 | 1 |
| Andorra | 0 | 0 | 0 |
| Aruba | 0 | 0 | 0 |
| Bermuda | 0 | 0 | 0 |
| Burkina Faso | 0 | 0 | 0 |
| Burundi | 0 | 0 | 0 |
| Cabo Verde | 0 | 0 | 0 |
| Cameroon | 0 | 0 | 0 |
| Central African Republic | 0 | 0 | 0 |
| Chad | 0 | 0 | 0 |
| Channel Islands | 0 | 0 | 0 |
| Comoros | 0 | 0 | 0 |
| Côte d'Ivoire | 0 | 0 | 0 |
| Cuba | 0 | 0 | 0 |
| Equatorial Guinea | 0 | 0 | 0 |
| Eritrea | 0 | 0 | 0 |
| Faeroe Islands | 0 | 0 | 0 |
| Gabon | 0 | 0 | 0 |
| Gambia, The | 0 | 0 | 0 |
| Gibraltar | 0 | 0 | 0 |
| Guam | 0 | 0 | 0 |
| Guinea | 0 | 0 | 0 |
| Guinea-Bissau | 0 | 0 | 0 |
| Haiti | 0 | 0 | 0 |
| Korea, Dem. Rep. | 0 | 0 | 0 |
| Lesotho | 0 | 0 | 0 |
| Madagascar | 0 | 0 | 0 |
| Mali | 0 | 0 | 0 |
| Marshall Islands | 0 | 0 | 0 |
| Micronesia, Fed. Sts. | 0 | 0 | 0 |
| Monaco | 0 | 0 | 0 |
| New Caledonia | 0 | 0 | 0 |
| Niger | 0 | 0 | 0 |
| Nigeria | 0 | 0 | 0 |
| Northern Mariana Islands | 0 | 0 | 0 |
| Palau | 0 | 0 | 0 |
| Papua New Guinea | 0 | 0 | 0 |
| Rwanda | 0 | 0 | 0 |
| San Marino | 0 | 0 | 0 |
| São Tomé and Príncipe | 0 | 0 | 0 |
| Senegal | 0 | 0 | 0 |
| Sierra Leone | 0 | 0 | 0 |
| Sint Maarten (Dutch part) | 0 | 0 | 0 |
| Somalia | 0 | 0 | 0 |
| South Sudan | 0 | 0 | 0 |
| St. Martin (French part) | 0 | 0 | 0 |
| Sudan | 0 | 0 | 0 |
| Togo | 0 | 0 | 0 |
| Turkmenistan | 0 | 0 | 0 |
| Turks and Caicos Islands | 0 | 0 | 0 |
| Tuvalu | 0 | 0 | 0 |
| Uzbekistan | 0 | 0 | 0 |
| Virgin Islands (U.S.) | 0 | 0 | 0 |

The list of above countries and territories is based on the World Bank classification and does not necessarily denote political independence.

HIC = High-income country; LMIC = Low- and/or middle-income country

**Table S4.** Summary of included studies: characteristics and key findings across domains

| **Domain** | **Sub-domain** | **N of articles** | **Total country/territory number (LMICs)** | **Most Commonly Used Measurements** | **Key findings (HICs vs LMICs)**  (*n* = number of studies) |
| --- | --- | --- | --- | --- | --- |
| Internalizing | Anxiety | 38 | 114 (67) | PISA Math Anxiety Scale, GSHS Anxiety Symptoms measure, HBSC Academic Stress measure, Social and Health Assessment (SAHA) | Majority of studies reported mixed findings (*n*=16), or comparable findings (*n*=6) between HICs and LMICs, while higher rates were in LMICs (*n*=4) or HICs (*n*=1) |
|  | Depression | 35 | 75 (55) | GSHS Depressive Symptoms measure, Children’s Depression Inventory (CDI), Center for Epidemiologic studies depression scale (CES-D), Global Early Adolescent Study (GEAS) depressive symptom index | Majority of studies reported comparable findings (*n*=11), or mixed findings (*n*=7) between HICs and LMICs, while higher rates were reported in LMICs (*n*=3) |
|  | Suicide behaviour and self-harm | 24 | 139 (106) | GSHS Suicide Behavior measure (suicidal ideation, plan, and attempt), HBSC Suicidal Ideation and Behavior measure, European School Survey Project on Alcohol and Other Drugs (ESPAD) Psychosocial Module (suicide ideation, plan, attempts, self-harm thoughts and self-harm) | Majority of studies reported mixed findings (*n*=10), between HICs and LMICs while similar findings (*n*=1) and higher rates were reported in LMICs (*n*=1) |
|  | Emotional and internalizing problems | 16 | 33 (20) | Youth Self-Report, Child Behavior Checklist (CBCL), Teacher's Report Form (TRF) | Mixed findings between HICs and LMICs were reported (*n*=6) while higher rates were reported in LMICs (*n*=6) |
|  | Body image | 10 | 69 (31) | HBSC Body Image measure | Higher rates were reported in HICs (*n*= 5) while three studies reported opposing findings (*n*=3) |
|  | Loneliness | 6 | 23 (16) | GSHS Loneliness measure | Mixed findings reported between HICs and LMICs (*n*=2) |
|  | Sleep difficulties | 6 | 132 (87) | GSHS Anxiety-induced Sleeplessness measure, HBSC Multiple Health Complaints scale | Higher rates were reported in HICs (*n*=2), while one study found mixed findings between HICs and LMICs |
|  | Post-traumatic stress | 4 | 8 (5) | Child posttraumatic Stress Reaction Index | Mixed findings reported between HICs and LMICs (*n*=3) |
|  | Eating problems | 3 | 6 (4) | Eating Attitude Test-26 (EAT-26), Eating Disorder Inventory-2 (EDI-2), Sociocultural Attitudes Towards Appearance Questionnaire-4 Revised (SATAQ-4R) | Higher rates were reported in HICs (*n*=2), while one study found mixed findings between HICs and LMICs (*n*=1) |
|  | Body image and eating distress | 1 | 2 (1) | EAT-26, Eating Disorders Assessment Questionnaire (CETCA), Questionnaire on Influences on Body Shape Model (CIMEC) | Higher rates were reported in HICs compared to LMICs |
| Externalizing | Externalizing difficulties | 14 | 62 (35) | Youth Self-Report (YSR), CBCL, TRF | Majority of studies reported mixed findings between HICs and LMICs (*n*=7), while higher rates were reported in LMICs (*n*=4) or HICs (*n*=1) |
|  | Conduct problems | 10 | 38 (24) | The Strengths and Difficulties Questionnaire (SDQ), Youth Risk Behaviour Surveillance System (YRBSS), Adolescent Health and Development Questionnaire (AHSQ), SAHA | Mixed findings reported between HICs and LMICs (*n*=4) while higher rates were reported in HICS (*n*=2) |
| General mental health and well-being | General mental health | 18 | 98 (50) | SDQ, HBSC symptom checklist, YSR, CBCL | Majority of studies reported mixed or comparable findings between HICs and LMICs (*n*=8) while higher rates were in HICs (*n*=6) or LMICs (*n*=2) |
|  | Subjective well-being | 8 | 53 (27) | Subjective Well-Being Scale (SWBS), Children’s World Subjective Well-being (CW-SWB), Psychological well-being (PWB), WHO-5 Well-being Index | Majority of studies reported mixed or comparable findings between HICs and LMICs (*n*=5) while higher rates were in LMICs (*n*=1) |
|  | Stress | 4 | 13 (7) | McMaster Family Assessment Device (FAD), Hassle Scale for Children (HSC), Problem Questionnaire (PQ) | Majority of studies reported mixed findings between HICs and LMICs (n=2) while higher rates were in HICs (n=1) |
| Bullying | Traditional bullying | 48 | 182 (60) | HBSC Bullying measure, Olweus Bullying scale, PISA Bullying measure, GSHS Bullying measure | Majority of studies reported higher rates in LMICs (*n*=26), while higher rates were HICs (*n*=2). The rest reported mixed findings. |
|  | Cyberbullying | 18 | 72 (31) | HBSC Bullying measure | Majority of studies reported higher rates in LMICs (*n*=7), while higher rates were HICs (*n*=4). The rest reported mixed findings. |
|  | Sexual bullying | 1 | 5 (1) | Sexual Bullying Questionnaire (SBQ) | Higher rates were reported in LMICs than HICs (*n*=1) |

HIC = High-income country; LMIC = Low- and middle-income country; HBSC = Health Behaviour in School-aged Children; GSHS = Global School-based Student Health Survey; PISA = Programme for International Student Assessment.

The number of countries/territories was extracted from the original study using the World Bank classification to categorize High-Income Countries (HICs) and Low- and Middle-Income Countries (LMICs) based on the data collection year.

**Table S5**. All included studies and characteristics

|  | Author, year | Total country/territory number (LMICs) | Measures: Scale names with reference or exact question used to measure. | Study name | Sample, age, informants | Key findings |
| --- | --- | --- | --- | --- | --- | --- |
| [1] | Abio 2022 | 53 (47) | Suicide ideation: During the past 12 months, did you ever seriously consider attempting suicide? Made a suicide plan: During the past 12 months, did you make a plan about how you would attempt suicide? Suicide attempt: During the past 12 months, how many times did you actually attempt suicide? Bullying victimisation: During the past 30 days, on how many days were you bullied? Loneliness: During the past 12 months, how often have you felt lonely? Anxiety: During the past 12 months, how often have you been so worried about something that you could not sleep at night? | GSHS | N = 193484, age = 12-16, self | The overall prevalence of suicide ideation, making a plan and suicide attempt were 10.4%, 10.3% and 11.0%, respectively. The highest prevalence rates reported were from the Americas. The strongest risk factors associated with suicidal behavior included anxiety, loneliness, no close friends and the substance abuse |
| [2] | AlSabbah 2009 | 24 (8) | Body weight dissatisfaction: At present are you on a diet or doing something else to lose weight? | HBSC | N= 103982 age= 11-15,  Self | The highest prevalence of body dissatisfaction was 34.1 in Ukraine as LMICs and 39.9 in Italy as HICs among boys and 61.8 in Czech Republic as LMICs and 56.8 in Slovenia as HICs among girls. Adolescents' age and overweight status were positively associated with body weight dissatisfaction in almost all countries. |
| [3] | Arat 2017 | 6 (6) | Loneliness: During the past 12 months, how often have you felt lonely?  Anxiety: During the past 12 months, how often have you been so worried about something that you could not sleep at night?  Depression: During the past 12 months, did you ever feel so sad or hopeless almost every day for 2 weeks or more in a row that you stopped doing your usual activities?  Suicidal ideation: During the past 12 months, did you ever seriously consider attempting suicide?  Suicide attempts: During the past 12 months, how many times did you actually attempt suicide? | GSHS | N = 23372, age = 11-17, self | In general, physical activity appears to facilitate positive mental health outcomes among adolescents. However, the ways in which different types of physical activity result in mental health differed to varying degrees across countries. High levels of exercising for 60 min per day within a week were significantly associated with a decrease in the likelihood of anxiety in the Philippines and China. Likewise, this study documented those high levels of exercising for 60 min per day within a week also linked to lower odds of loneliness in Pakistan, |
| [4] | Assarsson 2018 | 37 (34) | Suicidal ideation: During the past 12 months, did you ever seriously consider attempting suicide? | GSHS | N = 149306, age = 13-17, self | The rate of suicidal ideation ranged from 0.87% in Myanmar to 32.76% in Samoa. In 24 of 37 countries, girls were more likely to report suicide ideation than boys. The higher levels of national gender inequality were associated with higher levels of adolescent suicide ideation. |
| [5] | Athanasiou 2018 | 7 (1) | Cyberbullying victimization: Has someone acted in this kind of hurtful or nasty way to you in the past 12 months on the Internet? | N/A | N = 12372, age = 14-17, self | The highest rates of cyberbullying occurred in Romania (37.3%), Greece (26.8%) and Germany (24.3%), followed by Poland (21.5%). The lowest rates were found in the Netherlands (15.5%), Iceland (13.5%) and Spain (13.3%). In Romania, Poland and Germany cybervictimization was associated with a social network site use, whereas Internet use was associated with increased odds of cybervictimization only in Romania. |
| [6] | Auerbach 2010 | 2 (1) | Center for Epidemiologic Studies Depression Scale (CES-D; Radloff, 1977), Multidimensional Anxiety Scale for Children (MASC; March, 1997; Yao et al., 2007), Adolescent Life-Events Questionnaire (ALEQ; Hankin & Abramson, 2002), Responses to Stress Questionnaire - RSQ (Connor-Smith et al., 2000; Yao et al., 2010) | N/A | N = 547, age = 12-18, self | In both Canada and China, higher levels of coping deficits were associated with increases in depressive, but not anxious, symptoms following negative events. Gender differences did not emerge. |
| [7] | Auerbach 2011 | 2 (1) | Center of epidemiologic studies depression scale (CES-D; Radloff, 1977), Multidimensional anxiety scale for children - short form (MASC-SF; March, 1997), Adolescent life events questionnaire - revised (ALEQ; Hankin & Abramson, 2002), Aspirations index - revised (AI-R; e.g., Kasser & Ryan, 1996) | N/A | N = 660,  Age = 12-18, self | Higher levels of relative extrinsic aspirations predicted higher levels of depressive symptoms for Canadian and Chinese adolescents |
| [8] | Badura 2021 | 9 (5) | Four items from The HBSC symptom checklist measuring psychological complaints (feeling low, bad temper or irritability, feeling nervous and difficulties falling asleep) experienced in the last 6 months. | HBSC | N = 55429, age=11/13/15, Self | Psychological complains: The lowest mean score was in Russia while the highest mean score was in Poland. Mean was 5.27 (4.08). LMICs: 4.50 (4.08) Armenia, 5.72 (3.91) Czechia, 5.67 (4.38) Latvia, 4.69 (4.07) Moldova, 4.39 (4.19) Russia HICs: 5.00 (3.61) Belgium, 5.20 (4.12) Canada, 6.02 (4.29) Poland, 5.47 (3.75) Slovakia |
| [9] | Bagley 1999 | 6 (3) | Self-Completed Measure of Behaviourial and Emotional Problems (Sanford et al., 1992): emotinal disorders, somatic disorder, conduct disorder and hyperactivity General Functioning and Adolescent Conflict scales of the McMaster Family Assessment Device (Keitner et al., 1990): stress | Adolescent stress and adjustment | N = 2524, age=15-16, Self | Students in Canada, Britain, Hong Kong, and the Philippines report similar average stress levels and responses. In contrast, students in Pakistan and India report high averages for conduct and emotional disorders, as well as low self-esteem than other countries. Pakistani students experience the highest stress from school, family, and community, while Indian adolescents report the lowest. |
| [10] | Baird 2019 | 2 (2) | General Health Questionnaire - 12 (GHQ-12; Goldberg and Blackwell, 1970), Rosenberg's self-esteem scale (RSES; Rosenberg, 1965) | Gender and Adolescence: Global Evidence program | N=6489, age=10-12, Self | GHQ scores had a higher mean in Bangladesh compared to Ethiopia. The RSES scores were also slightly higher in Bangladesh than in Ethiopia.  A strong and large positive association between restrictive gender attitudes and the worse mental health in both Bangladesh and Ethiopia across gender and rural/urban location. On the other hand, restrictive gender norms were positively associated with worse mental health in Ethiopia, but a negative association was found in Bangladesh. |
| [11] | Balogun 2014 | 12 (12) | Depression: During the past 12 months, did you ever feel so  sad or hopeless almost every day for more than 2 weeks in a row that you stopped doing your usual activities?  Anxiety-induced sleeplessnes: During the past 12 months, how often have you been so worried about something that you could not sleep at night? | GSHS | N= 32001 age= 13-15  Self | Overall prevalence of depression varied across countries from 16.5% in Myanmar to 48.8% in Kenya. Overall prevalence of sleep problems varied across countries from 36% in Myanmar to 79.1% in Philippines. |
| [12] | Beckwith 2022 | 4 (3) | Bullying and violence perpetration: e.g., During the last six months, have you seen any of your FEMALE peers bully or threaten someone?  Depression: e.g., I feel sad | GEAS | N = 5762, age = 10-14, Self | Violence perpetration among young adolescents in high-poverty areas was linked to unsafe environments and risky peer behaviors, while strong parental ties, neighborhood cohesion, and egalitarian gender norms reduced risks. |
| [13] | Biswas 2022 | 40 (7) | Traditional bullying victimization; How often have you been bullied in school in the past couple of months?  In the past couple of months how often have you been cyberbullied? | HBSC | N = 214080, age = 11-15, self | A consistent finding is that traditional bullying victimization is considerably more common among adolescents across both LMICs and HICs than cyberbullying victimization. Positive family functioning, strong peer relationships, and greater school connectedness are associated with a lower risk of both forms of bullying victimization. |
| [14] | Bochaver 2022 | 2 (2) | Eight-item victimization scale from the Student Aggression and Victimisation Questionnaire (SAVQ) (Skrzypiec, 2015) | N/A | N = 1084, age = 12-17, self | Significant differences were detected on several items; one notable difference was the relationship to the perpetrator. In Mexico, the most common bully was siblings, while in Russia, the highest rank was for parents. |
| [15] | Bravo-Sanzana 2022 | 2 (2) | Well-Bing: Mental Health Continuum (Keyes, 2009) | Study by Global Research Alliance | N=3275, age=10-18, Self | languishing-moderate-flourishing Chile: Males: 9.7%, 40.8%, 49.5%, Females: 16.4%, 41.7%, 41.8%, Non-binary: 50.0%, 34.6%, 15.4% Mexico: Males: 8.9%, 38.0%, 53.1%, Females: 13.6%, 40.6%, 45.9%, Non-binary: 43.3%, 40.0%, 16.7% |
| [16] | Bravo-Sanzana 2023 | 7 (4) | The Student Aggression and Victimization Questionnaire (Skrzypiec, 2015), The Depression, Anxiety, and Stress Scale-21 Items (DASS-21; Lovibond, 1995), The Connor-Davidson Resilience Scale CD-RISC; Campbell-Sills, 2007) | Study by Global Research Alliance | N = 6423, age = 14.31, self | Adolescents in Chile and Russia reported the highest depression, anxiety, and stress, with victimization most prevalent in Russia and India. Resilience mediated the impact of victimization on mental health only in Chile, Indonesia, and Russia. |
| [17] | Brown 2008 | 8 (8) | Bullying: During the past 30 days, on how many days were you bullied?  Anxiety-related sleep loss: During the past 12 months, how often have you been so worried about something that you could not sleep at night?  Persistent loneliness: During the past 12 months, how often have you felt lonely?  Consider suicide: During the past 12 months, did you ever seriously consider attempting suicide?  Planned suicide: During the past 12 months, did you make a plan about how you would attempt suicide? | GSHS | N = 26510, age = 13-15, Self | Bullying behavior is common among boys and girls in eight countries and is associate with physical fighting and multiple adverse health risk behaviors. The prevalence of being bullied on least 1 day ranged from 25% in Tanzania to 63% in Zambia. About 16% of youth reported feelings of loneliness most of the time or always during the 12 months. The overall prevalence of sleep problem was 15%, and it was associated with being bullied. One in five of youth considered suicide and 22% of youth planned suicide during the 12 months preceding the survey. |
| [18] | Buist 2017 | 2 (1) | The Sibling Relationship Questionnaire (SRQ; Buhrmester  & Furman, 1990), Externalizing and internalizing problems: The Nijmegen Problem Behavior List (NPBL Research version; De Bruyn, Scholte, & Vermulst, 2005), Youth Self-Report (YSR; Achenbach, 1991; Verhulst, Van der Ende, & Koot, 1997) | N/A | N = 597, age Mean = 10.94 (SD 0.62), 10.81 (SD 0.80), self | Indian early adolescents reported higher levels of externalizing and internalizing problem behaviors compared to their Dutch counterparts. The influence of sibling and parent-child relationship quality on these behaviors is similar across both countries. |
| [19] | Calmaestra 2020 | 2 (1) | European Cyberbullying Intervention Project Questionnaire (ECIPQ; Ortega-Ruiz, 2016) | N/A | N= 33303, age=11-18, Self | One in four in Ecuador, and one in five in Spain, teenagers were involved in cyberbullying. Older students were more likely to involve in cyberbullying in both countries. |
| [20] | Campbell 2021 | 73 (31) | Psychological Distress: How often adolescents felt sad, miserable, scared, and afraid on a scale of never, rarely, sometimes and always.  Life satisfaction: How satisfied are you with life as a whole these days? Eudaemonic wellbeing: how much do you agree with these questions: My life has clear meaning or purpose. I have discovered a satisfactory meaning in life. I have a clear sense of what gives meaning to my life. | PISA | N=566829, age=11/13/15, Self | Adolescent girls generally experience poorer mental health than boys across various cultures. However, the extent of this gender gap varies significantly between countries, with some nations even showing a reversed trend. Higher GDP per capita correlates with both poorer average mental health and a wider gender gap. Countries with greater gender equality tend to exhibit larger gender disparities in mental health outcomes. |
| [21] | Chen 1998 | 3 (1) | Misconduct (20 items, e.g., got into a fist fight, broke or  damaged property on purpose) | N/A | N = 591, Mage = 13.8, self & Parent | The four groups of adolescents (European Americans, Chinese Americans, Taipei Chinese, Beijing, Chinese) reported similar levels and patterns of self-reported misconduct with no significant difference. |
| [22] | Chen 2004 | 4 (2) | Loneliness and social dissatisfaction (Asher, 1984).  Peer assessments of social behavior: The Revised Class Play (RCP;  Masten, 1985). | N/A | N = 2263, age = 9-12, self | Aggression made significant indirect contributions to the prediction of loneliness in Chinese children, but not in other samples. Shyness-sensitivity was associated with Loneliness directly in Brazilian and Italian children and indirectly through peer relationships in Canadian children but not associated with Loneliness in Chinese children. |
| [23] | Chen 2020 | 2 (1) | California School Climate and Safety Survey (CSCSS; e.g., Benbenishty & Astor, 2005) | N/A | N = 2582, grade = 7-9, Self | Verbal violence is most frequent. Male students are most likely to be aggressive and victimized. Grade-level differences in school violence were shown to be weak or insignificant. School nonattendance is generally associated with being kicked, punched, socially excluded, blackmailed, threatened, or sexually kissed without consent. The results were similar across countries. |
| [24] | Chen 2020 | 2 (1) | An adapted Chinese version of cyberbullying victimisation scale (Chen, 2018) | N/A | N = 2582, grade = 7-9, Self | Online curses, insults, and humiliation are the most prevalent forms of cyberbullying. Males are more likely to report perpetration in all countries, while no significant gender differences have been observed in victimization rates. Grade-level differences in cyberbullying were shown to be weak or insignificant across societies. |
| [25] | Chen 2020 | 2 (1) | An adapted Chinese version of cyberbullying victimisation scale (Chen, 2018), Brief Symptom Rating Scale (Chang et al., 2018; Chen & Wei, 2011; Chen,Wu, & Wei, 2020) | N/A | N = 1932, grade = 7-9, Self | Cybervictimisation was associated with psychological distress, and it had indirect association through parental support across countries and genders. The associations were stronger for Mainland Chinese students compared to Taiwanese ones. |
| [26] | Chen 2023 | 25 (10) | School bullying Victimization scale (e.g., Borualogo & Casas, 2021). Children's World Subjective Well-being (CW-SWB) Scale | ISCWEB | N=73182, age=10/12, Self | School victimization ranged 0.69 (1.25) in S Korea to 3.24 (2.43) in Namibia while subjective well-being ranged 46.01 (13.49) in Hong Kong to 57.59 (6.19) in Albania. Quality of family- child, peer, and teacher-child relationships mediate the association of material deprivation with school bullying victimization and subjective well-being in all countries. The family-child relationship is the strongest mediator between material deprivation and subjective well-being, while peer relationship is the strongest mediator between material deprivation and school victimization. |
| [27] | Chester 2015 | 33 (10) | Bullying victimization scale (Olweus, 1996) | HBSC | N= 581838, age=11/13/15, Self | Between 2001-02 and 2009-10, the percentage of children experiencing occasional bullying at school decreased from 33.5% to 29.2%, while chronic bullying declined from 12.7% to 11.3%. About one-third of the countries studied showed significant decrease in both occasional and chronic victimization for both genders. However, French-speaking Belgium reported significant increases in both types of bullying. |
| [28] | Chudal 2022 | 13 (7) | The Strengths and Difficulties Questionnaire (SDQ; Goodman, 1997) Traditional and Cyberbullying victimization: The students were asked  how often they had been bullied at school or outside school or cyberbullied in the past 6 months. | GCAMHS | N= 21688, age=13-15, Self | The study reported a 28.9% overall victimization rate, with 17.7% experiencing only traditional bullying and 5.1% only cyberbullying. Cyberbullying occurred both independently and alongside traditional bullying, with a 6.1% prevalence for combined victimization. In the total sample, those who experienced combined victimization, reported the highest internalizing symptoms. |
| [29] | Cosma 2020 | 37 (11) | An adapted version of the Olweus Bullying Questionnaire (1997)  Cybervictimization (2014 survey cycle only): participants were asked to indicate how often in the past couple of months they had experienced the following: Someone sent mean instant messages, wall postings, emails and text messages or created a website that made fun of me and Someone took unflattering or inappropriate pictures of me without permission and posted them online. | HBSC | N= 764518, age=11/13/15, Self | Traditional bullying victimization among boys declined in 21 countries, and among girls in 12 countries. Cybervictimization rates were consistently lower than traditional bullying. Notably, 45.8% of students experiencing cybervictimization also faced traditional bullying, with similar percentages for boys (46.5%) and girls (45.3%). |
| [30] | Cosma 2022 | 15 (8) | WHO-5 Well-being Index (Blom, 2012) | HBSC | N=74071, age=11/13/15, Self | LMICs: 2.54 (1.21) (Turkey)- 3.46 (1.16) (Armenia) Non-LMICs: 2.75 (1.19) (Austria)- 3.05 (1.08) (Lithuania) WHO-5 does not show good psychometric properties and measurement invariance. The WHO-4, excluding the first item of the scale (I have felt cheerful and in good spirits), demonstrated good psychometric properties. |
| [31] | Cosma 2022 | 46 (13) | Traditional bullying perpetration and victimization: Olweus Bullying Questionnaire (1997) Cybervictimization: 2 items from HBSC questionnaire | HBSC | N=220457, age=11/13/15, Self | Significant cross-national variations exist in gender differences in bullying. Boys are more likely than girls to engage in both traditional and cyberbullying and to be victims of traditional bullying. In countries with greater gender inequality, these gender differences in traditional bullying are more pronounced. In countries with lower gender inequality, gender disparities in cyber victimization are larger, with girls more frequently becoming victims of cyberbullying than boys. |
| [32] | Craig 2009 | 40 (11) | Traditional bullying perpetration and victimization: Olweus Bullying Questionnaire (1997) | HBSC | N = 202056, age = 11/13/15, self | Adolescents in Baltic countries experienced the highest bullying and victimization rates, while northern Europeans had the lowest. |
| [33] | Craig 2020 | 42 (8) | Cyberbullying perpetration and victimization: Olweus Bullying Questionnaire (1993) | HBSC | N = 180919, age = 11/13/15, self | Engagement in social media use was associated with cyberbullying victimization and perpetration. These association were stronger for cyber-perpetration versus cyber-victimization and for girls versus boys. Problematic social media use was most strongly and consistently associated with cyber-bullying, both victimization and perpetration. |
| [34] | Crocetti 2015 | 6 (4) | GAD subscale of the SCARED: Screen for Child Anxiety Related Emotional Disorders (Birmaher, 1997) | N/A | N = 3445, age = 14-18, self | The scores on GAD symptoms varied significantly across countries, with Dutch respondents reporting the lowest levels whereas Filipino participants exhibited the highest levels of GAD symptoms. Girls reported more GAD symptoms than boys in all countries except Kenya. |
| [35] | Crous 2017 | 15 (7) | Psychological well-being multi-item (PWB, Ryff's scale, 1989) | ISCWEB | N = 18286, Mage=12.05 (SD:0.595), Self | PWB mean scores LMICs: 8.14 (1.51) (Ethiopia)- 9.04 (1.21) (Romania) Non-LMICs: 7.43 (1.92) (S Korea)- 8.85 (1.37) (Malta) More than 25% of the deprived children reported low PWB (e.g., 69% in S Korea). |
| [36] | Delvecchio 2015 | 2 (1) | The Spence Children's Anxiety Scale (SCAS; Spence, 1997) The Strengths and Difficulties Questionnaire (SDQ; Goodman, 1997) | N/A | N = 3416, age = 13-18, self | Higher levels of anxiety symptoms were significantly associated (p<.01) with both the internalizing and externalizing behavior factors. Chinese adolescents reported higher anxiety symptoms than Italian ones and girls scored higher than boys. No age differences were found. |
| [37] | Deryol 2022 | 23 (5) | Traditional bully victimization; How often have you been bullied at school in the past couple of months? Cyberbully victimization; How often someone sent mean instant messages, wall postings, emails, and text messages, or created a website that made fun of her/him? How often someone took unflattering or inappropriate pictures of her/him without permission and posted them online? | HBSC | N = 110718, Mage = 13.55, Self | Individual-level factors associated with traditional and cyberbullying victimization vary across countries. Notably, nations with higher human development levels tend to report lower instances of both forms of bullying. Furthermore, the quality of human development at the country level influences how individual factors relate to experiences of traditional and cyberbullying victimization |
| [38] | DiGiunta 2018 | 3 (1) | Child Behavior Checklist (CBCL; Achenbach, 1991) Youth Self-report (YSR; Achenbach, 1991) | N/A | N = 534, age = 10/12/13,  Self & parents | Higher maternal self-efficacy in anger regulation was associated with stronger adolescent self-efficacy, reducing internalizing and externalizing symptoms. Cultural variations were minimal, but fathers’ influence on adolescent self-efficacy was less consistent across contexts. |
| [39] | DiGiunta 2020 | 9 (6) | Child Behavior Checklist (CBCL; Achenbach, 1991) Youth Self-report (YSR; Achenbach, 1991) | N/A | N = 1298, age = 13-15, self & parents | Parental irritability and low self-efficacy in anger regulation were associated with harsher parenting and increased adolescent irritability. Adolescents’ irritability mediated the relationship between parental emotional regulation and both internalizing and externalizing problems. These associations were consistent across nine countries, highlighting cultural similarities in the impact of parental emotionality on adolescent adjustment. |
| [40] | DiGiunta 2023 | 2 (1) | Parental Warmth: Parental Acceptance-Rejection/ Control Questionnaire (Short Form) Harsh Parenting: The Discipline Interview Youths' externalizing and internalizing problems: Achenbach’s (1991) Youth Self- Report; the Adult Self-Report (ASR; Rescorla and Achenbach, 2004)  Youths' anger and sadness: the Early Adolescent Temperament Questionnaire-Revised (EATQ-R; Capaldi and Rothbart, 1992)  Youths' self-eficacy beliefs in regard to anger and sadness regulation: the Regulative Emotional Self-Efficacy Scale (Caprara et al., 2008) | N/A | N = 285, age = 10-18, self & parents | Adolescents in Colombia and Italy showed increasing self-efficacy for anger regulation, while self-efficacy for sadness regulation remained stable. Harsh parenting and externalizing problems at age 10 predicted lower initial anger regulation self-efficacy. High self-efficacy for both emotions at age 12 was linked to fewer internalizing and externalizing problems at age 18. |
| [41] | Dmitrieva 2004 | 4 (2) | Perceived Parental Warmth Scale (11 items); Parental Knowledge Scale (10 items); Parent-adolescent Conflict Scale (11 items); Perceived Parental Sanction of Adolescent Misconduct Scale (11-items); Family-related negative life events (7 items); Center for Epidemiological Studies Depression Scale (CES_D)(20 items); Adolescent problem behaviours Scale (45 items) | N/A | N = 1696, age = 16.47-17.64, self | The path from family-related life events to adolescent problem behaviors was mediated by perceived parental involvement, parent–adolescent conflict, and perceived parental sanctions of adolescent misconduct. With the exception of minor cross-cultural differences in the magnitude of associations among variables, this study revealed considerable similarity in the association of family factors with adolescent internalizing and externalizing symptomatology. |
| [42] | Doty 2023 | 2 (1) | Cyberbullying and cyber victimization: the Problem Behavior Frequency Scales- Adolescent Revised (Farrell et al., 2018) | N/A | N = 485, age = 12.47 (India)&11.79 (U.S.), self & parents | Strong invariance was found for perpetration, but weak invariance for victimization highlights limits in cross-cultural comparisons. Cyberbullying prevalence was 34.9% in India and 31.1%-35.0% in the U.S., with SES influencing victimization patterns in India. These findings support culturally tailored approaches for research and intervention. |
| [43] | Due 2005 | 28 (8) | Bullying: During this term, how often have you been bullied at school?"  Health Status: Self-reported frequency of twelve symptoms:  Headache, stomachache, backache, feeling low, bad temper, nervousness, difficulties in getting to sleep, and dizziness, loneliness, Tired in the morning, Feeling left out of things, Feeling helpless  Family Affluence:  Does your family own a car, van, or truck?  Do you have your own bedroom?  During the past twelve months, how many times did you travel away on holiday with your family? | HBSC 1997/1998 | N = 123227, age = 11/13/15, Self | Bullying prevalence varied widely, from 6.3% in Sweden to 41.4% in Lithuania. A consistent and graded relationship was observed, where higher bullying exposure increased the risk of symptoms like headache, feeling low, and helplessness. |
| [44] | Due 2008 | 66 (36) | Bullying victimization: How often have you been bullied at school in the past couple of months? (HBSC) / During the past 30 days, on how many days were you bullled? (GSHS) | HBSC 2001/2002; GSHS | N = 218104, age = 13-15, Self | Bullying prevalence varied significantly, with averages of 32.1% (HBSC) and 37.4% (GSHS). The highest rates were found in Lithuania (62.7%) and African countries, while the lowest were in Sweden (16.0%) and Tajikistan (7.1%). Prevalence was generally higher among boys but showed regional and gender-specific variations. Bullying was strongly associated with negative social, physical, and psychological outcomes. |
| [45] | Due 2009 | 35 (11) | The Olweus Bullying Questionnaire The Family Affluence Scale (FAS) | HBSC 2001/2 | N = 142911, age = 11/13/15, Self | Children from lower socioeconomic backgrounds were at greater risk, with a 13% higher likelihood of being bullied for each step decrease in family affluence. Greater income inequality, measured by a 10-point increase in the Gini coefficient, was associated with a 3% higher likelihood of bullying. National wealth and school-level economic status did not directly influence prevalence, but disparities in income within schools and countries significantly increased bullying rates. |
| [46] | Duinhof 2020 | 7 (2) | The Strengths and Difficulties Questionnaire (SDQ; Goodman, 1997) | HBSC | N = 33233, age = 11,13,15, self | Adolescents in different countries reported varying psychological and behavioral challenges. Emotional symptoms and conduct problems were highest in Poland, while Greece and Bulgaria reported the lowest levels of emotional symptoms. Peer relationship problems were most prevalent in Bulgaria, Germany, and Slovenia, while Greece reported the lowest. Adolescents in the Netherlands had the fewest conduct problems but the highest levels of hyperactivity-inattention issues. |
| [47] | Dzielska 2020 | 26 (2002=8/ 2006=7/2010=4) | Weight Reduction Behavior: At present, are you on a diet or doing something else to lose weight?  Body Mass Index Body Image Body Weight congruence | HBSC 2017/2018 | N= 639194 age= 11/13/15  Self | The prevalence of WRB was higher among girls than boys, though gender differences narrowed in recent surveys. Overall rates were 10.2% for boys and 18.0% for girls. WRB increased significantly among boys in most countries, while changes among girls were less common. Higher BMI and weight overestimation were key risk factors for WRB in both genders. |
| [48] | Elgar 2009 | 37 (14) | The Family Affluence Scale (FAS); Social support (family; peer; school support) Olweus’s Bully/Victim Questionnaire | United Nations Development Program Human Development Report / HBSC (2005/2006) | N = 66817, age = 11, Self | Countries with higher income inequality experience more school bullying among preadolescents, with males and females both affected, while family and school support reduce bullying but do not explain the link between inequality and bullying, emphasizing the need for targeted anti-bullying efforts across socioeconomic contexts in both HICs and LMICs. |
| [49] | Elgar 2015 | 79 (46) | GSHS:  Bullying: In past 30 days, on how many days were you bullied?  Fighting: In past 12 months, how many times were you in a physical fight?  HBSC:  Bullying: How often have you been bullied at school in the past couple of months?  Fighting: In past 12 months, how many times were you in a physical fight? | GSHS 2003-2011/ HBSC 2006/2010 | N = 334736/342312, age = 11-16, Self | Approximately 30% of adolescents reported being bullied, and 10.7% of boys and 2.7% of girls engaged in frequent physical fighting. Higher country wealth was associated with lower bullying rates, reducing prevalence by 3.9% for boys and 4.2% for girls per unit increase in wealth. For fighting, wealth decreased rates by 2.9% for boys and 1.0% for girls. Income inequality and education spending influenced these patterns, with fighting less prevalent in affluent but unequal countries that invested more in education. |
| [50] | Erskine 2024 | 3 (3) | DISC-5 (Youth Risk Behaviour Surveillance System) | N/A | N=16815, age=10-17, Self & Parents | Suicidal behaviors and self-harm were significantly higher among individuals with mental disorders, with a 12-month prevalence of any mental disorder ranging from 3.3% in Vietnam to 12.1% in Kenya. Suicide attempts in the past year were less frequent in Vietnam and Indonesia (0.2%) compared to Kenya (1.0%). |
| [51] | Eskin 1995 | 2 (1) | The Suicide Probability Scale (SPS) (Cull and Gill, 1988); The scale for Interpersonal Behavior (SIB); Perceived Social Support (PSS) | N/A | N = 1306, age = 15-20, self | Suicide attempts were reported by 9.4% of Swedish and 10.9% of Turkish students. Key risk factors included previous psychiatric contact, low family support, suicide attempts in the family, and female gender. Among Swedish adolescents, low peer support, few friendships, and low assertiveness were also significant predictors of suicidal risk. Family-related stress was a major factor for both groups, but Turkish adolescents reported more school-related and life-stress reasons for attempting suicide. |
| [52] | Eskin 2014 | 2 (1) | Suicidal ideation: Have you ever thought of killing yourself? Have you, during the past 12 months, thought of killing yourself? Do you have thoughts of killing yourself right now? Have you ever made an attempt to kill  yourself? Have you, during the past 12 months, made an attempt to kill yourself? Current Mood: How sad do you feel right now? Religiosity: What is the depth of your religious belief?  Attitudes Toward Suicide: 24 items  Reactions to an Imagined Suicidal Close Friend: 20 items | N/A | N = 964, age = 14-20, self | Suicidal ideation rates were similar between groups, with 36.4% of Slovak and 33.8% of Turkish students reporting lifetime, past 12-month, or current thoughts. However, suicide attempts were more common among Turkish students (12.2%) than Slovak students (4.8%). Slovak adolescents had more permissive attitudes toward suicide, while Turkish students, who rated themselves as more religious, were more likely to believe in punishment after death. Despite this, Turkish students were more accepting of a suicidal close friend compared to their Slovak peers. |
| [53] | Eslea 2004 | 7 (1) | The Olweus Questionnaire for bullying: How often have you been bullied at school this term?’’; ‘‘How often have you taken part in bullying other children at school this term? | N/A | N = 47992, age = around 11,  self | Victimization rates varied widely, with the lowest reported in Ireland (5.2%) and the highest in Italy (Florence) at 25.6%. The proportion of students identifying as bullies ranged from 2.0% in China to 16.9% in Spain. Bully-victims, who both bullied others and were bullied, made up 19.6% of students in Spain but only 0.8% in England (national sample). The percentage of students uninvolved in bullying was highest in Ireland (91.0%) and lowest in Spain (50.8%). |
| [54] | Farruggia 2004 | 2 (2) | Self-esteem: Rosenberg Self-Esteem Scale (1965) Parental warmth and acceptance: 11-item scale (Greenberger, Chen, & Beam, 1998). Depressed mood: 20-item Center for Epidemiologic Studies Depression Scale (CES-D Scale; Radloff, 1977). | N/A | N = 1911, age = Mean range (16.5-17.6), self | The study examined adolescent self-esteem across four countries, finding higher levels in the U.S. (mean score 3.8) and China (3.6) compared to the Czech Republic (3.2) and Korea (2.9). Parental warmth was strongly correlated with self-esteem in all groups (r = 0.51 to 0.35), with the strongest link in the U.S. (r = 0.51) and the weakest in the Czech Republic (r = 0.35). Self-esteem was negatively associated with depressive symptoms, with the effect stronger in individualistic cultures. |
| [55] | Fine 2022 | 4 (4) | Psychosocial risk indicators (10 items); Risk and protective factors (10 items); Adverse Childhood Experiences (ACEs) | N/A | N = 10437, age = 11-14, Self & primary caregivers | In the Democratic Republic of Congo, bullying raised behavioral problem risks by 13.67 times, while in Malawi, feeling unsafe in the neighborhood increased maladjustment by 2.69 times. In Indonesia, bullying was linked to a 10.78 times higher risk of behavioral problems, and in China, adverse childhood experiences doubled the risk. These findings highlight the need for interventions addressing bullying, school safety, and early adversity. |
| [56] | Fine 2023 | 4 (4) | Emotional problems: I blame myself when things go wrong; I worry for no good reason; I am so unhappy I can't sleep at night; I feel sad; I am so unhappy I think of harming myself.  Behavioral problems: Bullied or threatened another boy or girl for any reason; Slapped, hit, or otherwise physically hurt another boy or girl; Been teased or called names by someone; Been slapped, hit, or otherwise physically hurt by a boy or girl. | N/A | N = 10437, age Mean range = 11.9-12.5, self | A four-group classification emerged: Well-Adjusted (40–62%), Emotional Problems (14–29%), Behavioral Problems (15–22%, absent in China), and Maladjusted (4–15%). Boys were more likely to have behavioral problems, while girls were more prone to emotional issues. In the Democratic Republic of Congo, 22% of adolescents showed behavioral problems, while in Malawi, 15% fell into the maladjusted category. In Indonesia, 29% experienced emotional problems, and in China, 62% were well-adjusted. |
| [57] | Fismen 2022 | 47 (13) | Weight status: How much do you weigh without clothes? How tall are you without shoes?  Self-Percieved body weight: Do you think your body is...? Life satisfaction: Here is a picture of a ladder. The top of the ladder ‘10’ is the best possible life for you and the bottom ‘0’ is the worst possible life for you. In general, where on the ladder do you feel you stand at the moment?  Subjective health complaints: how often they experienced the following symptoms over the past 6 months: headache, abdominal pain, backache, feeling low, irritability or in a bad mood, feeling nervous, sleeping difficulties, and dizziness. | HBSC 2017/2018 | N= 64229, age= 15,  Self | Adolescents who perceived themselves as "too fat" had significantly lower life satisfaction (b = -0.73) and more subjective health complaints (b = -0.34), regardless of actual weight status. The likelihood of self-perceiving as overweight was highest in those with obesity (OR = 32.64) and overweight (OR = 9.06). Girls were more likely to feel "too fat" (OR = 3.16), while those with lower socioeconomic status had a higher risk of both feeling overweight (OR = 1.11) and underweight (OR = 1.13). |
| [58] | Fleming 2010 | 19 (18) | Bullying victimization: During the past 30 days, how many days were you  bullied?  mental health and health behaviors: During the past 12 months, did you ever feel sad or hopeless almost every day for two weeks or more in a row that you stopped doing your usual activities? During the past 12 months, how often have you felt lonely? During the  past 12 months, how often have you been so worried about something that you could not sleep at night? During the past 12 months, did you ever seriously considered attempting suicide? along with questions about age of first cigarette the frequency of drinking alcohol within the past 30 days, and illegal drug use. | GSHS 2003-2006 | N = 91398, age = 12-16, Self | The study examined bullying among middle-school students in low- and middle-income countries, with prevalence ranging from 7.8% in Tajikistan to 60.9% in Zambia. Boys and younger students were more likely to report being bullied. Victimized students had higher risks of sadness (45.6% vs. 27.6%), loneliness (78.0% vs. 63.4%), and suicidal ideation (25.5% vs. 16.0%) compared to non-bullied peers. They also reported increased tobacco use (31.6% vs. 15.3%), alcohol use (31.6% vs. 15.3%), drug use (13.3% vs. 4.3%), and sexual activity (22.7% vs. 10.4%). |
| [59] | Frenzel 2007 | 2 (1) | The Academic Emotions Questionnaire “Mathematics (AEQ-M) (Pekrun et al., 2005) | N/A | N = 891, age Mean range = 14.34-14.99, self | Chinese students were found to experience higher levels of anxiety in mathematics. They were also found to experience more enjoyment, pride, and shame, and less anger, than German students. |
| [60] | Germani 2021 | 2 (1) | The meaning in Life Questionnaire (MLQ, Steger et al., 2006) Family Allocentrism Idiocentrism Scale (FAIS; Lay et al., 1998) Children’s Depression Inventory (CDI; Kovacs, 1992) | N/A | N = 415, age Mean range = 12.59-12.60, self | Chinese adolescents scored higher in family allocentrism (M = 73.82) than Italian peers (M = 67.37). In both countries, family allocentrism was negatively associated with depressive symptoms (China: β = -0.34, Italy: β = -0.28), with meaning in life mediating this relationship. The mediation effect was stronger in Italy, where 34.5% of the total effect of family allocentrism on depressive symptoms was explained by meaning in life, compared to 30.5% in China. |
| [61] | Ghekiere 2019 | 33 (2002=10/2006=8/2010=5/2014=3) | Sleep-Onset Difficulties: "How often in the past six months did you have difficulties in falling asleep?"  Physical Activity: "On how many days in the past week were you physically active for at least 60 minutes?"  Screen Time Behavior:  "How many hours a day, in your free time, do you usually spend watching TV, videos, DVDs, and other entertainment on a screen?"  "How many hours a day, in your free time, do you usually spend playing games on a computer, console, tablet, smartphone, or other electronic device?"  "How many hours a day, in your free time, do you usually spend using electronic devices for purposes such as homework, emailing, social media, or browsing the internet?" | HBSC | N(2002) = 155300  N(2006) = 167656  N(2010) = 172729  N(2014) = 175399,   age= 11,13,15 Self | Sleep difficulties increased over time, with prevalence reaching 37.4% in France and as low as 9.6% in Ukraine. Excessive screen time, exceeding two hours daily, was linked to a 20% higher likelihood of sleep difficulties, with rates surpassing 90% in many countries. Physical activity levels slightly improved but showed no direct association with sleep problems. |
| [62] | Gillé 2021 | 9 (3) | Stress and coping Questionnaire for Children and Adolescents (SSKJ 3-8 R)  The Strengths and Difficulties Questionnaire (SDQ; Goodman, 1997) | N/A | N=5227, age=7-18, Self | Total difficulties scores ranged from 0.56 in Russia and Ukraine to 0.67 in Spain. Girls scored higher in somatic symptoms, sadness, anxiety, and anger, with well-being differences by country. In LMICs, anger scores were highest in Ukraine (6.99), sadness peaked in Russia (7.37), and well-being was highest in the Dominican Republic (10.96). In non-LMICs, anger was highest in English-speaking countries (7.90), sadness in English-speaking countries (6.88), and well-being highest in Germany (10.78). |
| [63] | Gobina 2008 | 2 (2) | Bullying Experience: "How often have you been bullied at school in the past couple of months?"; "How often have you taken part in bullying another student(s) at school in the past couple of months?"  Self-Rated Health: "How would you rate your health?"  Health Complaints: Headache; Stomach ache; Backache; Nervousness; Irritability or bad temper; Difficulties in getting to sleep; Dizziness  Life Satisfaction: Measured using the 10 steps of Cantril’s ladder. | HBSC 2001/2002 | N = 3417, age = 11/13/15, Self | Bullying involvement was significantly higher in Lithuania (52.3%) than in Latvia (30.1%). Boys were more likely than girls to be bullies or bully-victims in both countries. Adolescents involved in bullying had poorer self-rated health, with victims (OR = 2.29) and bully-victims (OR = 2.17) being the most affected. Victims reported more frequent health complaints such as nervousness (OR = 2.29), headaches (OR = 1.80), and difficulty sleeping (OR = 1.92). Low life satisfaction was also linked to bullying, with victims (OR = 2.61) and bully-victims (OR = 2.39) at higher risk. |
| [64] | Gomez-Baya 2022 | 15 (7) | School Bullying and Exclusion: "How often, if at all, in the last month have you been hit by other children in your school?" "How often, if at all, in the last month have you been left out by other children in your class?"  Subjective Happiness: "Overall, how happy have you been feeling during the last two weeks?" | ISCWeB | N = 12623, age = 10, Self | 20.8% of the total sample suffered bullying twice or more. The countries with the greatest percentages for bullying were South Africa, Turkey and Malta, while the countries with the lowest percentages were South Korea, Algeria and Norway |
| [65] | Görzig 2017 | 18 (2) | Cyber- and Face-to-face bullying victimization: Has someone acted in this kind of hurtful or nasty way to you in the PAST 12 MONTHS? | the 2010 EU Kids Online study | N = 15813, age = 12.43, Self/Parent(s) | Cyberbullying victimization rates ranged from 2% to 14% across countries. Life expectancy at the regional level was negatively associated with cyberbullying (β = -0.31), while crime rates showed a marginal positive relationship (β = 0.10). Higher population density was linked to lower cyberbullying (β = -0.14), whereas GDP was positively associated with cyberbullying (β = 0.26). |
| [66] | Govorova 2020 | 35 (2) | The cognitive performance scale | PISA | N = 248620, age = 15, self | Cognitive well-being, including enjoyment of science and self-efficacy, was the strongest predictor of academic performance, increasing science scores by up to 22 points. However, school effects on overall well-being were minimal, accounting for only 5–9% of variance. Socioeconomic status remained a key predictor of well-being and performance. |
| [67] | Greenberger 2000^f^ | 2 (1) | 20-item Center for Epidemiological Studies Depression Scale (CES-D) | N/A | N = 703,  age = 16-18, self | Gender differences were more pronounced in the U.S., where girls reported significantly higher depressive symptoms than boys. In China, parental warmth (β = -0.34) and parent-child conflict (β = 0.32) had stronger effects on depression than in the U.S. (β = -0.21, β = 0.18). Academic achievement was also more closely linked to depressive symptoms in China (β = -0.26) than in the U.S. (β = -0.09). |
| [68] | Greenberger 2000^g^ | 3 (1) | Adolescents' problems Behaviors; Perceived behavior of others; Perceived attitudes of parents and close friends toward the adolescent's hypothetical involvement in misconduct | N/A | N = 1094,  age = 16-17, self | Gender differences in depressive symptoms were more pronounced in the U.S., with higher rates among girls (β = 0.18). In China, parental warmth (β = -0.34) and parent-child conflict (β = 0.32) had stronger associations with depressive symptoms than in the U.S. (β = -0.21, β = 0.18). Academic achievement showed a stronger negative correlation with depressive symptoms in China (β = -0.26) compared to the U.S. (β = -0.09). |
| [69] | Gross-Manos 2022 | 35 (14) | The Subjective well-being scale (SWBS): I enjoy my life; My life is going well; I have a good life; The things that happen in my life are excellent; I am happy with my life | ISCWEB | N = 49428, age=10/12, Self | South Korea emerged as an outlier, showing low subjective well-being despite high material well-being. Countries such as Switzerland, India, and Israel displayed weaker associations between deprivation and well-being. In Israel, children in rural areas were found to be materially poorer but reported higher happiness than urban counterparts. |
| [70] | Güngör 2010 | 2 (1) | Parental warmth and control: The 22-item Perceived Parenting Styles Scale (Sumerr & Gungo r, 1999b); Attachment avoidance and anxiety with closest friends and romantic partners: 34 items from the 36-item Experiences in Close Relationships Inventory (ECR; Brennan et al., 1998) | N/A | N = 533, age = 14-18, self | Turkish adolescents reported higher anxiety than Belgian adolescents, though both groups had relatively low average scores (below 2.50). Maternal and paternal warmth were negatively correlated with attachment avoidance and anxiety, while psychological control showed positive associations in both groups. Increased warmth and decreased control were linked to lower attachment anxiety and avoidance. In the total sample, female adolescents reported higher attachment anxiety than males, and anxiety increased with age. |
| [71] | Gupta 2013 | 2 (1) | The Gender-Typed Behavior in Relationships Scale (GBRS); The Rosenberg Self-Esteem Scale (Rosenberg, 1965); The Children’s Depression Inventory (CDI; Kovacs, 1992); Perceived Social Support from Friends measure (Procidano & Heller, 1983) | N/A | N = 814, age Mean rang (11.37-12.20), self | Adherence to gender-typed behaviors in friendships was linked to higher depressive symptoms, lower self-esteem, and lower friendship quality for boys in both countries. The association with depressive symptoms was stronger for boys in the United States. No significant nationality differences were found in gender-typed behaviors, depressive symptoms, or friendship quality over two years. |
| [72] | Haid 2010 | 3 (1) | The Problem Questionnaire (PQ; Seiffge-Krenke 1995) | N/A | N=3259, age=11-20, Self | Italian adolescents reported the highest future-related stress (M = 0.79), followed by Turkish (M = 0.55) and German adolescents, who had the lowest (M = 0.18). A similar pattern was observed for identity-related stress, with Italian adolescents scoring highest (M = 0.63), followed by Germans (M = 0.39) and Turks (M = 0.01). |
| [73] | Han 2021 | 6 (1) | Student's sense of school belonging; Parental support; Victimization | PISA | N = 33633, age = 15, Self | The prevalence of bullying victimization was highest in Hong Kong (31.2%) and lowest in South Korea (9.7%). Latent profile analysis identified three victimization patterns, with “made fun of” being the most frequently reported form (38.5%). Parental support moderated the relationship between victimization and school belonging only in Taiwan (β = 0.27, p < .05), where higher parental support reduced the negative impact of bullying. |
| [74] | Harel-Fisch 2010 | 2 (1) | STAGE (Subjective threat from armed conflict events); Child posttraumatic Stress Reaction Index (CPTS-RI); Cantril ladder; Parental Support | HBSC | N = 24935, age = 11,13,15, self | Subjective threat scores were highest in Gaza (M = 14.6) and lowest in Jewish Israelis (M = 5.23). Higher exposure was associated with increased posttraumatic symptoms (Jewish Israeli: β = 0.26, West Bank: β = 0.20, Gaza: β = 0.20) and psychosomatic symptoms (Jewish Israeli: β = 0.15, West Bank: β = 0.13). Smoking rates were highest in the West Bank (M = 0.17) and lowest in Gaza (M = 0.09). |
| [75] | Harel-Fisch 2011 | 40 (11) | Olweus Bullying Questionnaire; School perceptions | HBSC 2002/2006 | N = 197502, age = 11/13/15, Self | Negative school perceptions were strongly associated with bullying involvement across 40 countries. Adolescents with 2–3 negative school perceptions had twice the odds of being involved in bullying compared to those with no negative perceptions. The odds increased in a graded fashion, reaching over 8 times higher for those with the most negative perceptions. The association was consistent across gender and nearly all countries, with the strongest effects found in Iceland (OR = 23.3 for victimization) and Sweden (OR = 11.8 for bullying). |
| [76] | Harel-Fisch 2012 | 2 (1) | Suicidal ideation and behavior (4) Risk behavior (Health related risk behaviors; Negative influence in social settings; School Achievement) (HBSC-ME 2004 survey) | HBSC | N = 8345, age = 15, Self | Suicidal ideation and behavior were highest among Arab-Israeli adolescents (20.5% ideation, 17.2% planning, 17.7% attempts), followed by Jewish Israelis (17.4%, 9.1%, 7.8%) and Palestinians (14.5%, 11.2%, 11.4%). |
| [77] | Heinz 2020 | 45 (12) | Low Life Satisfaction: Cantril’s ladder  Multiple Health Complaints: In the past six months, how often have you had the following? (Headache, Abdominal pain, Backache, Feeling low, Irritability, Nervousness, Sleeping difficulties, Dizziness)  Body Image Perception: "Do you feel that your body is…"  School Pressure: "How pressured do you feel by schoolwork?"  Bullying Victimization: "How often have you been bullied at school in the past couple of months?"  Low Family Support: "How easy is it for you to talk to your parents or guardians about things that really bother you?" "How much do your parents or guardians help and support you when needed?" "Do you feel that your family understands you?" "How much does your family care about you?" | HBSC 2018 | N = 71942, age = 15, Self | Girls had higher odds of reporting multiple health complaints (OR = 2.51), feeling fat (OR = 1.95), and school pressure (OR = 1.71) compared to boys. Smoking and alcohol use were more common among boys in most countries, but in high-gender-equality countries, gender gaps in substance use were smaller. Nordic countries had some of the highest odds for school pressure (e.g., Sweden OR = 2.90). |
| [78] | Hillekens 2020 | 4 (1) | Network of the relationship Inventory; Youth self-Report Questionnaire | N/A | N = 1026, age Mean range = 10.68-11.22, self | No cross-cultural differences in associations between quality of the parent-child relationship and problem behavior. We did not find any effects of maternal or paternal warmth. However, across samples conflict with mothers was associated with more internalizing and externalizing problem behavior, and conflict with fathers was associated with more externalizing problem behavior |
| [79] | Högberg 2021 | 35 (10) | Stress related demands in School: How pressured do you feel by the schoolwork you have to do? | HBSC | N = 160000, age = 15-16, self | Economic change, measured by GDP growth, but not educational expansion, contributes to increased school stress in adolescents. Both economic growth and educational expansion amplify the impact of stress on mental health, making the effect stronger as countries become wealthier and more educated. Girls generally experience higher stress levels across most years and countries, but stress levels remain relatively stable over time. |
| [80] | Hosozawa 2021 | 71 (30) | During the past 12 months, how often have you had the following experiences in school? (Some experiences can also happen in social media.)’:  1 Other students left me out of things on purpose.  2 Other students made fun of me.  3 I was threatened by other students.  4 Other students took away or destroyed things that belonged to me.  5 I got hit or pushed around by other students.  6 Other students spread nasty rumours about me. | PISA | N = 421437, age = 15, Self | 30.4% experienced frequent victimization, with rates varying by country from 9.3% in Korea to 64.8% in the Philippines. Verbal and relational victimization were more common (21.4% and 20.9%, respectively) than physical victimization (15.2%). Boys, students from the lowest wealth, and those with lower academic performance had higher victimization scores, but there was substantial country-to-country variation. Similar patterns were observed for all victimization types, except for relational victimization, where gender differences were smaller. |
| [81] | Hussein 2010 | 3 (1) | The Peer Interactions in Primary School Questionnaire (Tarshis and Huffman 2007) | N/A | N=981, age=around 11, Self | Egyptian and Saudi boys/girls had a higher level of bullying than American boys/girls, whereas no differences were displayed among the three cultures on the victimization subscale. Boys had a higher level of bullying than girls in the three cultures, and boys and girls had a similar level of victimization in three cultures. |
| [82] | Imran 2021 | 2 (1) | The Hospital Anxiety and Depression Scale (HADS; Zigmond & Snaith, 1983) The Psychological Well-being Scale (Ryff, 1989) | N/A | N=1120, age=12-18, Self | Psychological well-being scores were slightly higher in Scotland (M = 110.49, SD = 18.97) than in Pakistan (M = 108.77, SD = 24.72), while anxiety and depression scores were also higher in Scotland (M = 28.10, SD = 7.21) compared to Pakistan (M = 26.12, SD = 6.97). |
| [83] | Isaksson 2023 | 3 (1) | the Eating Disorder Diagnostic Scale; the Screening Survey of Exposure to Community Violence; the Child Post-Traumatic Stress Reaction Index (CPTS-RI); the Center for Epidemiologic Studies-Depression Scale (CES- D); Anxiety: a 12-item scale [27] which targets worrisome and preoccupying thoughts or unpleas- ant feelings about the student him/herself or about external stimuli. | the Social and Health Assessment (SAHA) | N= 9751, age= 12-18, Self | No differences were found between the countries in the occurrence of ED thoughts (p=0.061). However, the frequency of ED compensatory behaviours differed between the countries (p<0.001), with higher ratings among students in HICs than in LMICs. |
| [84] | Iwawaki 2001 | 2 (1) | Trait scale of STAIC X-2 (Spielberger, 1973);  Sleeping patterns: real length of sleep, favourable length of sleep, time for going to bed, regular or irregular sleep regime | N/A | N = 818, age = 12-13, self | Girls and older children had higher anxiety scores, with no significant difference between Japan and Slovakia. A negative relationship was found between anxiety and sleep duration in the combined sample. Children who go to bed late had higher anxiety scores. Japanese children needed more sleep to feel refreshed, with a difference of 2.17 hours compared to 1.35 hours in Slovak children. Additionally, Japanese children went to bed later and had shorter sleep durations. |
| [85] | Jacob 2020 | 32 (26) | Suicide attempt: During the past 12 months, how many times did you actually attempt suicide?  Fast food consumption: During the past 7 days, on how many days did you eat food from a fast food restaurant? | GSHS | N = 105061, age = 12-15, self | The prevalence of suicide attempts was higher among consumers of fast food compared to non-consumers (11.8% vs. 8.3%). The figures were similar for boys and girls. Of the 32 countries included in the study, a positive association (OR > 1) between fast food consumption and suicide attempts was found in 26 countries although this was not statistically significant in all countries. The pooled estimate by country-income level showed that the association is most pronounced in low-income countries, followed by lower middle-income countries, and upper middle-income countries. |
| [86] | Jacob 2020 | 32 (26) | Suicide attempt: During the past 12 months, how many times did you actually attempt suicide?  Consumption of carbonated soft drinks:  During the past 30 days, how many times per day did you usually drink carbonated soft drinks? | GSHS | N = 105061, age = 12-15, self | The prevalence of suicide attempts and carbonated soft drink consumption ≥3 times/day was 10.2% and 10.7%, respectively. After adjusting for confounders, those consuming 3 and ≥4 times/day were 1.36 and 1.43 times more likely to report a suicide attempt. Country analyses revealed that consuming soft drinks ≥3 times/day was linked to higher odds of suicide attempts in 22 of 32 countries, with a pooled odds ratio of 1.20. |
| [87] | Jessor 2003 | 2 (1) | The 36-page Adolescent Health and Development Questionnaire (AHDQ): Protective Factors and Risk Factors; Problem Behavior Involvement | N/A | N = 3335, age = 7-9 grades, self | Despite lower prevalence of the problem behaviors in the Chinese sample, especially for girls, a substantial account of problem behavior is provided by the same protective and risk factors in both countries and for both genders. Protection is generally higher in the Chinese sample than in the U.S. sample, but in both samples protection also moderates the impact of risk. |
| [88] | Johnson 2024 | 53 (17) | School belonging: “I feel like an outsider (or left out of things) at school" (reverse scored), "I make friends easily at school," "I feel like I belong at school," "I feel awkward and out of place in my school" (reverse scored), "other students seem to like me," and "I feel lonely at school" (reverse scored)  Teacher support: the teacher "shows an interest in every student's learning"; "gives extra help when students need it"; "helps students with their learning"; and "continues teaching until students  understand" | PISA | N=413575, age=15.8, self | Students' perceptions of cooperation, bullying, and a disruptive disciplinary climate were key predictors of school belonging. In countries with higher individualism and power distance, students reported stronger belonging, even after accounting for factors like teacher support, bullying exposure, and parental involvement. Higher perceived teacher support and cooperation were associated with greater belonging and lower bullying and parental involvement. |
| [89] | Kahumoku 2011 | 2 (1) | Objectified body consciousness: Body surveillance; Body shame; The desire to change body scale; The eating behaviors scale Control beliefs  Mental health indicators:  1) Depressive symptoms 2) Somatic complaints 3) Suicide ideation | the Swiss Multicentric Adolescent Survey on Health questionnaire | N= 18239, Mean age range = 16.4-17.8,  Self | Swiss participation had a significantly higher means than Georgian participants in Body surveillance, Body shame, Appearance control beliefs, Somatic complaints, and Suicidal ideation while Georgian participants had a significantly higher mean than Swiss participation in Depressive symptoms. |
| [90] | Kakar 2023 | 4 (3) | The discrepancy subscale of the Body Image Ideals Ques- tionnaire (BIQ); the Body Image Questionnaire; the Body Esteem Scale for Adolescents and Adults (BESSA; Mendelson et al., 2001) | N/A | N= 900,  Self | There were significant differences between countries for all variables. Girls in Australia as a HICs country satisfaction (2.80,0.95) than girls in LMICs: China (3.19,0.57), India (3.52,0.90) and Iran (3.67,0.73). Actual-ideal facial discrepancies were related to lower appearance satisfaction only for Iranian girls and higher perceived bodily discrepancies were linked to lower appearance satisfaction only for Australian girls. |
| [91] | Kakar 2023 | 4 (3) | The respective Pressure subscales from SATAQ-4R (Schaefer et al., 2017);  The Thin/Low Body Fat Internalization subscale from the SATAQ-4R;  The Physical Appearance Comparison Scale (Thompson et al., 1991);  the Body Esteem Scale for Adolescents and Adults (BESSA; Mendelson et al., 2001); The Children’s Eating Attitudes Test (chEAT-26; Maloney et al., 1988) | N/A | N= 900, age= 12-18,  Self | Girls in Australia as a HICs country reported higher levels of appearance comparison compared to LMICs countries. Australian participants reported higher levels of disordered eating followed by girls in in India, ­Iran, and then girls in China. |
| [92] | Kalaycıoğlu 2015 | 6 (1) | Socioeconomic Status: Students’ socioeconomic status is assessed using the educational, social, and cultural status (ESCS) index;  Math self-efficacy;  Math Anxiety | PISA | N = 8806, age = 15, self | The relationship between socioeconomic status and mathematics achievement is highest in the Netherlands and lowest in Hong Kong. For all six countries, the most important predictor of mathematics achievement is math self-efficacy. The relationship between math self-efficacy and mathematics achievement is highest for England. |
| [93] | Kapetanovic 2020 | 9 (5) | the Youth Self Report Form; the Youth Knowledge, Disclosure, Control, and Solicitation Scale (Stattin and Kerr 2000) | N/A | N = 2084, age = 13, 15, self | Adolescent secrecy predicted higher psychological problems over time, with secrecy at age 13 linked to increased externalizing problems at age 15 across cultures. Secrecy also showed a positive, reciprocal relationship with externalizing behavior, but no other bidirectional links between parents. |
| [94] | Katsantonis 2021 | 32 (6) | Being bullied:  1. Other students left me out of things on purpose. 2. Other students made fun of me. 3. I was threatened by other students. 4. Other students took away or destroyed things that belonged to me. 5. I got hit or pushed around by other students. 6. Other students spread nasty rumors about me. | PISA | N = 286481, age = 15.78, self | East Asian countries have the lowest latent means of bullying, while Southeast Asian countries have the highest means.  Anglo-Saxon, Eastern European, Mediterranean, South American, and Middle East countries displayed rather higher scores. |
| [95] | Kayano 2008 | 3 (1) | The Eating Attitude Test-26 (EAT-26:26 items) The Drive for Thinness subscale of the Eating Disorder Inventory-2 (EDI-2: 7 items) | N/A | N= 724, age=18.65(Japanese)/15.9(Indian)/15.52(Omani)/15.04(European)/14.61(Filipino), Self | Male participants in LMICs scored significantly higher than HICs both in EAT-26 and drive for thinness. Female in HICs scored higher than in LMICs in Drive for thinness scale, while mixed finding was observed in EAT-26. |
| [96] | Kearns 2022 | 88 (67) | Suicidal ideation: During the past 12 months, did you ever seriously consider attempting suicide?  Suicide plan: During the past 12 months, did you make a plan about how you would attempt suicide?  Suicide attempt: During the past 12 months, how many times did you actually attempt suicide?  Worry-related sleep problems: During the past 12 months, how often have you been so worried about something that you could not sleep at night? | GSHS | N= 332877, age=<11/14 ≥15,  Self | The means of worry-related sleep problems were significantly higher in HICs (1.25) than LMICs (1.02). Worry-related sleep problems were associated with all STBs. |
| [97] | Khan 2021 | 64 (44) | Stress-related sleep disturbance: During the past 12 months, how often have you been so worried about something that you could not sleep at night?  Fast food consumption was assessed with the question: ‘During the past 7 days, on how many days did you eat food from a fast food restaurant?  Consumption of carbonated soft drinks was assessed with the ques- tion: ‘During the past 30 days, how many times per day did you usually drink carbonated soft drinks? | GSHS | N= 175261 Age= 12-15 Self | Prevalence of sleep difficulties was 7.5% including both LMICs and HICs.Carbonated soft drink and fast food intake were strongly associated with stress-related sleep disturbance. |
| [98] | Khan 2022 | 38 (6) | School stress: How pressured do you feel by schoolwork?  School satisfaction: How do you feel about school at present?  Discretional time spent on screen-based activities: (i) “About how many hours a day do you usually watch television (including DVDs and videos) in your free time?”, (ii) “About how many hours a day do you usually play games on a computer or games console (PlayStation, Xbox, GameCube, etc.) in your free time?”, (iii) “About how many hours a day do you usually use a computer for chatting on-line, internet, email- ing, homework etc. in your free time?” | HBSC | N = 191786, age = 11, 13, 15, self | Adolescents watching TV for over 4 hours daily had 31% higher odds of school stress and 36% lower odds of school satisfaction, while prolonged gaming raised stress by 26% and lowered satisfaction by 37%. Computer use had the highest impact, increasing stress by 46% and reducing satisfaction by 39%. |
| [99] | Kim 2021 | 2 (1) | Bullying victimization:  China: the Olweus Bully/Victim Questionnaire (Solberg & Olweus, 2003). Canada: The Multi-Dimensional Bullying Questionnaire (Prabaharan, 2020)  Witnessing: Defending Behaviors Scale (Lambe & Craig, 2020)  Internalizing difficulties: the  Strengths and Difficulties Questionnaire (Goodman, 1997)  Individuals’ psychosocial wellbeing (Only measured in Chinese sample) | N/A | N=2933, grade 5-9 (China)/5-8(Canada), Self | Both victimization and witnessing bullying were linked to lower well-being and higher emotional problems in Chinese and Canadian youth. Clearer rules and fairness in Chinese classrooms protected well-being and reduced the impact of witnessing bullying, which affected Chinese students more. While disciplinary structure boosted well-being for Chinese students, it did not moderate bullying’s impact on Canadian youth. |
| [100] | Kim 2022 | 45 (12) | The Olweus Bullying scale; the Cantril ladder; Parental and teacher support: My family really tries to help me/I feel that my teachers care about me as a person | HBSC | N=230757, age=11/13/15, Self | The study examines bullying, mental health, and the role of supportive adults across 45 countries, finding that both in-person and cyber-bullying are strongly linked to higher psychological symptoms and lower life satisfaction. While supportive adults (parents or teachers) were generally associated with better mental health outcomes, their presence paradoxically increased the mental health risks of bullying involvement. Adolescents with multiple supportive adults experienced the highest risks, suggesting complexities in how adult support interacts with the distress of bullying experiences. |
| [101] | King 2024 | 51 (11) | The index of school-work anxiety (OECD, 2017); Achievement scores in three subjects: reading, math, and science | PISA | N = 389215, age = 15, self | Students in more unequal countries experienced greater test anxiety and had lower levels of achievement. Test anxiety, in turn, was associated with lower academic achievement in reading, math, and science. However, test anxiety did not mediate the effects of income inequality on achievement, nor did income inequality moderate the relationship between test anxiety and achievement. |
| [102] | Klinger 2015 | 18 (5) | School pressure: How pressured do you feel by the schoolwork you have to do? | HBSC | N = 453369, age = 11, 13, 15, self | North America reported the highest pressure (e.g., up to 53% in the U.S.), while Germanic countries reported the lowest (e.g., 19-26%). Pressure increased with age and was higher among girls, particularly at age 15, where girls experienced more pressure than boys across all regions. |
| [103] | Koenig 2021 | 4 (4) | Depressive symptom index (6 items); sexual double standard (SDS);  gender stereotypical traits (GST) | GEAS | N = 5749, age = 10-14, self | Depressive symptom mean scores were highest in Denpasar (mean: 11.4) and lowest in Flanders (mean: 7.6). Mean depressive symptom scores were significantly higher among girls than boys in all sites, with the exception of Denpasar, where we observed no significant sex difference. |
| [104] | Kokkevi 2012 | 17 (6) | Suicide attempts: Has it ever happened that you attempted suicide? If so, how many times?  Self-harm thoughts: Have you ever thought of harming yourself?  Current smoking: How frequently have you smoked cigarettes during the last 30 days?  Family structure: hich of the following people live in the same household as you? | ESPAD | N = 45806, age = 15-16, self | The study highlights substantial variation in suicide attempt rates among countries, ranging from 4.1% in Armenia to 23.5% in Hungary, with a median of 10.5%. Gender differences were pronounced, with females reporting nearly double the rates of males. In Hungary, 31.3% of female adolescents and 14.9% of males reported attempts. Self-harm thoughts affected 15.0% to 43.8% of students across countries, with frequent self-harm thoughts (5+ times) ranging from 2.1% in Armenia to 15.3% in the Faroe Islands. Overall, 30.7% of students reported either self-harm thoughts, suicide attempts, or both, with higher prevalence among females (39.9%) compared to males (20.9%). |
| [105] | Kokkevi 2012 | 16 (6) | Substance use: tobacco, alcohol, unprescribed use of tranquilizers/sedatives, cannabis and other illegal drugs;  Suicide attempts: Has it ever happened that you attempted suicide? If so, how many times? | ESPAD | N = 45086, age = 15-16, self | The prevalence of any self-reported suicide attempt was 11.2 % overall and varied from 4.1 % in Armenia to 23.5 % in Hungary. In every country, the percentage of respondents who reported at least one suicide attempt increased steeply with the number of substances use |
| [106] | Koyanagi 2019 | 48 (39) | Suicide attempt: During the past 12 months, how many times did you actually attempt suicide?  Bullying victimization: During the past 30 days, on how many days were you bullied? | GSHS | N = 134229, age = 12-15, self | Bullying victimization is a strong and dose-dependent risk factor for suicide attempts among adolescents globally, especially in LMICs |
| [107] | Lai 2008 | 10 (2) | TIMSS 2003 student questionnaire | TIMSS | N = 54383, 8^th^ grade, self | The Philippines had the highest bullying rates, with over 40% of students experiencing verbal abuse, while Japan and Korea reported the lowest. Physical bullying affected 20-30% of male students in Australia, New Zealand, and Singapore. Immigrant students in Taiwan, the Philippines, Korea, and Indonesia faced higher victimization, while Hong Kong showed no such trend. Bullying negatively impacted academic performance, with students in Australia, Japan, and New Zealand reporting increased study pressure, whereas those in the Philippines, Indonesia, Malaysia, and Singapore did not experience the same effect. |
| [108] | Lambert 1998 | 2 (1) | US-Jamaican  Child Behavior Checklist (CBCL; Achenbach, 1991)- Jamaican Youth Checklist (JYC)  Teacher's Report Form (TRF; Achenbach, 1991)-JTRF  Youth Self-report (YSR; Achenbach, 1991)-JYSR | N/A | N=730, age=12-18, Self, Parents & Teachers | No significant total problem score differences were found between Jamaican and U.S. adolescents in reports by any informants. However, adolescents in both societies reported significantly more problems than their parents or teachers. Jamaican adolescents received and endorsed higher problem ratings for the Withdrawn and Somatic Complaints syndromes and on internalizing scores. U.S. adolescents obtained significantly higher scores on the cross-informant Attention Problems. Jamaican internalizing scores were significantly higher than U.S. scores for parent, teacher, and self-reports. |
| [109] | Law 2022 | 3 (2) | The School Connectedness Scale (Sieving et al., 2001);  The Cyberbullying and Cybervictimization Scale (Shapka & Law, 2013) | N/A | N = 3872, age = 16.36(China)/12.13(Canada)/15.87(Tanzanian),self | School connectedness were negatively related to cyberbullying/ victimization for all three countries, and that there were no cultural differences between being less connected to school and identifying as a cyberbully/victim. Increased school connectedness predicted lower experiences of cyberbullying/victimization. |
| [110] | Lee 2009 | 41 (14) | Math self-concept Math self-efficacy Math anxiety | PISA | N = 276165, age = 15, self | Asian countries such as Korea, and Japan, demonstrate low math self-concept and math self-efficacy and high math anxiety despite their high scores on math performance. On the other hand, some of the Western European countries such as Finland, Netherlands, Liechtenstein, and Switzerland showed a balanced outcomes, with high math performance and low levels of math anxiety. |
| [111] | Lewis 2017 | 3 (1) | The Short Mood and Feelings Questionnaire (Angold et al., 1995) | The International Youth Development Study (IYDS) | N = 7075, age = 11-18, self | The present findings revealed that adolescents in Mumbai, India, reported substantially higher depressive symptoms in both factors, but particularly for the self-critical dimension, as compared to their peers in Australia and the USA and that males in Mumbai report high levels of depressive symptoms than females in Mumbai |
| [112] | Li 2008 | 2 (1) | Revised Children’s Manifest Anxiety Scale (RCMAS) | N/A | N = 289, age = 12-17, self | Levels of anxieties did not differ based on country (China and Singapore). Gender differences were evident. Gender and grade  interaction effects were found on the anxiety scales. Mixed results were found when comparing Mainland Chinese and Singapore Chinese with the American normative sample on the different anxiety scales. |
| [113] | Li 2008 | 2 (1) | Cyberbullying experience with 15 items | N/A | N=354, age=12-15(Canada)/11-14(China), Self | Proportionally more Canadian students than Chinese students reported that they had cyberbullied others (p < 0.001) or knew someone being cyberbullied (p = 0.002). No significant differences were found in the frequencies of bullying, bully victimisation and cyberbully victimisation. Proportionally more Chinese students than Canadian students reported that adults in school tried to stop cyberbullying (p = 0.025) when notified. Similarly, Chinese cyberbullying victims (p < 0.001) and bystanders (p < 0.001) were more likely to tell adults about the incidents than their Canadian counterparts. |
| [114] | Li 2015 | 3 (2) | Rosenberg Self-Esteem Scale (RSES) Children Depression Inventory (CDI) | N/A | N = 1045, Mean age range = 8.82-14.73, self | Costa Rican adolescents scored higher on positive and negative self-esteem than their Chinese and Italian counterparts. Both positive and negative self-esteem was related to depression across three countries. |
| [115] | Li 2016 | 2 (1) | The Spence Children's Anxiety Scale (SCAS; Spence, 1997) The Strengths and Difficulties Questionnaire (SDQ; Goodman, 1997) | N/A | N = 908, age = 12-18, self | Regarding mother-report, Chinese mothers rated their children to be more anxious than did Italian mothers. Mothers considered girls to have higher anxious level than boys. No significant interaction was detected. With respect to father-report, Chinese fathers thought their children to be more anxious than did Italian fathers. Similarly, fathers also reported higher anxious level for girls than boys. In comparison to Italian mothers, Chinese mothers thought that their children had higher panic and agoraphobia, fears of physical injury, obsessive compulsive disorder and separation anxiety, but lower generalized anxiety/overanxious symptoms. Chinese fathers rated their children to have more anxiety than Italian fathers on panic and agoraphobia, fears of physical injury, obsessive-compulsive disorder and separation anxiety, but lower on generalized anxiety/overanxious symptoms. |
| [116] | Li 2019 | 2 (1) | The Strengths and Difficulties Questionnaire (SDQ; Goodman, 1997); The Meaning in Life Questionnaire (MLQ, Steger et al., 2006);  The Self-Restraint subscale from the Adolescents' Self-Consciousness Scale (Nie et al., 2014);  The Children's Depression Inventory (CDI, Kovacs, 1992) | N/A | 549, 16-17, Self | The mean (SD) of depressive symptoms was 13.13 (6.49) in China and 13.21 (7.47) in Italy. The mean (SD) of SDQ was 12.39 (5.27) in China and 13.20 (6.12) in Italy. The presence of meaning was negatively related to depressive symptoms and psychological distress in both countries. The association between presence of meaning and depressive symptoms was stronger in Italian than in Chinese adolescents. The search for meaning was positively related to depressive symptoms in both countries and psychological distress only in Italy. |
| [117] | Liu 2018 | 40 (39) | Suicide attempts: During the past 12 months, how many times did you actually attempt suicide?”. | GSHS | N = 146460, age = 12-18, self | The mean 12-month prevalence of suicide attempts was 17.2%, ranging from 6.7% in Malaysia to 61.2% in Samoa. The overall prevalence of suicide attempts was higher for girls than for boys. Among the suicide attempts, the proportion of suicide attempts with a plan was higher for girls than for boys (62.7% vs 53.2%, P<0.05). |
| [118] | Liu 2020 | 25 (25) | Depressive symptoms: “During the past 12 months, did you ever feel so sad or hopeless almost every day for two weeks or more in a row that you stopped doing your usual activities?”  Anxiety symptoms: “During the past 12 months, how often have you been so worried about something that you could not sleep at night?”. | GSHS | N = 65267, age = 12-15, self | The pooled estimates of overall prevalence rates were 30.3% for depressive symptoms and 8.3%for anxiety symptoms. The prevalence of depressive and anxiety symptoms was higher in African and Eastern Mediterranean regions compared to Americas and East and Southeast Asian regions. |
| [119] | Lukoševičiūtė 2022 | 3 (1) | Happiness: In general, how do you feel about your life at present?;  Self-rated health: Would you say your health is. . .?;  The Multiple Health Complaints scale: the frequency of eight common health symptoms in the past 6 months: headache, stomachache, backache, sleeping difficulties, feeling low, irritability or bad mood, feeling nervous, and dizziness;  Social support: Teacher/Student/Friend/Family Life satisfaction: Cantril ladder Health-related quality of life: Kidscreen-10 Index;  Well-being: WHO-5 Well-being Index; Bullying and cyberbullying; Self-directed violence (suicidal ideation, self-harming behavior) | HBSC | N = 47439, age = 11-16, Self | The prevalence of sleep difficulties ranged from 74.9% in Scotland for HICs to 81.5% in Lithuania for LMICs and 82.6% in Portugal for HICs. |
| [120] | Malykh 2013 | 2 (2) | Children`s Depression Inventory (CDI) | N/A | N = 1139, age = 7-11, self | Depressiveness decreased with age for boys in both groups, and both scored higher than their U.S. and Canadian peers. No significant differences were found between Russian and Kyrgyz girls, though Kyrgyz girls tended to score higher at younger ages (7–11 and 12–14), while Russian girls scored higher at 15–17 years, where their depressiveness significantly increased (p = .001). Kyrgyz girls showed a decrease in depressiveness at older ages. |
| [121] | Mancinelli 2021 | 4 (1) | The Strengths and Difficulties Questionnaire (SDQ; Goodman, 1997) | N/A | N=1000, age=16-17, Self | SDQ total difficulties scores varied by country, with Poland reporting the highest (14.28), followed by Italy (13.20), Spain (13.06), and China (12.39). Significant differences were found only between Poland and China, with Poland scoring 1.89 points higher on average. Gender differences were not significant. |
| [122] | Marksteiner 2020 | 47 (16) | Bullying in school; Life Satisfaction; Belonging; | PISA | N = 319057; age = 15, Self | Bullying and well-being are negatively associated. Further, the results indicated, as expected, that feelings of belonging compensate for the negative impact that bullying has on well-being. |
| [123] | McCabe 2012 | 8 (5) | Body Image and Body Change Inventory (McCabe and Ricciardelli, 2003) | N/A | N= 4641 Self | Male participants were more satisfied with their appearance except for Greek adolescents. Mean differences in body satisfaction between male and female were more significant for Australian (0.62) from HICs and Malaysian participants (0.43) from LMICs. |
| [124] | McKinnon 2016 | 32 (29) | Suicidal ideation and planning: “During the past 12 months, did you ever seriously consider attempting suicide?” and: “During the past 12 months, did you ever make a plan about how you would attempt suicide?” | GSHS | N = 164770, age 13-17, self | The pooled 12-month prevalence of suicidal ideation was higher in females (16.2%) than males (12.2%), and for ideation with a plan, it was 8.3% for females and 5.8% for males. Prevalence varied widely, from 5.1% in Indonesia to 28.1% in Zambia for ideation, and from 1.7% in Tanzania to 15.3% in Benin and Kenya for ideation with a plan. The African Region had the highest prevalence of ideation (21.6%) with no gender differences, while the Americas showed a prevalence ratio of 1.7 favoring females. The South-East Asia and Western Pacific Regions had the lowest rates, at 10.7% for ideation and 5.0% for ideation with a plan. |
| [125] | Medina 2012 | 2 (2) | Attitudes towards Suicide (ATTS);  Youth Self-report (YSR; Achenbach, 1991) | N/A | N = 684, age = 15-18, self | Cambodian adolescents scored significantly higher on most mental health syndromes compared to Nicaraguan peers, despite similar prevalence of serious suicidal expressions (plans or attempts). In Nicaragua, all YSR syndromes were strongly associated with suicidal expressions, while in Cambodia, associations were limited to specific syndromes (withdrawn/depressed for girls and somatic complaints for boys). Exposure to suicide by significant others was more common in Nicaragua (26.4%) than Cambodia (16.8%) and showed a stronger association with suicidal expressions in Nicaraguan boys. |
| [126] | Nansel 2004 | 25 (6) | Bullying/been bullied: how frequently they had been bullied at school during the cur- rent school term and how frequently they had bullied others at school during the current term? Psychosocial adjustment: health prob- lems; emotional adjustment; school ad- justment; relationship with classmates; and alcohol use;  Weapon carrying | HBSC 1997/1998 | N = 113200, age = 11.5/13.5/15.5, Self | Bullying involvement ranged from 9% in Sweden to 54% in Lithuania. Bullies, victims, and bully-victims consistently reported poorer emotional adjustment, more health problems, and worse relationships with classmates compared to noninvolved youth. Victims had the poorest emotional adjustment, while bullies exhibited more alcohol use and poorer school adjustment. Bully-victims had the worst overall psychosocial outcomes, including combined difficulties in emotional, social, and school contexts​. |
| [127] | Nguyen 2022 | 5 (3) | The Brief Symptoms Inventory (BSI) The COVID-19 impact: Please think about and assess how much the COVID-19 outbreak has personally affected you, your daily routines, work, and your family life? | N/A | N = 4670, age = 14-19, self | The association between the COVID-19 impact and psychological distress in adolescents’ lives was positive and moderate in Morocco and Serbia, positive and weak in Vietnam and the United States of America, and negative and weak in Sweden. We also found that female adolescents reported higher distress levels than male adolescents. |
| [128] | Ojala 2007 | 30 (10) | Lose weight; height and weight;  Self-perceived weight: Do you think your body is...? | HBSC survey, 2001/2002 | N= 106119 age= 14.6 (1.0)  Self | Adolescents in HICs were more dissatisfied with their bodies compared to those in LMICs. Overweight adolescents attempted weight loss more frequently than non-overweight peers across all countries. In LMICs, 5–24% of overweight boys and 28–55% of overweight girls reported weight loss attempts, compared to 3–7% of boys and 11–24% of girls who were not overweight. In HICs, weight loss attempts ranged from 10–46% among overweight boys and 28–60% among overweight girls, compared to 1–9% of boys and 9–28% of girls who were not overweight. |
| [129] | Page 2006 | 3 (2) | Attempted suicide: if had attempted suicide in the past 12 months, lifetime. Hopelessness: Beck Hopelessness Scale Loneliness: revised UCLA Loneliness Scale (R-UCLA) | N/A | N = 8463, Mean age range = 15.5-16.7, self | The prevalence of suicide attempts across the three samples of Asian youth were not consistent with Taiwanese girls and boys as the most likely to have ever attempted suicide. As expected, results showed that suicide attempters (in past 12 months and ever) scored higher on hopelessness and loneliness than non-attempters across all three samples and for both genders. |
| [130] | Page 2010 | 4 (4) | Psychological distress: “During the past 12 months, how often have you felt lonely?”, “During the past 12 months, how often have you felt worried?”, “During the past 12 months, did you ever feel so sad or hopeless almost every days for 2 weeks or more in a row that you stopped doing your usual activities?”  “During the past 12 months, did  you make a plan about how you would attempt suicide?”,  “How many close friends do you have?” | GSHS | N = 30851, age = 11-16, self | Smokers reported higher worry, sadness, and suicide plans across all groups, with loneliness differing in most but not all. In Chile, nonsmokers were likelier to lack close friends, unlike in other countries. Drug users showed more distress than non-users, except on a few indicators in Chinese, Philippine, and Namibian adolescents. |
| [131] | Page 2013 | 38 (33) | Demographics; alcohol and drug use; dietary behaviors; hygiene; mental health; physical activity; protective factors; sexual behaviors that contribute to HIV infection, other sexually transmitted infections, and to unintended pregnancy; tobacco use; and violence and unintentional injury. | GSHS | N = 266694, age = 13-15, self | Total suicide ideation prevalence at the national level ranged from a low of 0.7% in Myanmar, National (2007) to a high of 31.1% in Zambia (2003). Suicide ideation for boys ranged from 31.0% in Zambia (2003) to 0.8% in Myanmar (2007). For girls, suicide ideation ranged from 30.8% in Zambia (2003) to 0.7% in Myanmar (2007). |
| [132] | Pat-Horenczyk 2009 | 2 (1) | Objective Exposure: Palestine: a 40-item Political Violence Inventory regarding their exposure to military incursions (Haj-Yahia, 2004).  Israel: Exposure to violence, such as suicide bombings and mass shootings (Pat-Horenczyk, 2005).  Subjective Exposure: the DSM-IV-TR (American Psychiatric Association, 2000)  UCLA PTSD Reaction Index: Adolescent Version;  The Diagnostic Predictive Scales (DPS) (Lucas, Zhang, Fisher, Shaffer, Regier, Narrow, Bourdon, Dulcan, Canino, Rubio-Stipec, Lahey, & Friman, 2001); Somatic Complaints Checklist. These items were based on the DISC (Shaffer et al., 2000) Brief COPE (Carver, 1997) | N/A | N = 2251, age = 12-18, self | Conflict-related violence was linked to higher PTSD (6.8% in Israelis, 37.2% in Palestinians) and functional impairment, especially in school (61% vs. 19.9%). Girls reported more distress. Palestinians used religion and distraction to cope, while Israelis used active coping​. |
| [133] | Pfoertner 2014 | 31 (12) | Psychological health complaints psychological complaints: feeling low, bad temper or irritability, feeling nervous and difficulties falling asleep | HBSC | N(2005-6) = 164123/N(2009-10) = 168284; age=11/13/15, Self | Higher youth unemployment was linked to more psychological complaints, with a 10% increase in unemployment associated with a 5% rise in complaints. Girls (28%) and older adolescents (32%) reported higher distress than boys (18%). Countries with stronger welfare policies had lower distress rates, with Nordic countries reporting 15% fewer complaints than Eastern European countries​. |
| [134] | Pronk 2017 | 2 (1) | Bullying role behavior: Participant Roles Scales (Salmivalli et al., 1996)  Peer-group status: popularity and preference | N/A | N=699, age=13.8, Self | Indian adolescents received significantly more nominations for bully, follower and victim, and Dutch adolescents received significantly more nominations for defender. Indian boys received more nominations for victim and outsider than Indian girls and Dutch boys, while Dutch girls received more nominations for victim and outsider than Dutch boys and Indian girls. |
| [135] | Rajmil 2014 | 11 (3) | The Strengths and Difficulties Questionnaire (SDQ; Goodman, 1997);  The KIDSCREEN-10 Index | KIDSCREEN | N=10625, age=8-18, Self & Parents | Mean SDQ total difficulties scores ranged from 6.4 in the UK to 10.0 in the Czech Republic. Health-related quality of life (HRQOL) was lower among children with low Family Affluence Scale (FAS) scores, with the proportion reporting poor HRQOL ranging from 4.4% in Switzerland to 20% in Poland. |
| [136] | Ravens-Sieberer 2008 | 12 (3) | The Strengths and Difficulties Questionnaire (SDQ; Goodman, 1997);  The Family Affluence Scale (FAS, Currie, Elton, Todd, & Platt, 1997);  The Oslo Three-Item Social Support Scale (Brevik & Dalgard, 1996);  The Social Adjustment Scale (SAS, published in McDowell & Newell, 1996);  the Mental Health Components Score (MCS) from the Short Form 12 Health Survey (SF-12, Ware, Kosinski, & Keller, 1996);  The KIDSCREEN-10 Health-Related Quality of Life Questionnaire (KIDSCREEN Group Europe, 2006; Ravens-Sieberer et al., 2005) | KIDSCREEN | N=15945, age=12-18, Self | Mental health problems were highest in the UK (borderline = 13%; abnormal = 10.4%) and Hungary (borderline = 10.9%; abnormal = 7.0%) and lowest in Germany and Switzerland. Females had higher prevalence rates than males, with the largest gender gap in France and the smallest in Spain. Older adolescents in the Netherlands and Greece reported more abnormal SDQ scores. |
| [137] | Rescorla 2007 | 24 (6) | The Youth Self-Report (YSR; Achenbach & Rescorla, 2001) | N/A | N=27206, Mage=12.8-14.7, Self | Mean total problem scores ranged from 25.0 in Germany to 48.3 in Greece, with 17 of 24 countries scoring within one standard deviation of the overall mean (35.3). Adolescents consistently rated themselves as having more problems compared to parental reports, with self-reported scores averaging 34.0 versus 20.5 from parents. |
| [138] | Rodríguez-Hidalgo 2020 | 2 (1) | The European Cyberbullying Intervention Project Questionnaire (ECIP-Q); The Rosenberg Self-Esteem Scale (RSES);  The Basic Empathy Scale (BES); The Social Skills Scale | N/A | N = 24943, age = 13.92, Self | Adolescents in Spain and Ecuador showed similar cyber victimization rates, but Ecuador had higher cyberaggression; predictors like self-esteem, empathy, and social skills varied between countries. |
| [139] | Ruchkin 2005 | 2 (1) | Posttraumatic stress: The Child Post-Traumatic Stress Reaction Index; Exposure to violence during the past 2 years: Screening Survey of Exposure to Community Violence; Depression, anxiety, and somatization: Three scales from the Behavior Assessment System for Children; Expectations about the future: Four items were derived from an instrument by Jessor et al. (25) | N/A | N = 2157, age = 14-17, self | Posttraumatic stress symptoms increased with exposure to community violence in both U.S. and Russian adolescents. Higher PTSD levels were linked to greater internalizing problems, with U.S. youth reporting more exposure to violence than Russian youth. Girls exhibited PTSD symptoms at lower levels of trauma exposure than boys. Expectations for the future decreased as posttraumatic stress increased, but patterns of symptom expression were similar across both cultures. |
| [140] | Ruchkin 2006 | 3 (1) | The Social and Health Assessment Behavior Assessment;  The Behavior Assessment System for Children (BASC; Reynolds and Kamphaus, 1992)  The Social and Health Assessment scale of delinquent behavior (Schwab Stone et al., 1999);  the 10-item Disap- proval of Deviancy Scale (Jessor et al., 1989);  Perception of Risk: substance use, gun carrying, fighting, dropping out of school, sexual intercourse without a condom, etc. | N/A | N = 3309, age = 14-17, self | The prevalence of clinical levels of self-reported depression by country and gender. Students in all three countries reported similar prevalences of depressive symptoms, with the clinical symptom levels in boys ranging from 10% (in Belgium and Russia) to 13% (in the United States), and in girls from 12% (in Belgium) to 16% (in Russia) and 18% (in the United States). Within-country comparisons of depression levels between boys and girls showed significant differences in the United States (p=0.013) and in Russia (p=0.007), but not in Belgium (p=0.328). |
| [141] | Samara 2019 | 4 (1) | the Bully/Victim-Questionnaire (BVQ; Olweus, 1991) | N/A | N = 3186, age = 12-16, Self | Greek students were most likely to be involved in bullying (M = 3.08), while Palestinian students from Gaza reported the highest victimization (M = 3.23). Israeli Jewish students were more likely to be pure bullies compared to Israeli Palestinians and Gazan students, while German students had the lowest victimization scores (M = 1.34). Structural analysis found inconsistencies in how bullying was reported, with Gaza Strip data showing the least distinction between bullying and victimization, questioning cross-cultural comparability​ |
| [142] | Sentenac 2013 | 11 (3) | Self-rated health: Would you say your health is...?;  Overall life satisfaction;  Subjective health complaints: headache, stomach ache, backache, feeling low, irritability, feeling nervous, difficulties in getting to sleep, feeling dizzy) | HBSC | N = 55030, age = 11/13/15, Self | Students with disabilities or chronic illnesses face higher bullying rates globally, with LMICs showing higher prevalence; however, the health impact of bullying is similar across HICs and LMICs, highlighting the need for improved inclusive education efforts worldwide. |
| [143] | Shapka 2018 | 2 (1) | Average hours online, accessing the Internet in a private place, privacy concerns, going online for social use, and motivations for cyberbullying.  Cyberbullying and Cybervictimization: 12 items (Shapka & Law, 2013) | N/A | N = 1018, age = 11-15, Self | In Canada, girls reported higher cybervictimization, while no gender differences were found in Tanzania. More time spent online and social internet use were associated with higher cyberbullying involvement in both countries. In Tanzania, private internet access increased cyberbullying risk, while in Canada, cellphone ownership moderated cybervictimization risk. |
| [144] | Shukla 2022 | 3 (1) | Warwick Edinburgh Mental Wellbeing Scale (WEMWEBS) (Tennant et al., 2007) | N/A | N= over 616 (sample size in UK NR), Mage=15.6 (1.98), Self | Well-being scores were highest in Israel (M = 24.76) and India (M = 23.93) and lowest in the UK (M = 22.04). |
| [145] | Sitnikova 2022 | 2 (2) | Hospital Anxiety and Depression Scale (HADS) The Perceived Stress Scale (PSS) | N/A | N=1834, Mage=15.58 (1.11), Self | Kyrgyz schoolchildren reported higher levels of anxiety (7.15) and depression (5.68) in 2021 compared to Russian peers (6.54 and 5.17, respectively). The proportion of students with high depression levels decreased in Kyrgyzstan but increased in Russia over time. Girls consistently showed higher stress, anxiety, and depression than boys in both countries. Stress levels in Kyrgyz students rose from 25.88 in 2020 to 27.06 in 2021, while Russian students' stress remained stable. |
| [146] | Skinner 2022 | 4 (3) | Dyadic Coping Inventory (DCI; Bodenmann, 2008, Ledermann et al., 2010); Parental Acceptance Rejection/Control Questionnaire Short Form (Rohner, 2005);  Child Behavior Checklist (CBCL; Achenbach, 1991); Youth Self Report (YSR; Achenbach, 1991). | N/A | N = 472, age = 13-15, Self |  |
| [147] | Šmigelskas 2018 | 9 (4) | The Teacher and Classmate Support Scale; The Multidimensional Scale of Perceived Social Support (MSPSS); The Family Affluence Scale (FAS-III); Fighting: “During the past 12 months, how many times were you involved in a physical fight?” Bullying perpetration: “How often have you taken part in bullying another student(s) at school in the past couple of months?” Smoking: Monitoring the Future Study and the European School Survey Project on Alcohol and Other Drugs (ESPAD) Drunkenness: “Have you ever had so much alcohol that you were really drunk?” | HBSC 2013/2014 | N = 43667, age = 11/13/15, Self | Social support, particularly from family, significantly reduces bullying and fighting among school-aged children across European countries, with higher-income countries showing stronger associations compared to lower- and middle-income countries. |
| [148] | Springer 2007 | 2 (1) | Aggressive behavior and victimization (9 items); Depressive symptoms and suicidal ideation: 1) Felt sad or hopeless every day for 2 or more weeks in a row 2)Seriously considered attempting suicide 3) Attempted suicide;  Substance use (6 items); Sexual and reproductive behavior (3 items); Youth Risk Behavior Survey (YRBS) | N/A | N = 1856, age = 14-17, self | Latino adolescents in the U.S. had higher rates of substance use and sexual activity compared to their peers in El Salvador. Substance use prevalence was 10–40% higher in the U.S., with marijuana use being nine times greater and cocaine use five times higher. U.S. Latino adolescents were 13–27% more likely to have had sexual intercourse, but Salvadoran youth reported lower condom use (11–42% lower). |
| [149] | Stoet 2016 | 68 (22) | Mathematics anxiety: “I often worry that it will be difficult for me in mathematics classes”, “I get very tense when I have to do mathematics homework”, “I get very nervous doing mathematics problems”, “I feel helpless when doing a mathematics problem”,“I worry that I will get poor grades in mathematics”;  Excess mathematics anxiety;  Importance: “Parents believe studying mathematics is important”, “Parents believe mathematics is important for career” | PISA | N = 761655, age = 15, self, parents | Level of gender equality and direct measure of need satisfaction, the HD were negatively correlated with national averages of mathematics anxiety. In other words, the national average (i.e., combined score of boys and girls) of mathematics anxiety was lower in more gender equal and developed nations. Overall, national levels of mathematics anxiety were lower in countries with higher national performance levels in both years. |
| [150] | Sujoldzić 2007 | 3 (3) | Hopkins Symptom Checklist 25,  RADS (Reynolds Adolescent Depression Scale)  Anxiety (5 items), Depression (20 items)  SWLS (The Satisfaction With Life Scale) (5 items) | N/A | N= 1117 age= 15-18  Self | Adolescents in Kosovo reported the highest levels of body satisfaction (80% for girls, 86% for boys) and life satisfaction compared to other groups. Albanian adolescents in Albania displayed higher psychological distress, including somatic stress and anxiety, than their peers in Kosovo and Italy. Bosnian adolescents in Austria exhibited higher BMI values and self-esteem but reported lower anxiety compared to those in Bosnia and Croatia. |
| [151] | Tahmouresi 2014 | 2 (1) | Emotion Management Scale (CEMS) the Cognitive Emotion Regulation Questionnaire (CERQ) Youth Self-report (YSR; Achenbach, 1991) | N/A | N = 269, age = 11-14, self | A main result of the study showed that children in Iran report more internalizing and externalizing symptoms. Culture and emotional expression may explain differences between Iranian and German children. |
| [152] | Tang 2020 | 83 (70) | Bullying victimization: “During the past 30 days, on how many days you were bullied?”;  Suicidal ideation: “During the past 12 months, did you ever seriously consider attempting suicide?”;  Suicide planning: “During the past 12 months, did you make a plan about how you would attempt suicide?”;  Suicide attempt: “During the past 12 months, how many times did you actually attempt suicide?”  Cigarette smoking: “During the past 30 days, on how many days did you smoke cigarettes?”;  Alcohol use: “During the past 30 days, on how many days did you have at least one drink containing alcohol?”;  Close friendship: “How many close friends do you have?”;  Parental support: “During the past 30 days, how often did your parents or guardi-ns understand your problems and worries?”;  Loneliness: “During the past 12 months, how often have you felt lonely?”;  Anxiety: “During the past 12 months, how often have you been so worried about something that you could not sleep at night?”;  Socioeconomic status of each adolescent: “During the past 30 days, how often did you go hungry because there was not enough food in your home?” | GSHS | N = 220310, age = 12-15, Self | The global prevalence of suicidal ideation, planning, and attempts was approximately 16%, with being bullied affecting 35.3% of adolescents. Africa reported the highest rates of being bullied (48%) and suicidal behaviors, while Europe had the lowest rates (8.7% bullying). Girls experienced higher rates of suicidal ideation (18.2%) and planning (17.3%) compared to boys, while rates of suicide attempts were similar between genders. |
| [153] | Thorsén 2022 | 2 (1) | Perceived stress scale (PSS) 14 items; Psychiatric symptoms: 11 items | Region Skåne's Public Health Survey | N=636, age=15-16, Self | Perceived stress and psychiatric symptoms were linked in boys and girls across both countries. Swedish adolescents had higher PSS scores in the top two quartiles compared to Bulgarian peers (Swedish boys: 65%, Bulgarian boys: 24%; Swedish girls: 67%, Bulgarian girls: 41%). Girls in both countries consistently reported higher stress levels and more psychiatric and somatic symptoms than boys. In Bulgaria, girls experienced restless sleep more frequently than boys, while no gender difference was observed in Sweden. |
| [154] | Toro 2006 | 2 (1) | Eating Attitude Test-26 (EAT-26:26 items);  CETCA (Eating Disorders Assessment Questionnaire);  CIMEC (Questionnaire on Influences on Body Shape Model, 40 items);  70 Socio-demographic data, risk factors;  Attitudes towards body image (70 items). | N/A | N= 796, age= 11-18,  Self | Significant differences were found in risk behaviors: more Spanish girls reported body dissatisfaction and binging; more Mexican girls had a history of psychiatric and psychological treatment, pressure from parents and friends to lose weight, dieting, physical activity and vomiting to lose weight, and a history of greater weight loss. Mexican girls were significantly more likely to have received or be receiving psychological or psychiatric treatment and presented more cases of overweight in childhood. |
| [155] | Turner-Moore 2021 | 5 (1) | Sexual bullying questionnaire (SBQ) | ASBAE | N = 253, age = 13-18, Self | Sexual bullying victimization varied across five European countries, with the highest rates in Slovenia (100%) and the lowest in Bulgaria (68.3%). Repeat victimization was most common in Slovenia (83.3%) and least common in Bulgaria (61.7%). Female participants reported higher rates of sexual harassment, appearance-based victimization, and sexual assault than males. Italy had the highest rate of engaging in sexual bullying (100%), while Bulgaria had the lowest (45.5%). Repeat bullying was most frequent in Italy (75%) and least in England (23.3%)​ |
| [156] | Tuttle 2023 | 55 (23) | Bullying: How often during the prior 12 months they had the following experiences in school: “Other students left me out of things on purpose”; “Other students made fun of me”; “I was threatened by other students.” | PISA | N = 286871, age = 15/16, Self | Countries with higher decommodification (greater social welfare protection) had lower bullying victimization rates. On average, 9.62% of students were frequent victims of bullying, with the lowest rates in the Netherlands (2.28%) and Japan (4.32%), and the highest in the Philippines (40.13%) and the Dominican Republic (22.04%). In the U.S., the rate was slightly above average at 10.32%, emphasizing the importance of social welfare systems in reducing bullying victimization. |
| [157] | Vancampfort 2018 | 30 (30) | Depressive Symptoms: During the past 12 months, did you ever feel so sad or hopeless almost every day for two weeks or more in a row that you stopped doing your usual activities?  Sedentary behavior: “How much time do you spend during a typical or usual day sitting and watching television, playing computer games, talking with friends, or doing other sitting activities? | GSHS | N = 67077, age = 12-15, self | The prevalence of depressive symptoms was 28.7%. There was a linear increase in the prevalence of depressive symptoms with increasing sedentary time beyond ≥3 h/day (vs. < 1 h/day). Among boys, 1–2 h/day of SB was associated with lower odds for depression (vs. < 1 h/day). Countrywide meta-analysis demonstrated that spending ≥3 h/day versus < 3 h/day was associated with a 20% increased odds for depressive symptoms (OR = 1.20; 95% CI = 1.16–1.24) with low between-country heterogeneity (I2 = 27.6%). |
| [158] | Vazsonyi 2015 | 6 (2) | Big Five Weinberger Adjustment Inventory (WAI) | International Study of Adolescent Development and Problem Behaviors | N=5835, Mage=16.7, Self | Neuroticism was positively linked to all four dependent measures, while extraversion was negatively associated with depression, low well-being, and low self-esteem. Conscientiousness showed a negative relationship with low well-being and low self-esteem. Agreeableness and openness were negatively associated with low well-being and low self-esteem across all samples, with agreeableness also positively linked to anxiety. No consistent patterns emerged for agreeableness with depression or for openness with depression and anxiety. |
| [159] | Verhulst 2003 | 7 (3) | Youth Self-Report | N/A | N=3340, age=11-18, Self | Youth in LMICs generally reported higher total problems scores compared to those in HICs, with the exception of youth in Turkey, who had low scores. Across cultures, girls consistently scored higher on internalizing problems and lower on externalizing problems than boys. Mean total problems scores in LMICs included China (44.2), Jamaica (42.9), and Turkey (32.3), while in HICs they were Australia (40.2), Israel (30.4), the Netherlands (34.0), and the United States (39.3). |
| [160] | Vermeiren 2002 | 3 (1) | The Social and Health Assessment (SAHA);  the Behavioral Assessment System for Children (BASC); Expectations in the future: Four items | N/A | N = 3372, age = 14-17, self | Anxiety levels showed no consistent relationship with antisocial behavior, except in the U.S., where moderate antisocial adolescents exhibited slightly higher anxiety. Sensation-seeking was most pronounced in severe antisocial groups, particularly among Belgian boys. |
| [161] | Vittetoe 2002 | 7 (7) | Drug Use Screening Inventory (DUSI) behavioral problems: Have you teased or done harmful things to animals? Do you swear or use dirty language a lot? Do you tease others a lot? Have you threatened to hurt people? Do you do risky or dangerous things a lot? Have you taken advantage of other people? | N/A | N = 5549, age = 12-19, self | Adolescents with higher levels of behavioral problems were over five times more likely to use tobacco than those with lower levels, with country-specific differences observed. Tobacco use was highest in El Salvador (25.5%) and lowest in the Dominican Republic (3.4%). |
| [162] | Volk 2021 | 2 (1) | Bullying and victimization: bullying questionnaire (Book et al. 2012); the HEXACO Personality Inventory–Revised; Social Dominance (4 items) | N/A | N = 831, age = 14.63(China)/14.64(Canada), Self | Lower Honesty-Humility and Conscientiousness were associated with higher bullying perpetration and social dominance across Canadian and Chinese adolescents. In China, extraversion was linked to bullying and dominance, while agreeableness predicted social dominance in Canada. Bullying mediated the relationship between personality traits and dominance in both cultures. |
| [163] | Vore 2016 | 3 (1) | The Hassles Scale for Children (HSC: Parfenoff & Jose, 1989);  The Obsessive Beliefs Questionnaire (OBQ: Obsessive Compulsive Cognitions Working Group, 1997);  The Obsessive-Compulsive Inventory (OCI: Foa, Kozak, Salkovskis, Coles, & Amir, 1998) | N/A | N = 165, age 15-18, Self | Iranian participants reported lower daily stress scores (M = 11.51) than Lithuanian (M = 32.73) and British participants (M = 33.51). Obsessive-compulsive symptoms were positively correlated with beliefs, but daily stress did not significantly influence symptoms. |
| [164] | Wang 2020 | 24 (24) | Anxiety symptoms: “During the past 12 months, how often have you been so worried about something that you could not sleep at night?”  Sedentary behaviour: “How much time do you spend during a typical or usual day sitting and watching television, playing computer games, talking with friends, or doing other sitting activities?” | GSHS | N = 59587, age = 12-15, self | Anxiety symptoms were reported by 10.3% of adolescents, with the highest prevalence in Africa (13.1%) and the lowest in East and Southeast Asia (5.2%). Sedentary behavior exceeding 2 hours per day was linked to higher anxiety, with the strongest effects in Seychelles and Indonesia. |
| [165] | Weine 1995 | 2 (1) | Child Behavior Checklist (CBCL; Achenbach, 1991) Teacher reported Teacher's Report Form (TRF) (Achenbach, 1991) | N/A | N=938. age=6-13, Parents & Teachers | Chinese children had higher scores on teacher-reported delinquent behavior, anxious-depressed symptoms, and internalizing problems, while American children scored higher on parent-reported aggressive behavior and attention problems. |
| [166] | Weitkamp 2019 | 8 (4) | The Youth Self Report (YSR; Achenbach, 1991); the Adolescent Family Process measure (AFP; Vazsonyi et al. 2003); | N/A | N = 2415, age = 14-16, self | Parental psychological control was linked to higher adolescent internalizing symptoms across all eight countries. Maternal anxious rearing was a strong predictor of both internalizing and externalizing problems, particularly in Pakistan (M = 3.45) and Turkey (M = 3.04). Paternal support was associated with lower internalizing symptoms, but paternal psychological control was linked to increased externalizing behaviors, especially in Germany (M = 3.05). Adolescents in Peru and Argentina reported the highest levels of maternal support (M = 4.27, M = 4.18), while those in Pakistan had the highest perceived parental psychological control (M = 2.95)​. |
| [167] | Yuan 2023 | 5 (3) | M-anxiety: I often worry that it will be difficult for me in mathematics classes; I am just not good at mathematics; I get very tense when I have to do mathematics homework; I get good grades in mathematics; I get very nervous doing mathematics problems; I learn mathematics quickly; I have always believed that mathematics is one of my best subjects ;I feel helpless when doing a mathematics problem; In my mathematics class, I understand even the most difficult work; I worry that I will get poor grades in mathematics | PISA | N = 17284, age = 15, self | Mathematics anxiety was negatively associated with standardized test scores and math interest across all countries. In Shanghai and Singapore, students who rated their abilities highly and valued math performed better, while in Malaysia and Indonesia, these relationships were reversed. Parent and peer attitudes influenced math anxiety in Malaysia, Indonesia, and Korea but had no significant impact in Shanghai and Singapore. |
| [168] | Zgambo 2014 | 2 (2) | Children Depression Inventory (CDI) | N/A | N =478, age = 8-12, self | Chinese children had a higher prevalence of clinically significant depressive symptoms (16%) compared to Malawian children (12.4%). Among Chinese children, sibling conflict (aOR = 4.1), parent-child conflict (aOR = 7.7), and living with only the father (aOR = 4.1) were significant predictors of depressive symptoms. For Malawian children, having only the mother employed increased the risk (aOR = 3.0). Chinese children scored higher on negative self-esteem, while Malawian children had higher anhedonia. |
| [169] | Zhang 2023 | 2 (1) | The Strength and Difficulties Questionnaire (SDQ);  Body image and eating distress scale 9 items; Bullying 3 items; school safety 4 items; | ECAMHS | N=1840, Mage=13.9 (SD:0.3) in Japan/14.6 (SD:1.3) in Russia, Self | Japanese adolescents reported more mental health problems and lower school safety than Russian adolescents. Girls had higher mental health issues, less bullying victimization, and more body dissatisfaction than boys. School safety had a stronger protective effect for girls, while bullying victimization had a greater negative impact on Japanese girls. |
| [170] | Zhao 2019 | 2 (1) | Math anxiety: 5 items (e.g., worry that it will be difficult, get very tense, and  feel helpless) | PISA | N = 10471, age = 15-16, self | Math self-efficacy positively influenced math knowledge in both the U.S. and China. In the U.S., math self-concept improved math knowledge (β = 0.038), while math anxiety (β = -0.047) and perceived peer math norms (β = -0.033) had negative effects. In China, positive student-teacher relationships (β = 0.024) and lower student-teacher ratios (β = 0.027) were linked to better math knowledge, while a stronger sense of belonging had a negative effect (β = -0.040). |
| [171] | Zhou 2009 | 2 (2) | Child Behavior Checklist (CBCL; Achenbach, 1991)  China - Child Behavior US - EATQ  Both - CBCL (parent/teacher) for internalizing and externalizing (different version) | N/A | N = 704, age = 10-13, self & parents/caregiver | Lower effortful control and higher anger-irritability were linked to more externalizing problems, with stronger effects in China. Positive emotionality reduced internalizing problems in both countries but increased externalizing behaviors in China. Chinese parents rated boys higher on internalizing problems, unlike U.S. parents. Socioeconomic status was positively linked to effortful control and negatively to adjustment problems​. |
| [172] | Zietz 2022 | 7 (5) | Youth Self Report | N/A | N = 1082, age = 12-15, self & parents | Economic pressure was lowest in European American families (12.6%) compared to the overall sample average (39%). In Colombia, income loss at age 12 was linked to increased externalizing behaviors at age 15 (6.04), while in Rome, maternal depression was negatively associated with externalizing behaviors (-1.95). |

*abbrevasions:HBSC the Health Behaviour in School-aged Children; GSHS: Global School-based Health Survey; PISA: Program for International Student Assessment; ECAMHS: the Eurasian Child and Adolescent Mental Health Study; ASBAE: The Addressing Sexual Bullying Across Europe; KIDSCREEN: the European Screening for and Promotion of Health-Related Quality of Life in Children and Adolescents—A European Public Health Perspective; TIMSS: Third International Mathematics and Science Study; GEAS: The Global Early Adolescent Study; ESPAD: The European School Survey Project on Alcohol and Other Drugs.

*The number of countries/territories was extracted from the original study using the World Bank classification to categorize High-Income Countries (HICs) and Low- and Middle-Income Countries (LMICs) based on the data collection year.

**References**

1. Abio A, Owusu PN, Posti JP, et al (2022) Cross-national examination of adolescent suicidal behavior: a pooled and multi-level analysis of 193,484 students from 53 LMIC countries. Soc Psychiatry Psychiatr Epidemiol 57:1603–1613. https://doi.org/10.1007/S00127-022-02287-X

2. Al Sabbah H, Vereecken CA, Elgar FJ, et al (2009) Body weight dissatisfaction and communication with parents among adolescents in 24 countries: International cross-sectional survey. BMC Public Health 9:. https://doi.org/10.1186/1471-2458-9-52

3. Arat G, Wong PWC (2017) The relationship between physical activity and mental health among adolescents in six middle-income countries: A cross-sectional study. Child Youth Serv 38:180–195. https://doi.org/10.1080/0145935X.2017.1297202

4. Assarsson R, Petersen S, Högberg B, et al (2018) Gender inequality and adolescent suicide ideation across Africa, Asia, the South Pacific and Latin America–a cross-sectional study based on the Global School Health Survey (GSHS). Glob Health Action 11:. https://doi.org/10.1080/16549716.2019.1663619

5. Athanasiou K, Melegkovits E, Andrie EK, et al (2018) Cross-national aspects of cyberbullying victimization among 14-17-year-old adolescents across seven European countries. BMC Public Health 18:. https://doi.org/10.1186/S12889-018-5682-4

6. Auerbach RP, Abela JRZ, Zhu X, Yao S (2010) Understanding the role of coping in the development of depressive symptoms: Symptom specificity, gender differences, and cross-cultural applicability. British Journal of Clinical Psychology 49:547–561. https://doi.org/10.1348/014466509X479681

7. Auerbach AL, Auerbach RP, Webb CA, et al (2011) Intrinsic and Extrinsic Aspirations exaMining the pathWay through Which intrinsic and extrinsic aspirations generate stress and subsequent depressive syMptoMs

8. Badura P, Hamrik Z, DIerckens M, et al (2021) After the bell: adolescents’ organised leisure-time activities and well-being in the context of social and socioeconomic inequalities. J Epidemiol Community Health 75:628–636. https://doi.org/10.1136/JECH-2020-215319

9. Bagley C, Mallick K, Verma G, et al (1999) Adjustment, stress and family life in adolescents in Canada, Britain, Hong Kong, India, Pakistan and The Philippines. Int J Adolesc Youth 7:263–278. https://doi.org/10.1080/02673843.1999.9747830

10. Baird S, Bhutta ZA, Hamad BA, et al (2019) Do restrictive gender attitudes and norms influence physical and mental health during very young Adolescence?Evidence from Bangladesh and Ethiopia. SSM Popul Health 9:. https://doi.org/10.1016/j.ssmph.2019.100480

11. Balogun O, Koyanagi A, Stickley A, et al (2014) Alcohol consumption and psychological distress in adolescents: A multi-country study. Journal of Adolescent Health 54:228–234. https://doi.org/10.1016/j.jadohealth.2013.07.034

12. Beckwith S, Lou C, Michielsen K, et al (2022) Violence Perpetration in Early Adolescence: A Study of Four Urban Communities Worldwide. Journal of Adolescent Health 71:616–627. https://doi.org/10.1016/j.jadohealth.2022.06.011

13. Biswas T, Thomas HJ, Scott JG, et al (2022) Variation in the prevalence of different forms of bullying victimisation among adolescents and their associations with family, peer and school connectedness: a population-based study in 40 lower and middle income to high-income countries (LMIC-HICs). J Child Adolesc Trauma 15:1029–1039. https://doi.org/10.1007/S40653-022-00451-8/TABLES/2

14. Bochaver AA, Navarro-Rodríguez CD, Korneev A, et al (2022) A comparative study of youth victimization during COVID-19 lockdowns in Mexico and Russia. Psychology, Society and Education 14:38–47. https://doi.org/10.21071/PSYE.V14I3.15047

15. Bravo-Sanzana M, Oriol X, Miranda R (2022) Characterization of Wellbeing and its Relationship with Exposure to Violence in Mexican and Chilean Early and Late Adolescents during the COVID-19 Pandemic. Child Indic Res 15:553–578. https://doi.org/10.1007/s12187-021-09905-1

16. Bravo-Sanzana M, Miranda R, Oriol X (2023) Adolescent Victimization during COVID-19 Lockdowns and Its Influence on Mental Health Problems in Seven Countries: The Mediation Effect of Resilience. Int J Environ Res Public Health 20:. https://doi.org/10.3390/ijerph20031958

17. Brown DW, Riley L, Butchart A, Kann L (2008) Bullying among youth from eight African countries and associations with adverse health behaviors. Ped Health 2:289–299. https://doi.org/10.2217/17455111.2.3.289

18. Buist KL, Verhoeven M, Hoksbergen R, et al (2017) Associations of Perceived Sibling and Parent-Child Relationship Quality With Internalizing and Externalizing Problems: Comparing Indian and Dutch Early Adolescents. Journal of Early Adolescence 37:1163–1190. https://doi.org/10.1177/0272431616653473

19. Calmaestra J, Rodríguez-Hidalgo AJ, Mero-Delgado O, Solera E (2020) Cyberbullying in adolescents from Ecuador and Spain: Prevalence and differences in gender, school year and ethnic-cultural background. Sustainability (Switzerland) 12:. https://doi.org/10.3390/su12114597

20. Campbell OLK, Bann D, Patalay P (2021) The gender gap in adolescent mental health: A cross-national investigation of 566,829 adolescents across 73 countries. SSM Popul Health 13:. https://doi.org/10.1016/J.SSMPH.2021.100742

21. Chen C, Greenberger E, Lester J, et al (1998) A cross-cultural study of family and peer correlates of adolescent misconduct. Dev Psychol 34:770–781. https://doi.org/10.1037/0012-1649.34.4.770

22. Chen X, He Y, De Oliveira AM, et al (2004) Loneliness and social adaptation in Brazilian, Canadian, Chinese and Italian children: A multi-national comparative study. J Child Psychol Psychiatry 45:1373–1384. https://doi.org/10.1111/j.1469-7610.2004.00329.x

23. Chen JK, Chen LM (2020) A Cross-National Examination of School Violence and Nonattendance Due to School Violence in Taiwan, Hong Kong, and Mainland China: A Rasch Model Approach. J Sch Violence 19:177–191. https://doi.org/10.1080/15388220.2019.1568882

24. Chen JK, Chen LM (2020) Cyberbullying among adolescents in Taiwan, Hong Kong, and Mainland China: a cross-national study in Chinese societies. Asia Pac J Soc Work Dev 227–241. https://doi.org/10.1080/02185385.2020.1788978

25. Chen JK (2020) Cyber victimisation, social support, and psychological distress among junior high school students in Taiwan and Mainland China. Asia Pac J Soc Work Dev 30:150–163. https://doi.org/10.1080/02185385.2020.1755994

26. Chen JK, Wang SC, Chen YW (2023) Social Relationships as Mediators of Material Deprivation, School Bullying Victimization, and Subjective Well-Being among Children Across 25 Countries: A Global and Cross-National Perspective. Appl Res Qual Life 18:2415–2440. https://doi.org/10.1007/s11482-023-10192-x

27. Chester KL, Callaghan M, Cosma A, et al (2015) Cross-national time trends in bullying victimization in 33 countries among children aged 11, 13 and 15 from 2002 to 2010. Eur J Public Health 25:61–64. https://doi.org/10.1093/EURPUB/CKV029

28. Chudal R, Tiiri E, Brunstein Klomek A, et al (2022) Victimization by traditional bullying and cyberbullying and the combination of these among adolescents in 13 European and Asian countries. Eur Child Adolesc Psychiatry 31:1391–1404. https://doi.org/10.1007/S00787-021-01779-6

29. Cosma A, Walsh SD, Chester KL, et al (2020) Bullying victimization: time trends and the overlap between traditional and cyberbullying across countries in Europe and North America. Int J Public Health 65:75–85. https://doi.org/10.1007/s00038-019-01320-2

30. Cosma A, Költő A, Chzhen Y, et al (2022) Measurement Invariance of the WHO-5 Well-Being Index: Evidence from 15 European Countries. Int J Environ Res Public Health 19:9798. https://doi.org/10.3390/IJERPH19169798/S1

31. Cosma A, Bjereld Y, Elgar FJ, et al (2022) Gender Differences in Bullying Reflect Societal Gender Inequality: A Multilevel Study With Adolescents in 46 Countries. Journal of Adolescent Health 71:601–608. https://doi.org/10.1016/j.jadohealth.2022.05.015

32. Craig W, Harel-Fisch Y, Fogel-Grinvald H, et al (2009) A cross-national profile of bullying and victimization among adolescents in 40 countries. Int J Public Health 54:. https://doi.org/10.1007/S00038-009-5413-9

33. Craig W, Boniel-Nissim M, King N, et al (2020) Social Media Use and Cyber-Bullying: A Cross-National Analysis of Young People in 42 Countries. Journal of Adolescent Health 66:S100–S108. https://doi.org/10.1016/J.JADOHEALTH.2020.03.006

34. Crocetti E, Hale WW, Dimitrova R, et al (2015) Generalized Anxiety Symptoms and Identity Processes in Cross-Cultural Samples of Adolescents from the General Population. Child Youth Care Forum 44:159–174. https://doi.org/10.1007/s10566-014-9275-9

35. Crous G (2017) Child psychological well-being and its associations with material deprivation and type of home. Child Youth Serv Rev 80:88–95. https://doi.org/10.1016/J.CHILDYOUTH.2017.06.051

36. Delvecchio E, Mabilia D, Di Riso D, et al (2015) A Comparison of Anxiety Symptoms in Community-Based Chinese and Italian Adolescents. J Child Fam Stud 24:2418–2431. https://doi.org/10.1007/s10826-014-0045-y

37. Deryol R, Wilcox P, Stone S (2022) Individual Risk, Country-Level Social Support, and Bullying and Cyberbullying Victimization Among Youths: A Cross-national Study. https://doi.org/101177/08862605211015226 37:NP15275–NP15311. https://doi.org/10.1177/08862605211015226

38. Di Giunta L, Iselin AMR, Lansford JE, et al (2018) Parents’ and early adolescents’ self-efficacy about anger regulation and early adolescents’ internalizing and externalizing problems: A longitudinal study in three countries. J Adolesc 64:124–135. https://doi.org/10.1016/j.adolescence.2018.01.009

39. Di Giunta L, Rothenberg WA, Lunetti C, et al (2020) Longitudinal associations between mothers’ and fathers’ anger/irritability expressiveness, harsh parenting, and adolescents’ socioemotional functioning in nine countries. Dev Psychol 56:458–474. https://doi.org/10.1037/dev0000849

40. Di Giunta L, Lunetti C, Lansford JE, et al (2023) Predictors and outcomes associated with the growth curves of self-efficacy beliefs in regard to anger and sadness regulation during adolescence: a longitudinal cross-cultural study. Front Psychol 14:. https://doi.org/10.3389/fpsyg.2023.1010358

41. Dmitrieva J, Chen C, Greenberger E, Gil-Rivas V (2004) Family relationships and adolescent psychosocial outcomes: Converging findings from eastern and western cultures. Journal of Research on Adolescence 14:425–447

42. Doty JL, Mehari KR, Sharma D, et al (2023) Cross-Cultural Measurement of Cyberbullying Perpetration and Victimization in India and the U.S. J Psychopathol Behav Assess 45:1068–1080. https://doi.org/10.1007/s10862-023-10039-7

43. Due P, Holstein BE, Lynch J, et al (2005) Bullying and symptoms among school-aged children: international comparative cross sectional study in 28 countries. Eur J Public Health 15:128–132. https://doi.org/10.1093/EURPUB/CKI105

44. Due P, Holstein BE, Soc MS (2008) Bullying victimization among 13 to 15-year-old school children: results from two comparative studies in 66 countries and regions. Int J Adolesc Med Health 20:209–221. https://doi.org/10.1515/IJAMH.2008.20.2.209

45. Due P, Merlo J, Harel-Fisch Y, et al (2009) Socioeconomic inequality in exposure to bullying during adolescence: A comparative, cross-sectional, multilevel study in 35 countries. Am J Public Health 99:907–914. https://doi.org/10.2105/AJPH.2008.139303

46. Duinhof EL, Lek KM, De Looze ME, et al (2020) Revising the self-report strengths and difficulties questionnaire for cross-country comparisons of adolescent mental health problems: the SDQ-R. Epidemiol Psychiatr Sci 29:e35. https://doi.org/10.1017/S2045796019000246

47. Dzielska A, Kelly C, Ojala K, et al (2020) Weight Reduction Behaviors Among European Adolescents—Changes From 2001/2002 to 2017/2018. Journal of Adolescent Health 66:S70–S80. https://doi.org/10.1016/j.jadohealth.2020.03.008

48. Elgar FJ, Craig W, Boyce W, et al (2009) Income inequality and school bullying: multilevel study of adolescents in 37 countries. J Adolesc Health 45:351–359. https://doi.org/10.1016/J.JADOHEALTH.2009.04.004

49. Elgar FJ, Pförtner TK, Moor I, et al (2015) Socioeconomic inequalities in adolescent health 2002–2010: a time-series analysis of 34 countries participating in the Health Behaviour in School-aged Children study. The Lancet 385:2088–2095. https://doi.org/10.1016/S0140-6736(14)61460-4

50. Erskine HE, Maravilla JC, Wado YD, et al (2024) Prevalence of adolescent mental disorders in Kenya, Indonesia, and Viet Nam measured by the National Adolescent Mental Health Surveys (NAMHS): a multi-national cross-sectional study. The Lancet 403:1671–1680. https://doi.org/10.1016/S0140-6736(23)02641-7

51. Eskin M (1995) Suicidal behavior as related to social support and assertiveness among Swedish and Turkish high school students: A cross‐cultural investigation. J Clin Psychol 51:158–172. https://doi.org/10.1002/1097-4679(199503)51:2<158::AID-JCLP2270510204>3.0.CO;2-H

52. Eskin M, Palova E, Krokavcova M (2014) Suicidal Behavior and Attitudes in Slovak and Turkish High School Students: A Cross-Cultural Investigation. Archives of Suicide Research 18:58–73. https://doi.org/10.1080/13811118.2013.803448

53. Eslea M, Menesini E, Morita Y, et al (2004) Friendship and loneliness among bullies and victims: Data from seven countries. Aggress Behav 30:71–83. https://doi.org/10.1002/ab.20006

54. Farruggia SP, Chen C, Greenberger E, et al (2004) Adolescent self-esteem in cross-cultural perspective: Testing measurement equivalence and a mediation model. J Cross Cult Psychol 35:719–733. https://doi.org/10.1177/0022022104270114

55. Fine SL, Musci RJ, Bass JK, et al (2022) A Multi-Country Study of Risk and Protective Factors for Emotional and Behavioral Problems Among Early Adolescents. Journal of Adolescent Health 71:480–487. https://doi.org/10.1016/J.JADOHEALTH.2022.05.002

56. Fine SL, Blum RW, Bass JK, et al (2023) A latent class approach to understanding patterns of emotional and behavioral problems among early adolescents across four low- and middle-income countries. Dev Psychopathol 35:1684–1700. https://doi.org/10.1017/S0954579422000384

57. Fismen AS, Galler M, Klepp KI, et al (2022) Weight Status and Mental Well-Being Among Adolescents: The Mediating Role of Self-Perceived Body Weight. A Cross-National Survey. Journal of Adolescent Health 71:187–195. https://doi.org/10.1016/J.JADOHEALTH.2022.02.010

58. Fleming LC, Jacobsen KH (2010) Bullying among middle-school students in low and middle income countries. Health Promot Int 25:73–84. https://doi.org/10.1093/heapro/dap046

59. Frenzel AC, Thrash TM, Pekrun R, Goetz T (2007) Achievement emotions in Germany and China: A cross-cultural validation of the academic emotions questionnaire-mathematics. J Cross Cult Psychol 38:302–309. https://doi.org/10.1177/0022022107300276

60. Germani A, Delvecchio E, Li J Bin, et al (2021) Meaning in Life as Mediator of Family Allocentrism and Depressive Symptoms Among Chinese and Italian Early Adolescents. Youth Soc 53:252–272. https://doi.org/10.1177/0044118X20921637

61. Ghekiere A, Van Cauwenberg J, Vandendriessche A, et al (2019) Trends in sleeping difficulties among European adolescents: Are these associated with physical inactivity and excessive screen time? Int J Public Health 64:487–498. https://doi.org/10.1007/S00038-018-1188-1

62. Gillé V, Kerkhoff D, Heim-Dreger U, et al (2021) Stress-symptoms and well-being in children and adolescents: factor structure, measurement invariance, and validity of English, French, German, Russian, Spanish, and Ukrainian language versions of the SSKJ scales. Health Psychol Behav Med 9:875–894. https://doi.org/10.1080/21642850.2021.1990062

63. Gobina I, Zaborskis A, Pudule I, et al (2008) Bullying and subjective health among adolescents at schools in Latvia and Lithuania. Int J Public Health 53:272–276. https://doi.org/10.1007/S00038-008-7041-1/METRICS

64. Gomez-Baya D, Babić Čikeš A, Hirnstein M, et al (2022) Positive Youth Development and Depression: An Examination of Gender Differences in Croatia and Spain. Front Psychol 12:. https://doi.org/10.3389/FPSYG.2021.689354

65. Görzig A, Milosevic T, Staksrud E (2017) Cyberbullying Victimization in Context: The Role of Social Inequalities in Countries and Regions. J Cross Cult Psychol 48:1198–1215. https://doi.org/10.1177/0022022116686186

66. Govorova E, Benítez I, Muñiz J (2020) How Schools Affect Student Well-Being: A Cross-Cultural Approach in 35 OECD Countries. Front Psychol 11:. https://doi.org/10.3389/fpsyg.2020.00431

67. Greenberger E, Chena C, Tallya SR, Dong Q (2000) Family, peer, and individual correlates of depressive symptomatology among U.S. and Chinese adolescents. J Consult Clin Psychol 68:209–219. https://doi.org/10.1037/0022-006X.68.2.209

68. Greenberger E, Chen C, Beam M, et al (2000) The perceived social contexts of adolescents’ misconduct: A comparative study of youths in three cultures. Journal of Research on Adolescence 10:365–388

69. Gross-Manos D, Bradshaw J (2022) The Association Between the Material Well-Being and the Subjective Well-Being of Children in 35 Countries. Child Indic Res 15:. https://doi.org/10.1007/S12187-021-09860-X

70. Güngör D, Bornstein MH (2010) Culture-general and -specific associations of attachment avoidance and anxiety with perceived parental warmth and psychological control among Turk and Belgian adolescents. J Adolesc 33:593–602. https://doi.org/10.1016/j.adolescence.2009.12.005

71. Gupta T, Way N, McGill RK, et al (2013) Gender-Typed Behaviors in Friendships and Well-Being: A Cross-Cultural Study of Chinese and American Boys. Journal of Research on Adolescence 23:57–68. https://doi.org/10.1111/j.1532-7795.2012.00824.x

72. Haid ML, Seiffge-Krenke I, Molinar R, et al (2010) Identity and future concerns among adolescents from Italy, Turkey and Germany: Intra- and between-cultural comparisons. J Youth Stud 13:369–389. https://doi.org/10.1080/13676260903447528

73. Han Y, Kang HR, Choe JW, Kim H (2021) The moderating role of parental support in the relationship between latent profiles of bullying victimization and sense of school belonging: A cross-national comparison. Child Youth Serv Rev 122:. https://doi.org/10.1016/j.childyouth.2020.105827

74. Harel-Fisch Y, Radwan Q, Walsh SD, et al (2010) Psychosocial outcomes related to subjective threat from armed conflict events (STACE): Findings from the Israeli-Palestinian cross-cultural HBSC study. Child Abuse Negl 34:623–638. https://doi.org/10.1016/J.CHIABU.2009.12.007

75. Harel-Fisch Y, Walsh SD, Fogel-Grinvald H, et al (2011) Negative school perceptions and involvement in school bullying: A universal relationship across 40 countries. J Adolesc 34:639–652. https://doi.org/10.1016/J.ADOLESCENCE.2010.09.008

76. Harel-Fisch Y, Abdeen Z, Walsh SD, et al (2012) Multiple risk behaviors and suicidal ideation and behavior among Israeli and Palestinian adolescents. Soc Sci Med 75:98–108. https://doi.org/10.1016/J.SOCSCIMED.2012.03.005

77. Heinz A, Catunda C, van Duin C, et al (2020) Patterns of Health-Related Gender Inequalities—A Cluster Analysis of 45 Countries. Journal of Adolescent Health 66:S29–S39. https://doi.org/10.1016/j.jadohealth.2020.02.011

78. Hillekens J, Buist KL, Horváth LO, et al (2020) Parent-early adolescent relationship quality and problem behavior in Hungary, the Netherlands, India, and Iceland. Scand J Psychol 61:763–774. https://doi.org/10.1111/sjop.12667

79. Högberg B (2021) Educational stressors and secular trends in school stress and mental health problems in adolescents. Soc Sci Med 270:113616. https://doi.org/10.1016/J.SOCSCIMED.2020.113616

80. Hosozawa M, Bann D, Fink E, et al (2021) Bullying victimisation in adolescence: prevalence and inequalities by gender, socioeconomic status and academic performance across 71 countries. EClinicalMedicine 41:. https://doi.org/10.1016/j.eclinm.2021.101142

81. Hussein MH (2010) The Peer Interaction in Primary School Questionnaire: Testing for measurement equivalence and latent mean differences in bullying between gender in Egypt, Saudi Arabia and the USA. Social Psychology of Education 13:57–76. https://doi.org/10.1007/s11218-009-9098-y

82. Imran S, MacBeth A, Quayle E, Chan SWY (2021) Secondary attachment and mental health in Pakistani and Scottish adolescents: A moderated mediation model. Psychology and Psychotherapy: Theory, Research and Practice 94:339–358. https://doi.org/10.1111/papt.12280

83. Isaksson J, Isaksson M, Stickley A, et al (2023) Community Violence Exposure and Eating Disorder Symptoms among Belgian, Russian and US Adolescents: Cross-Country and Gender Perspectives. Child Psychiatry Hum Dev. https://doi.org/10.1007/S10578-023-01590-1

84. Iwawaki S, Sarmany-Schuller I (2001) Cross-cultural (Japan-Slovakia) comparison of some aspects of sleeping patterns and anxiety. Stud Psychol (Bratisl) 43:215–224

85. Jacob L, Stubbs B, Firth J, et al (2020) Fast food consumption and suicide attempts among adolescents aged 12–15 years from 32 countries. J Affect Disord 266:63–70. https://doi.org/10.1016/j.jad.2020.01.130

86. Jacob L, Stubbs B, Koyanagi A (2020) Consumption of carbonated soft drinks and suicide attempts among 105,061 adolescents aged 12–15 years from 6 high-income, 22 middle-income, and 4 low-income countries. Clin Nutr 39:886–892. https://doi.org/10.1016/j.clnu.2019.03.028

87. Jessor R, Turbin MS, Costa FM, et al (2003) Adolescent Problem Behavior in China and the United States: A Cross-National Study of Psychosocial Protective Factors. Journal of research on adolescence 13:329–360. https://doi.org/10.1111/1532-7795.1303004

88. G Johnson R, Allen KA, Gallo Cordoba B (2024) Where does culture belong at school? Exploring the role of individualism and power distance in school belonging across cultures. Current Psychology 43:13492–13527. https://doi.org/10.1007/s12144-023-05280-y

89. Kahumoku EP, Vazsonyi AT, Pagava K, et al (2011) Objectified body consciousness and mental health in female adolescents: Cross-cultural evidence from Georgian and Swiss national samples. Journal of Adolescent Health 49:141–147. https://doi.org/10.1016/j.jadohealth.2010.11.001

90. Kakar V, Fardouly J, Rapee RM, et al (2023) Appearance Satisfaction Among Adolescent Girls in Australia, China, India, and Iran: The Role of Perceived Actual-Ideal Discrepancies in Facial and Bodily Attributes. Sex Roles 89:257–276. https://doi.org/10.1007/s11199-023-01395-5

91. Kakar V, Fardouly J, Rapee RM, et al (2023) Exploring the tripartite influence model of body image and disordered eating among adolescent girls living in Australia, China, India, and Iran. Body Image 47:. https://doi.org/10.1016/j.bodyim.2023.101633

92. Kalaycıoğlu DB (2015) The influence of socioeconomic status, self-efficacy, and anxiety on mathematics achievement in England, Greece, Hong Kong, the Netherlands, Turkey, and the USA. Kuram ve Uygulamada Egitim Bilimleri 15:1391–1401. https://doi.org/10.12738/estp.2015.5.2731

93. Kapetanovic S, Rothenberg WA, Lansford JE, et al (2020) Cross-Cultural Examination of Links between Parent–Adolescent Communication and Adolescent Psychological Problems in 12 Cultural Groups. J Youth Adolesc 49:1225–1244. https://doi.org/10.1007/s10964-020-01212-2

94. Katsantonis IG Cultural Variation in Aggressive Behavior: A Cross-Cultural Comparison of Students’ Exposure to Bullying Across 32 Countries

95. Kayano M, Yoshiuchi K, Al-Adawi S, et al (2008) Eating attitudes and body dissatisfaction in adolescents: Cross-cultural study. Psychiatry Clin Neurosci 62:17–25. https://doi.org/10.1111/j.1440-1819.2007.01772.x

96. Kearns JC, Kittel JA, Schlagbaum P, et al (2022) Worry-related sleep problems and suicidal thoughts and behaviors among adolescents in 88 low-, middle-, and high-income countries: an examination of individual- and country-level factors. Eur Child Adolesc Psychiatry 31:1995–2011. https://doi.org/10.1007/s00787-021-01838-y

97. Khan A, Lee EY, Rosenbaum S, et al (2021) Dose-dependent and joint associations between screen time, physical activity, and mental wellbeing in adolescents: an international observational study. Lancet Child Adolesc Health 5:729–738. https://doi.org/10.1016/S2352-4642(21)00200-5

98. Khan A, Lee EY, Horwood S (2022) Adolescent screen time: associations with school stress and school satisfaction across 38 countries. Eur J Pediatr 181:2273–2281. https://doi.org/10.1007/s00431-022-04420-z

99. Kim S, Spadafora N, Craig W, et al (2021) Disciplinary Structure and Teacher Support in Chinese and Canadian Schools: Examining How Authoritative Disciplinary Practices Protect Youth Involved in Bullying at School. School Ment Health 13:501–517. https://doi.org/10.1007/s12310-021-09431-z

100. Kim SS, Craig WM, King N, et al (2022) Bullying, Mental Health, and the Moderating Role of Supportive Adults: A Cross-National Analysis of Adolescents in 45 Countries. Int J Public Health 67:. https://doi.org/10.3389/IJPH.2022.1604264

101. King RB, Cai Y, Elliot AJ (2024) Income inequality is associated with heightened test anxiety and lower academic achievement: A cross-national study in 51 countries. Learn Instr 89:. https://doi.org/10.1016/j.learninstruc.2023.101825

102. Klinger DA, Freeman JG, Bilz L, et al (2015) Cross-national trends in perceived school pressure by gender and age from 1994 to 2010. Eur J Public Health 25:51–56. https://doi.org/10.1093/EURPUB/CKV027

103. Koenig LR, Blum RW, Shervington D, et al (2021) Unequal Gender Norms Are Related to Symptoms of Depression Among Young Adolescents: A Cross-Sectional, Cross-Cultural Study. Journal of Adolescent Health 69:S47–S55. https://doi.org/10.1016/j.jadohealth.2021.01.023

104. Kokkevi A, Rotsika V, Arapaki A, Richardson C (2012) Adolescents’ self-reported suicide attempts, self-harm thoughts and their correlates across 17 European countries. Journal of child psychology and psychiatry 53:381–389. https://doi.org/10.1111/j.1469-7610.2011.02457.x

105. Kokkevi A, Richardson C, Olszewski D, et al (2012) Multiple substance use and self-reported suicide attempts by adolescents in 16 European countries. Eur Child Adolesc Psychiatry 21:443–450. https://doi.org/10.1007/s00787-012-0276-7

106. Koyanagi A, Oh H, Carvalho AF, et al (2019) Bullying Victimization and Suicide Attempt Among Adolescents Aged 12–15 Years From 48 Countries. J Am Acad Child Adolesc Psychiatry 58:907-918.e4. https://doi.org/10.1016/j.jaac.2018.10.018

107. Lai S-L, Ye R, Chang K-P (2008) Bullying in Middle Schools: An Asian-Pacifit Regional Study. 9:503

108. Lambert MC, Lyubansky M, Achenbach TM (1998) Behavioral and Emotional Problems Among Adolescents of Jamaica and the United States: Parent,Teacher, and Self-Reports for Ages 12 to 18. 180–187

109. Law DM, Xiao B, Onditi H, et al (2022) Measurement Invariance and Relationships Among School Connectedness, Cyberbullying, and Cybervictimization: A Comparison Among Canadian, Chinese, and Tanzanian Adolescents. J Psychoeduc Assess 40:865–879. https://doi.org/10.1177/07342829221106585

110. Lee J (2009) Universals and specifics of math self-concept, math self-efficacy, and math anxiety across 41 PISA 2003 participating countries. Learn Individ Differ 19:355–365. https://doi.org/10.1016/j.lindif.2008.10.009

111. Lewis AJ, Rowland B, Tran A, et al (2017) Adolescent depressive symptoms in India, Australia and USA: Exploratory Structural Equation Modelling of cross-national invariance and predictions by gender and age. J Affect Disord 212:150–159. https://doi.org/10.1016/j.jad.2017.01.020

112. Li H, Ang RP, Lee J (2008) Anxieties in Mainland Chinese and Singapore Chinese adolescents in comparison with the American norm. J Adolesc 31:583–594. https://doi.org/10.1016/j.adolescence.2007.10.003

113. Li Q (2008) A cross-cultural comparison of adolescents’ experience related to cyberbullying. Educational Research 50:223–234. https://doi.org/10.1080/00131880802309333

114. Li J Bin, Delvecchio E, Di Riso D, et al (2015) Self-esteem and its association with depression among Chinese, Italian, and Costa Rican adolescents: A cross-cultural study. Pers Individ Dif 82:20–25. https://doi.org/10.1016/j.paid.2015.02.036

115. Li J Bin, Delvecchio E, Di Riso D, et al (2016) The Parent-Version of the Spence Children’s Anxiety Scale (SCAS-P) in Chinese and Italian Community Samples: Validation and Cross-Cultural Comparison. Child Psychiatry Hum Dev 47:369–383. https://doi.org/10.1007/s10578-015-0572-9

116. Li J Bin, Salcuni S, Delvecchio E (2019) Meaning in life, self-control and psychological distress among adolescents: A cross-national study. Psychiatry Res 272:122–129. https://doi.org/10.1016/j.psychres.2018.12.033

117. Liu X, Huang Y, Liu Y (2018) Prevalence, distribution, and associated factors of suicide attempts in young adolescents: School-based data from 40 low-income and middle-income countries. PLoS One 13:. https://doi.org/10.1371/journal.pone.0207823

118. Liu M wei, Chen Q tong, Towne SD, et al (2020) Fruit and vegetable intake in relation to depressive and anxiety symptoms among adolescents in 25 low- and middle-income countries. J Affect Disord 261:172–180. https://doi.org/10.1016/j.jad.2019.10.007

119. Lukoševičiūtė J, Gariepy G, Mabelis J, et al (2022) Single-Item Happiness Measure Features Adequate Validity Among Adolescents. Front Psychol 13:. https://doi.org/10.3389/fpsyg.2022.884520

120. Malykh S, Belova A, Sabirova E, et al (2013) Depressiveness in Children and Adolescents: A Cross-cultural Study in Russia and Kyrgyzstan. Procedia Soc Behav Sci 86:53–58. https://doi.org/10.1016/j.sbspro.2013.08.524

121. Mancinelli E, Liberska HD, Li J Bin, et al (2021) A cross-cultural study on attachment and adjustment difficulties in adolescence: the mediating role of self-control in italy, spain, china, and poland. Int J Environ Res Public Health 18:. https://doi.org/10.3390/ijerph18168827

122. Marksteiner T, Janson MP, Beißert H (2020) Belonging as Compensator: Social Belonging Moderates the Relation between Bullying and Well-Being Worldwide. Z Entwicklungspsychol Padagog Psychol 52:116–126. https://doi.org/10.1026/0049-8637/a000221

123. McCabe MP, Fuller-Tyszkiewicz M, Mellor D, et al (2012) Body satisfaction among adolescents in eight different countries. J Health Psychol 17:693–701. https://doi.org/10.1177/1359105311425274

124. McKinnon B, Gariépy G, Sentenac M, Elgar FJ (2016) Adolescent suicidal behaviours in 32 low- and middle-income countries. Bull World Health Organ 94:340-350F. https://doi.org/10.2471/BLT.15.163295

125. Medina CO, Jegannathan B, Dahlblom K, Kullgren G (2012) Suicidal expressions among young people in Nicaragua and Cambodia: A cross-cultural study. BMC Psychiatry 12:. https://doi.org/10.1186/1471-244X-12-28

126. Nansel TR, Craig W, Overpeck MD, et al (2004) Cross-national consistency in the relationship between bullying behaviors and psychosocial adjustment. Arch Pediatr Adolesc Med 158:730–736. https://doi.org/10.1001/ARCHPEDI.158.8.730

127. Nguyen HTM, Nguyen H V., Zouini B, et al (2022) The COVID-19 Pandemic and Adolescents’ Psychological Distress: A Multinational Cross-Sectional Study. Int J Environ Res Public Health 19:. https://doi.org/10.3390/ijerph19148261

128. Ojala K, Vereecken C, Välimaa R, et al (2007) Attempts to lose weight among overweight and non-overweight adolescents: a cross-national survey. International Journal of Behavioral Nutrition and Physical Activity 4:50. https://doi.org/10.1186/1479-5868-4-50

129. Page RM, Yanagishita J, Suwanteerangkul J, et al (2006) Hopelessness and loneliness among suicide attempters in school-based samples of Taiwanese, Philippine and Thai adolescents. Sch Psychol Int 27:583–598. https://doi.org/10.1177/0143034306073415

130. Page RM, Dennis M, Lindsay GB, Merrill RM (2010) Psychosocial Distress and Substance Use Among Adolescents in Four Countries. http://dx.doi.org/101177/0044118X10368932 43:900–930. https://doi.org/10.1177/0044118X10368932

131. Page RM, Saumweber J, Hall PC, et al (2013) Multi-country, cross-national comparison of youth suicide ideation: Findings from Global School-based Health Surveys. Sch Psychol Int 34:540–555. https://doi.org/10.1177/0143034312469152

132. Pat-Horenczyk R, Qasrawi R, Lesack R, et al (2009) Posttraumatic symptoms, functional impairment, and coping among adolescents on both sides of the israeli-palestinian conflict: A cross-cultural approach. Applied Psychology 58:688–708. https://doi.org/10.1111/j.1464-0597.2008.00372.x

133. Pfoertner TK, Rathmann K, Elgar FJ, et al (2014) Adolescents’ psychological health complaints and the economic recession in late 2007: a multilevel study in 31 countries. Eur J Public Health 24:961–967. https://doi.org/10.1093/EURPUB/CKU056

134. Pronk J, Lee NC, Sandhu D, et al (2017) Abociations between Dutch and Indian adolescents’ bullying role behavior and peer-group status. Int J Behav Dev 41:735–742. https://doi.org/10.1177/0165025416679743

135. Rajmil L, Herdman M, Ravens-Sieberer U, et al (2014) Socioeconomic inequalities in mental health and health-related quality of life (HRQOL) in children and adolescents from 11 European countries. Int J Public Health 59:95–105. https://doi.org/10.1007/S00038-013-0479-9

136. Ravens-Sieberer U, Erhart M, Gosch A, Wille N (2008) Mental health of children and adolescents in 12 European countries-results from the European KIDSCREEN study. Clin Psychol Psychother 15:154–163. https://doi.org/10.1002/cpp.574

137. Rescorla L, Achenbach TM, Ivanova MY, et al (2007) Epidemiological Comparisons of Problems and Positive Qualities Reported by Adolescents in 24 Countries. J Consult Clin Psychol 75:351–358. https://doi.org/10.1037/0022-006X.75.2.351

138. Rodríguez-Hidalgo AJ, Mero O, Solera E, et al (2020) Prevalence and psychosocial predictors of cyberaggression and cybervictimization in adolescents: A Spain-Ecuador transcultural study on cyberbullying. PLoS One 15:. https://doi.org/10.1371/journal.pone.0241288

139. Ruchkin V, Schwab-Stone M, Jones S, et al (2005) Article Is Posttraumatic Stress in Youth a Culture-Bound Phenomenon? A Comparison of Symptom Trends in Selected U.S. and Russian Communities

140. Ruchkin V, Sukhodolsky DG, Vermeiren R, et al (2006) Depressive symptoms and associated psychopathology in urban adolescents: A cross-cultural study of three countries. Journal of Nervous and Mental Disease 194:106–113. https://doi.org/10.1097/01.nmd.0000198142.26754.18

141. Samara M, Foody M, Göbel K, et al (2019) Do cross-national and ethnic group bullying comparisons represent reality? Testing instruments for structural equivalence and structural isomorphism. Front Psychol 10:. https://doi.org/10.3389/fpsyg.2019.01621

142. Sentenac M, Gavin A, Gabhainn SN, et al (2013) Peer victimization and subjective health among students reporting disability or chronic illness in 11 Western countries. Eur J Public Health 23:421–426. https://doi.org/10.1093/EURPUB/CKS073

143. Shapka JD, Onditi HZ, Collie RJ, Lapidot-Lefler N (2018) Cyberbullying and Cybervictimization Within a Cross-Cultural Context: A Study of Canadian and Tanzanian Adolescents. Child Dev 89:89–99. https://doi.org/10.1111/CDEV.12829

144. Shukla M, Wu AFW, Lavi I, et al (2022) A network analysis of adolescent mental well-being during the coronavirus pandemic: Evidence for cross-cultural differences in central features. Pers Individ Dif 186:. https://doi.org/10.1016/j.paid.2021.111316

145. Sitnikova MA, Ekaterina P, Alena D, et al (2022) The Cross-Cultural Differences in Perceived Stress of the COVID-19 Pandemic in Schoolchildren from Russia and Kyrgyzstan With Normal and High Levels of Anxiety and Depression. International Journal of Cognitive Research in Science, Engineering and Education 10:27–37. https://doi.org/10.23947/2334-8496-2022-10-2-27-37

146. Skinner AT, Gurdal S, Chang L, et al (2022) Dyadic Coping, Parental Warmth, and Adolescent Externalizing Behavior in Four Countries. J Fam Issues 43:237–258. https://doi.org/10.1177/0192513X21993851

147. Šmigelskas K, Vaičiūnas T, Lukoševičiūtė J, et al (2018) Sufficient social support as a possible preventive factor against fighting and bullying in school children. Int J Environ Res Public Health 15:e15050870–e15050870. https://doi.org/10.3390/ijerph15050870

148. Springer A, Kelder S, Orpinas P, Baumler E (2007) A cross-national comparison of youth risk behaviors in Latino secondary school students living in El Salvador and the USA. Ethn Health 12:69–88. https://doi.org/10.1080/13557850601002155

149. Stoet G, Bailey DH, Moore AM, Geary DC (2016) Countries with higher levels of gender equality show larger national sex differences in mathematics anxiety and relatively lower parental mathematics valuation for girls. PLoS One 11:. https://doi.org/10.1371/journal.pone.0153857

150. Sujoldzić A, De Lucia A (2007) A Cross-Cultural Study of Adolescents-BMI, Body Image and Psychological Well-Being

151. Tahmouresi N, Bender C, Schmitz J, et al (2014) Similarities and Differences in Emotion Regulation and Psychopathology in Iranian and German School-children: A Cross-cultural Study

152. Tang JJ, Yu Y, Wilcox HC, et al (2020) Global risks of suicidal behaviours and being bullied and their association in adolescents: School-based health survey in 83 countries. EClinicalMedicine 19:100253–100253. https://doi.org/10.1016/j.eclinm.2019.100253

153. Thorsén F, Antonson C, Palmér K, et al (2022) Associations between perceived stress and health outcomes in adolescents. Child Adolesc Psychiatry Ment Health 16:. https://doi.org/10.1186/S13034-022-00510-W

154. Toro J, Gomez-Peresmitré G, Sentis J, et al (2006) Eating disorders and body image in Spanish and Mexican female adolescents. Soc Psychiatry Psychiatr Epidemiol 41:556–565. https://doi.org/10.1007/s00127-006-0067-x

155. Turner-Moore T, Milnes K, Gough B (2022) Bullying in Five European Countries: Evidence for Bringing Gendered Phenomena Under the Umbrella of ‘Sexual Bullying’ in Research and Practice. Sex Roles 86:89–105. https://doi.org/10.1007/s11199-021-01254-1

156. Tuttle J, Gimenez G, Barrado B (2023) The Societal Context of School-Based Bullying Victimization: An Application of Institutional Anomie Theory in a Cross-National Sample. J Sch Violence 22:28–43. https://doi.org/10.1080/15388220.2022.2126850

157. Vancampfort D, Stubbs B, Firth J, et al (2018) Sedentary behavior and depressive symptoms among 67,077 adolescents aged 12-15 years from 30 low- and middle-income countries. International Journal of Behavioral Nutrition and Physical Activity 15:. https://doi.org/10.1186/s12966-018-0708-y

158. Vazsonyi AT, Ksinan A, Mikuška J, Jiskrova G (2015) The Big Five and adolescent adjustment: An empirical test across six cultures. Pers Individ Dif 83:234–244. https://doi.org/10.1016/j.paid.2015.03.049

159. Verhulst FC, Achenbach TM, van der Ende J, et al (2003) Comparisons of Problems Reported by Youths From Seven Countries

160. Vermeiren R, Deboutte D, Ruchkin V, Schwab-Stone M (2002) Antisocial behaviour and mental health: Findings from three communities. Eur Child Adolesc Psychiatry 11:168–175. https://doi.org/10.1007/s00787-002-0275-1

161. Vittetoe K, Lopez MF, Delva J, et al (2002) Behavioral problems and tobacco use among adolescents in Central America and the Dominican Republic

162. Volk AA, Provenzano DA, Farrell AH, et al (2021) Personality and bullying: Pathways to adolescent social dominance. Current Psychology 40:2415–2426. https://doi.org/10.1007/s12144-019-00182-4

163. Vore D, Banbury S, Lusher J (2016) Cognitive and stress vulnerabilities towards obsessive-compulsive disorder amongst British, Iranian and Lithuanian adolescents. Int J Cult Ment Health 9:303–312. https://doi.org/10.1080/17542863.2016.1170864

164. Wang MH, Xiao DM, Liu MW, et al (2020) Relationship between sedentary behaviour and anxiety symptoms among youth in 24 low- And middle-income countries. PLoS One 15:. https://doi.org/10.1371/journal.pone.0241303

165. Weine AM, Phillips JS, Achenbach TM (1995) Behavioral and Emotional Problems Among Chinese and American Children: Parent and Teacher Reports for Ages 6 to 13

166. Weitkamp K, Seiffge-Krenke I (2019) The Association Between Parental Rearing Dimensions and Adolescent Psychopathology: A Cross-Cultural Study. J Youth Adolesc 48:469–483. https://doi.org/10.1007/S10964-018-0928-0

167. Yuan Z, Tan J, Ye R (2023) A Cross-national Study of Mathematics Anxiety. Asia-Pacific Education Researcher 32:295–306. https://doi.org/10.1007/s40299-022-00652-7

168. Zgambo M, Kalembo FW elcome, Wang H, et al (2015) Prevalence and predictors of clinically significant depressive symptoms among Chinese and Malawian children: a cross-cultural comparative cross-sectional study. Glob J Health Sci 7:59–68. https://doi.org/10.5539/gjhs.v7n1p59

169. Zhang X, Slobodskaya HR, Kaneko H (2023) Adolescent mental health in Japan and Russia: The role of body image, bullying victimisation and school environment. International Journal of Psychology. https://doi.org/10.1002/IJOP.12947

170. Zhao Y, Ding C (2019) The association between students mathematic knowledge and factors related to students, parents, and school: A cross-cultural comparison study. Int J Educ Res 93:210–217. https://doi.org/10.1016/j.ijer.2018.11.006

171. Zhou Q, Lengua LJ, Wang Y (2009) The Relations of Temperament Reactivity and Effortful Control to Children’s Adjustment Problems in China and the United States. Dev Psychol 45:724–739. https://doi.org/10.1037/a0013776

172. Zietz S, Lansford JE, Liu Q, et al (2022) A longitudinal examination of the family stress model of economic hardship in seven countries. Child Youth Serv Rev 143:. https://doi.org/10.1016/j.childyouth.2022.106661
